# Biopolymers for Liver Tissue Engineering: A Systematic Review

**DOI:** 10.3390/gels11070525

**Published:** 2025-07-07

**Authors:** John Ong, Jacky Junzhe Zhao, Carla Swift, Athina E. Markaki

**Affiliations:** 1 Cambridge Stem Cell Institute, University of Cambridge, Cambridge CB2 0AW, UK; 2 Bedfordshire Hospitals NHS Trust, Bedford Hospital, Bedford MK42 9DJ, UK; carla.swift1@nhs.net; 3 Duke-NUS Medical School, Singapore 169857, Singapore; jz406@cantab.ac.uk; 4 Department of Engineering, University of Cambridge, Cambridge CB2 1PZ, UK; am253@cam.ac.uk

**Keywords:** biopolymers, hydrogels, scaffolds, substrates, stem cells, hepatocytes, cholangiocytes, organoids, tissue engineering, regenerative medicine, hepatology

## Abstract

Stem cell-derived liver cells, organoids, and lab-grown liver tissue are promising regenerative therapies for liver disease. However, current culture conditions are sub-optimal, producing end-target cells and tissue phenotypes that are immature or unstable when compared to primary liver cells and tissue. Biopolymers used in culture substrates and scaffolds for tissue engineering significantly impact the quality of the end-target cells and tissue, influencing the efficacy of regenerative treatments. In addition, the biochemical properties of some biopolymers may preclude the translation of downstream bioengineered products into clinical practice. Therefore, this systematic review aims to evaluate the recent advances in biopolymers within liver tissue engineering, providing an overview of the current usage in the field and highlighting novel substrates that have strong potential to be translated into clinical therapy.

## 1. Introduction

The incidence of liver disease is increasing globally [1]. It is the second most common cause of premature deaths in Europe, America, and Africa, and the third most common in Southeast Asia [2]. Over time, chronic liver disease can lead to liver cirrhosis and liver failure. At present, the only cure for end-stage liver failure is liver transplantation; however, the patient demand far exceeds the donor supply. In the United Kingdom alone, patients wait an average of 3 to 4 months for a liver transplant for chronic liver failure [3]. Unfortunately, approximately 10% of these patients die while on the waiting list due to the complications of their liver disease, and an even greater number are turned down for transplantation [4,5]. Therefore, novel therapies for the treatment of liver failure are urgently needed.

Bioengineering liver tissue from human stem cells and organoids offers excellent potential as an alternative to conventional liver transplantation. Vast quantities of cells and/or tissue could be generated in vitro, relieving the strain on the limited donor pool. In addition, hepatocytes, cholangiocytes, or liver organoids derived from patient-specific stem cells enable the autologous transplantation of end-target cells or tissue. This negates the need for long-term immune suppression and mitigates its associated side effects, which are often encountered with non-autologous liver transplantation. For example, Japanese researchers have successfully transplanted hepatocytes derived from human embryonic stem cells into an infant with urea cycle disorder, temporarily reversing the disease so that a liver transplant could be performed safely five months later [6]. Apart from cell therapy or tissue transplantation, regenerative approaches can also be used for disease modelling and predicting the response to novel drug therapies more accurately [7,8,9]. It is noteworthy that approximately half of the drugs that have caused drug-induced liver injury in humans did not have any significant liver-toxic effects during animal testing [10]. In contrast, stem cell-derived liver models, such as bioengineered liver tissue or liver-on-a-chip devices, have demonstrated good concordance between in vitro and in vivo drug toxicity, making them of strong interest to the pharmaceutical industry [11].

Despite the immense potential of stem cell-derived liver cells and tissue, it is widely recognised that current culture conditions are sub-optimal and yield immature end-target cells compared to primary cells [12,13]. A key factor attributed to this shortcoming is the lack of suitable biomaterials that accurately reproduce the stem cell niche during liver differentiation, both in terms of composition and dimensionality [14,15]. Three-dimensional (3D) systems and tissue-specific matrices yield better cellular phenotypes than two-dimensional (2D) culture systems and generic substrates [16,17,18,19]. However, the substrates commonly used in 3D culture systems, like Matrigel, are usually xenogenic and associated with many problems. For example, the concentrations of growth factors such as epidermal growth factor, insulin growth factor 1, and fibroblast growth factor 2 are inconsistent between Matrigel batches [20,21]. In addition, growth factor reduced versions of Matrigel contain only 53% of the extracellular matrix (ECM) proteins found in Matrigel [22]. These inconsistencies cause significant biochemical and mechanical variations [23], leading to poorly reproducible in vitro results [20,21,22,23,24]. Furthermore, poor definability, immunogenicity [25,26], and pathogenicity [27,28] preclude its use and any downstream products from clinical therapy [29].

**Figure 1:**
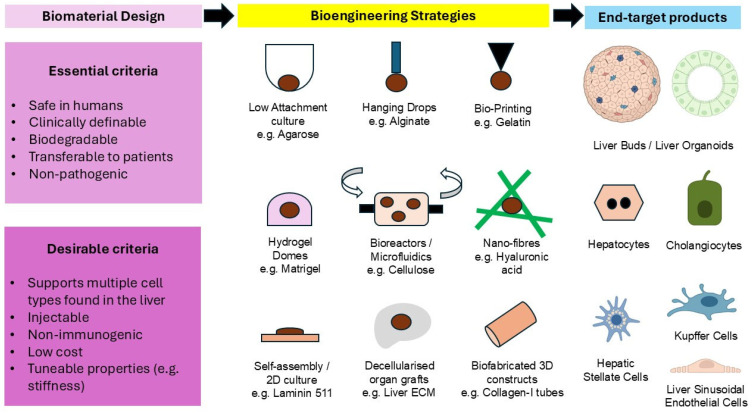
Key areas in biomaterial design, current bioengineering strategies, and desired end-target products of liver bioengineering. Dark brown areas under bioengineering strategies indicate cell masses.

Therefore, novel and better biomaterials are urgently needed to help achieve bioengineered and regenerative products to treat liver disease. Figure 1 provides a schematic of the biomaterial design and current strategies in liver bioengineering. The primary objective of this focused systematic review was to study the biomaterials used in liver bioengineering between 2020 and 2025, thereby understanding the direction of future practices. The secondary objective was to appraise novel biomaterials for their early potential in clinical translation, focusing on definability, versatility, degradability, and biosafety. It is essential to note that different types of stem cells exhibit distinct responses to the various biopolymers within culture substrates and scaffolds based on their degree of stemness and their inherent propensity to hepatic differentiation. Therefore, in the context of this review, we have classified the various types of stem cells broadly into three categories: (i) pluripotent stem cells (PSCs), (ii) liver stem cells (LSCs), and (iii) non-liver stem cells (NLSCs). PSCs refer to human induced pluripotent stem cells (iPSCs) and embryonic stem cells (ESCs). LSCs refer to native human hepatic or biliary human progenitor stem cells, such as LGR5+ hepatoblasts. NLSCs refer to all other human non-pluripotent and non-liver stem cells, e.g., mesenchymal stem cells (MSCs).

Herein, we present a comprehensive summary of biomaterial usage within human liver bioengineering, demonstrating that xenogenic matrices are still widely used. However, we have found that chemically defined alternatives are being increasingly explored, with recombinant human proteins gaining popularity in routine laboratory practice. Finally, we discuss the advantages and disadvantages of these alternatives and provide insights into the future direction of biomaterials research.

## 2. Results and Discussion

### 2.1. Summative Results of the Systematic Searches

**Figure 2:**
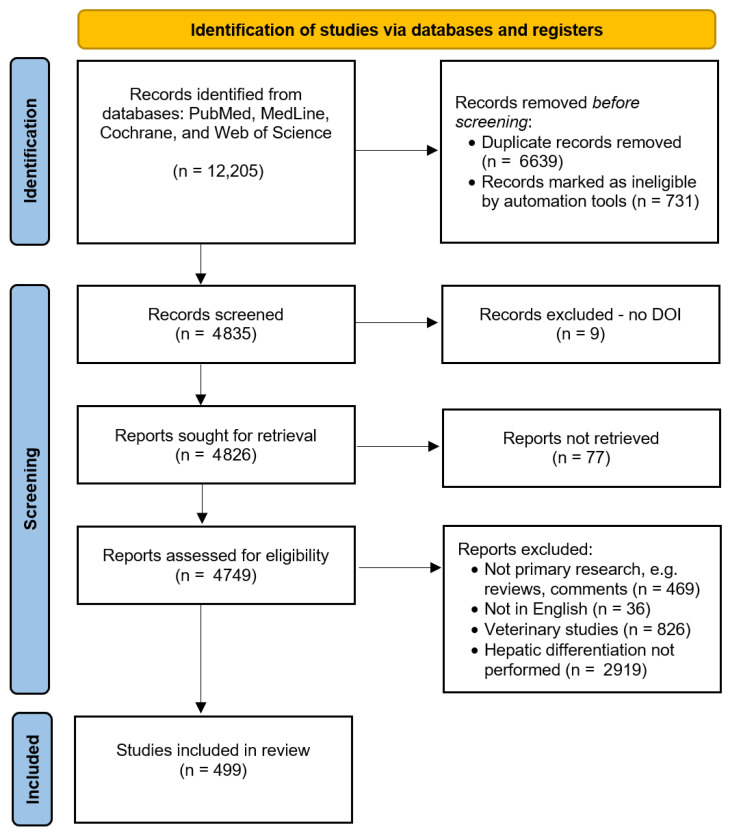
PRISMA 2020 systematic review flow diagram, adapted from Page, M.J. et al., BMJ 2021 [30].

Four public databases were comprehensively searched for articles published between 1 April 2020 and 1 April 2025 that reported human stem cell differentiation into liver cells, organoids, or tissue (the systematic search strategy is described in Section 4). A total of 12,205 articles were identified. After removing duplicates and ineligible hits (*n* = 7370), 4835 articles were screened for eligibility. During the screening process, 4336 articles were excluded from further analyses, and 499 were included after meeting the inclusion criteria (selection criteria described in Section 4). Figure 2 illustrates the PRISMA systematic review flow diagram. Of these 499 articles, 282 used PSCs, 158 used LSCs, and 59 used NLSCs for liver bioengineering. The results from each stem cell group are reported separately below.

### 2.2. Biomaterials Used to Derive Liver Cells and Organoids from PSCs

Of the 282 studies that used PSCs (detailed in Supplementary Table S1), eight reported cholangiocyte differentiation, 197 reported hepatocyte differentiation, 86 reported liver organoid culture, two reported differentiation of hepatic stellate cells, and two reported the differentiation of liver sinusoidal endothelial cells (LSEC). In total, 10 studies differentiated PSCs into hepatocytes AND cholangiocytes/liver organoids. Overall, 75.2% (212/282) of these studies used Matrigel or its derivatives for maintenance or differentiation. The other types of biopolymers used are summarised in Table 1.

**Figure 3:**
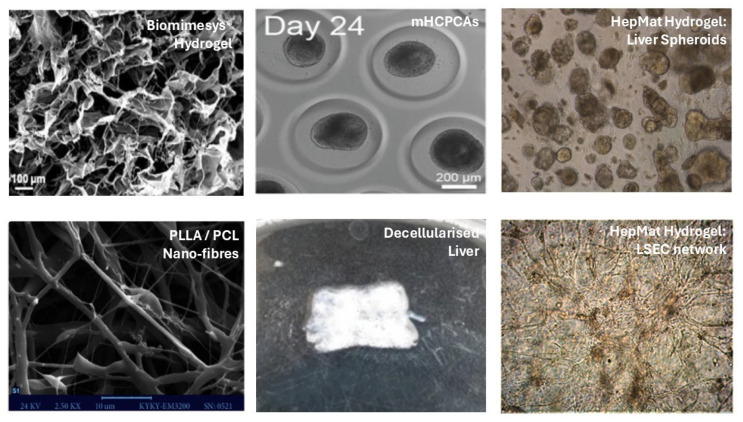
Liver bioengineering strategies for deriving liver tissue from PSCs. Images adapted from original articles: Biomimesys [31], agarose-based mHCPCAs = microfabricated hexagonal closely packed cavity arrays [32], HepMat, LSEC = liver sinusoidal endothelial cells [33], PLLA/PCL nano-fibres [34], and decellularised rabbit liver [35].

In total, 100% (8/8) of the PSC studies reporting cholangiocyte culture used Matrigel or its derivatives (growth factor reduced Matrigel). In 50% (4/8) of studies, collagen-I was added to Matrigel derivatives for cholangiocyte differentiation. Of the PSC studies reporting hepatocyte culture, 71.1% (140/197) used Matrigel or its derivatives, 13.7% (27/197) used laminin 511 or its derivatives (laminin 511-silk), and 10.7% (21/197) used laminin 521 or its derivatives (laminin 521-silk). The remaining studies used cellulose, Cellartis definitive endoderm differentiation coating, Cellartis DEF-COAT 1, PEG-peptides (e.g., “HepMat”), gelatine, fibronectin, liver scaffolds/ECM, Hydrox^TM^ coating (a peptide-functionalized hydrogel), Synthemax^®^ II coating (peptides covalently bonded to an acrylate polymer backbone)**,** placental ECM, vitronectin, PAA, PLLA/PCL, PCL-Gel-HA, and suspension culture. For three-dimensional (3D) liver and/or organoid culture, 93.4% (71/86) used Matrigel or its derivatives. The remaining studies used Biomimesys^®^ (HCS Pharma, Loos, France) (a hyaluronic acid-based scaffold containing collagen, laminin, and fibronectin—discussed below), gelatine, GelMA, decellularised animal livers, laminin 521, or PEG. Figure 3 illustrates some of the novel 3D strategies for liver bioengineering using PSCs.

Notably, researchers have also used specific biopolymers that do not deliver chemical cues to PSCs. For example, agarose-based microfabricated hexagonal closely packed cavity arrays (mHCPCAs), as pictured above, have been explored as a suspension culture method [32,36]. The hypothesis is that the tightly controlled microenvironment created by the mHCPCAs produces significantly less heterogeneity in organoid size, morphology, and maturation rate than standard Matrigel domes. Nonetheless, the liver organoids produced by this method are still noted to be immature [32,36] compared to primary human hepatocytes. Agarose is hydrophobic and lacks cell-binding motifs, promoting suspension culture, spheroid, or organoid formation [37]. The lack of mechano- and chemical cues most likely explains these results and highlights the importance of bioactive biopolymers within scaffolds.

### 2.3. Biomaterials Used to Derive Liver Cells and Organoids from LSCs

Of the 158 studies that used LSCs (detailed in Supplementary Table S2), three reported cholangiocyte differentiation, 24 reported hepatocyte differentiation, 48 reported biliary organoid culture, and 89 reported liver organoid culture. Five studies differentiated LSCs into hepatocytes and cholangiocytes/liver organoids. In total, 76.4% (120/157) of all these studies used Matrigel or its derivatives for maintenance or differentiation. The other types of biopolymers used are summarised in Table 2.

**Figure 4:**
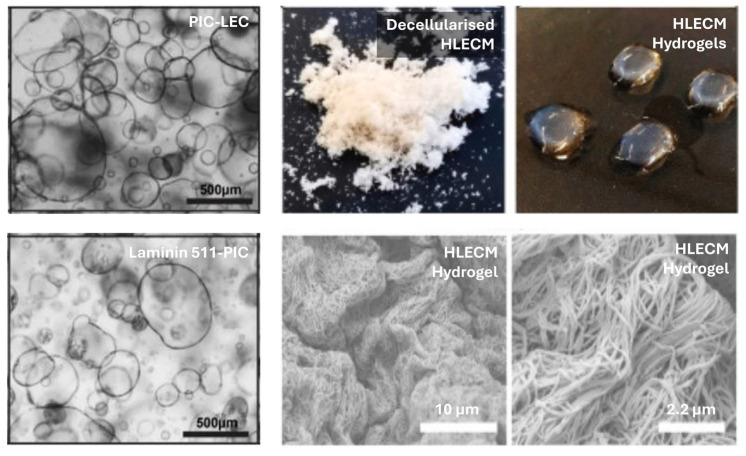
Left panel: Successful liver organoid culture in laminin 511-PIC and PIC-LEC, adapted from [38]. Centre and right panels: HLECM = human liver ECM from decellularised human liver made into a hydrogel, adapted from [39].

In total, 66.6% (2/3) of the studies reporting cholangiocyte differentiation from LSCs used Matrigel or its derivatives (growth factor reduced Matrigel); the remaining study used collagen-I. Of the studies reporting hepatocyte differentiation from LSCs, 18.2% (4/22) used Matrigel or its derivatives. An identical proportion (4/22) used collagen-I, while 63.6% (14/22) of studies did not use any substrate, as LSCs appear to be adherent cells in two-dimensional (2D) cell cultures. In contrast, 93.9% (46/49) of studies used Matrigel or its derivatives for 3D biliary organoid culture; the remaining used collagen-I, hyaluronic acid, liver ECM, or suspension culture. For 3D hepatic organoid culture, 85.4% (76/89) used Matrigel or its derivatives. The remaining studies used Hydrox^TM^, laminin 332, GelMA, PEG, PIC-laminin 511, suspension culture methods, or decellularised liver grafts. Figure 4 illustrates some of the interesting results from these studies.

### 2.4. Biomaterials Used to Derive Liver Cells and Organoids from NLSCs

Of the 59 studies that used NLSCs (detailed in Supplementary Table S3), one reported cholangiocyte differentiation, 52 reported hepatocyte differentiation, one reported liver sinusoidal endothelial cell culture, and five reported liver organoid culture. In total, 3.4% (2/59) of all these studies used Matrigel or its derivatives for maintenance or differentiation, 18.6% (11/59) used collagen-I as a substrate, and 55.9% (33/29) used no substrate. The other types of biopolymers used are summarised in Table 3.

The only study that reported cholangiocyte differentiation from NLSCs used collagen-I. Of the studies that reported hepatocyte differentiation, 63.5% (33/52) did not use any substrate, 23.1% (12/52) used collagen-I or gelatine, and only one used Matrigel (growth factor reduced). Fibronectin was used for the only study that differentiated NLSCs into LSECs. For 3D liver organoid culture, collagen-I (2/5), agarose (1/5), Lipidure coating (1/5), and Wharton’s jelly (1/5) were used as substrates.

### 2.5. Discussion of Results

PSCs are the most attractive cell source for bioengineering liver tissue because of their potential to differentiate into all the other cell types in the human liver, e.g., HSCs, immune cells, LSECs, etc. However, the stemness of PSCs places greater importance on the biopolymers in the in vitro culture environment because these function as cues for directed differentiation and maturation towards the intended end-targets. In addition, the versatility of a substrate is crucial for its uptake in standard laboratory practices and clinical therapy. Ideal substrates should support the differentiation of stem cells into all the cell types of the target organ, facilitate the different phases of differentiation without cell dissociation, and avoid replating for substrate changes. Substrates should also be easily transferable into humans, e.g., injectable or implantable. This study has identified several fully defined biomaterials used as an alternative to Matrigel-derived substrates in recent years. The properties of these biomaterials are briefly summarised in Table 4.

Collagen-I is frequently used in 2D hepatocyte and cholangiocyte differentiation. Nonetheless, it is widely recognised that it promotes the undesired spontaneous differentiation of PSC and is sub-optimal for hepatic organoid culture. Thus, conjugation with other biomaterials is necessary; Matrigel can be added to collagen-I to support liver organoid culture (Supplementary Table S1). Notably, some groups have biofabricated collagen-I constructs that serve as scaffolds for cell or organoid delivery rather than platforms for in vitro tissue culture [40].

Hyaluronic acid (HA) was featured in two studies: one study differentiated PSCs into hepatocytes using Biomimesys^®^—HA conjugated to collagen-I and collagen-IV via RGD binding sites [31]. The other study used HA conjugated with cholesterol and dexamethasone to maintain and differentiate LSCs into biliary organoids [41]. Roudaut et al. [31] reported that HA sheets modulated the porosity of collagen fibres in Biomimesys^®^, resulting in pore sizes ranging between 50 μm and 200 μm. The elastic modulus was 0.15  ±  0.05 kPa with a swelling ratio of 60  ±  10 g/g; the hydrogel can absorb roughly 60 times its dry weight in water [31]. These measurements suggest that Biomimesys^®^ is more conducive to liver organoid culture since Broguiere et al. [42] reported that the pore size of Matrigel used for liver organogenesis is below 200 nm; a large pore size improves the perfusion and diffusion within hydrogels. The stiffness of Matrigel, which varies between 0.2–0.84 kPa [23], is also comparable to Biomimesys^®^. In evaluating the liver organoids derived in Biomimesys^®^, the expression of key markers, including but not limited to albumin and hepatocyte nuclear factor 4 (HNF4), suggested the successful derivation of hepatocytes, whilst SRY-box transcription factor 9 (SOX-9) and cystic fibrosis transmembrane conductance regulator (CFTR) expression suggested the successful derivation of cholangiocytes within the liver organoid. Notably, the expression profiles of apolipoprotein B, zonula occludens-1, cytochrome P450 (CYP) 3A4, 1A2, 2C9, 2D6, and 2B6 with drug exposure, and significant lipid metabolism also served as essential features of hepatocyte maturity. However, it is noteworthy that key comparisons were lacking. These results would have been more insightful and informative if comparisons were made to primary human hepatocytes, especially since the authors report a decline in liver organoid function after 35 days. The differentiation efficiency was also not reported. Future murine experimentation to evaluate the biosafety, biodegradability, and engraftment potential of Biomimesys^®^ in vivo is also desired. In contrast to Roudaut et al. [31], Di Matteo et al. [41] biofabricated HA–cholesterol–dexamethasone nano-hydrogels for drug delivery to liver stem cells instead of using hydrogels as bioactive scaffolds. They showed that the phenotype of cholangiocyte-like cells derived from the liver stem cells could be enhanced with increased expression of the Na^+^/H^+^ exchanger isoform 1 (NHE1). Interestingly, when injected into mice, these drug-loaded nano-hydrogels also seemed to reduce liver injury in the mouse model. However, it is unclear whether this is achieved through liver stem cells. Nonetheless, HA is already widely used in medicinal products, and thus it is a promising biomaterial for liver tissue engineering. Further research is needed to determine whether HA-based matrices can support the multi-cell type differentiation required in liver organogenesis. Additional validation with in vivo data and clinical trials is also required.

A total of four studies used fibronectin to maintain stem cells in culture [43,44,45,46]. Four studies used it for hepatocyte differentiation [43,45,47,48]. One study used fibronectin to derive LSECs [46]. In all these studies, fibronectin was used as a 2D substrate. Indeed, fibronectin seems promising; however, in our experience it is highly viscous, which makes it very challenging to use in cell culture. In addition, only two of the above studies have reported results from short-term murine experiments. Therefore, more data, including from animal studies, are needed. Fibroin is considered a bioactive material [49]; however, it has only been used in one study with NLSCs [50]. In the study, the authors reported that the fibroin pores were large (≈300 μm), the scaffolds swelled up by 758.9%, and they degraded after 18 days in vitro. However, the Young’s modulus was 43.57 ± 2.34 kPa, suggesting that these scaffolds were as stiff as cirrhotic human livers [51]. Unfortunately, the characterisation of the hepatocyte-like cells cultured on fibroin was limited. Importantly, the authors reported that the alpha-fetoprotein (AFP) expression was relatively high compared to albumin, which suggests that these cells are still very immature. Direct comparisons to primary human hepatocytes were also not performed. Therefore, the potential of fibroin scaffolds remains undetermined.

Human recombinant laminins are an increasingly popular alternative to poorly defined xenogenic substrates, although laminin type significantly affects stem cell biology. Laminin 511 and laminin 521 are native to the developing embryo [52], and are the only two laminins that support the long-term clonal expansion of PSCs in vitro [53]. Laminin 111 and laminin 332 are associated with the epithelial-to-mesenchymal transition [54,55], especially in ESCs [54]. Therefore, on their own, they may not be suitable for long-term stem cell culture and/or liver differentiation. However, adding these laminins to laminin 511 or laminin 521 may be a valuable strategy in achieving better end-target phenotypes. Importantly, all pure forms of human recombinant laminins are only available in low-concentration aqueous solutions, which are of limited use in 3D liver bioengineering. As a result, several groups have attempted to conjugate laminins with various polymeric backbones to form hydrogels. Laminin 511-PIC [56], PIC-LEC [57], laminin 511-silk (Supplementary Tables S1 and S2), and laminin 521 (Biosilk) are recent attempts reported above. However, robust in vivo data on these biomaterials are still outstanding. Notably, at the time of writing, only aqueous laminin 511 (iMatrix) and laminin 521 (Biolamina) are available at good manufacturing practice (GMP) and clinical grades. Therefore, an essential area for development for 3D liver bioengineering is the development of clinical grade hydrogels (or scaffolds) incorporating laminin 511 or laminin 521 with supportive ECMs to enhance end-target phenotypes. Notably, laminin 411 has previously been reported to enhance liver differentiation [58,59,60]. Laminin 411-containing scaffolds and substrates, however, remain unexplored.

Among the hydrogels biofabricated with laminins identified in this review, PIC-LEC hydrogels exhibit a very low storage modulus and, consequently, with Young’s moduli in the range of several tens of Pa, they are sufficiently compliant to support the growth of liver organoids. Their porosity is dependent on the PIC and LEC concentrations used in the hydrogel synthesis, and remains poorly characterised [38]. Although lacking in vivo data, in vitro data showed that liver organoids cultured in PIC-LEC or PIC-laminin 511 had similar hepatocyte gene expression profiles (albumin, CYP3A4, and multidrug resistance protein 2) compared to Matrigel and cryopreserved human hepatocytes [38]. Functional analyses also showed that intracellular albumin, intracellular glutamate dehydrogenase, and excretory ammonia were similar to Matrigel. Though these results are promising, it is essential to note that cryopreserved human hepatocytes are not directly comparable to freshly isolated human hepatocytes because iatrogenic cell injury during cryopreservation could adversely affect cell function. Thus, future animal experimentation will be key in evaluating the biosafety, biodegradability, functionality, cell engraftment efficiency, and treatment efficacy of PIC-LEC/laminin 511 hydrogels in a liver failure model. Other data on laminin 521 and laminin 511 conjugates were lacking.

Our searches have also shown that synthetic alternatives such as PEG constructs have been increasingly explored. PEG is usually considered inert; thus, groups have conjugated PEG with bioactive polymers to deliver chemical cues to stem cells with some success. “HepMat”—PEG and RGD conjugates [33], and other PEG-peptide constructs [61] have been shown to support hepatocyte differentiation. Importantly, Kumar et al. [33] showed that HepMat can facilitate the derivation of both hepatocytes and LSECs from PSCs. Messenger ribonucleic acid (mRNA) copies of the hepatocyte markers HNF4-alpha, CYP3A4, CYP2D6, glucose-6-phosphatase, and peroxisome proliferator-activated receptor gamma coactivator 1-alpha (PGC1α) were similar in hepatocytes derived in HepMat versus cryopreserved human hepatocytes. Functional assays were limited but showed similar CYP3A4 activity (7-benzyloxy-4-trifluoromethylcoumarin fluorescence) compared to cryopreserved human hepatocytes. Notably, AFP was positive in HepMat-derived hepatocytes, indicating a degree of immaturity, but AFP quantification and comparisons were not provided. Separately, LSEC differentiation was evidenced by the upregulation of lymphatic vessel endothelial hyaluronan receptor 1 (LVEH-1) and CD31; macrophages and hepatic stellate cells were also generated from PSCs. However, in the latter, Matrigel was used instead of HepMat. The various cell types generated by the authors were used to create liver organoids that could model inflammation and fibrosis in fatty liver disease. This acts as an interesting model for testing novel anti-inflammatory and anti-fibrotic therapies. However, the porosity and stiffness data for HepMat are unavailable, and in vivo experiments were not performed. Similarly, Blackford et al. [61] attempted a 2D approach by conjugating PEG with RGD-containing peptides; however, the lack of suitable controls and in vivo data limits insight and comparison. Notably, these issues apply to most of the synthetic biomaterials listed in Table 4. It was also observed that synthetic polymers not conjugated with other bioactive polymers were mainly used as substrates to reduce cell adhesion and facilitate suspension culture, e.g., Lipidure, PLLA/PCL fibres, Hydrox^TM^ fibres, etc. Nonetheless, undisclosed proprietary information and non-comprehensive biofabrication methods significantly hinder the reproducibility and validation of these novel synthetic materials.

In addition to these challenges, regulatory factors are critical aspects of biomaterial design that can hinder the clinical translation of bioengineered products. It is prudent for researchers to be aware that regulatory bodies can evaluate (i) acellular biomaterials as either drugs or devices, and (ii) bioengineered liver constructs as either advanced therapy medicinal products (ATMPs, in Europe) and/or genetically modified organisms (GMOs). The latter attracts stricter regulatory control and scrutiny before approval for clinical use. The Food & Drug Administration (FDA, Silver Spring, MD, USA) has several definitions of a drug; the two definitions applicable to biomaterials are “A substance intended for use in the diagnosis, cure, mitigation, treatment, or prevention of disease.”, or “A substance (other than food) intended to affect the structure or any function of the body” [62]. Yet, some cases may not be straightforward, e.g., the FDA classifies HA as a medical device, although it seeks to reclassify it as a drug [63]. Nonetheless, prerequisites for regulatory scrutiny before clinical trials include: (i) the identification of the potential targets for pharmaceutical action by the drug, (ii) identification of the chemicals within the drug that modify the targets, (iii) animal and in vitro studies showing the efficacy and safety (including carcinogenicity, mutagenicity, and teratogenicity) of the drug, (iv) drug formulation and manufacturing processes (GMP compliant), and (v) drug purity and stability through the manufacturing process and over time [62]. If these checks are satisfactory, a biomaterial may be registered for phased clinical trials (phases 0 to 4). However, further checks may be required if stem cells are used with biomaterials. For example, iPSCs modified by viral vectors fall within the European framework of GMOs [64], and this may prolong the approval process. An approach to expedite the regulatory process is to use starting materials already approved for clinical use, such as clinical-grade recombinant proteins or pre-approved clinical-grade cells. For example, clinical-grade and GMP-compliant aqueous laminin 521 is commercially available (Biolamina, Sundbyberg, Sweden), and alginate hydrogels (FDA-approved) have been used to encapsulate primary human hepatocytes for transplantation into children with liver failure [65].

In summary, approximately 75% of the studies that bioengineer liver from PSCs and NLSCs continue to use Matrigel or its derivatives. A universal and “gold standard” biomaterial for liver bioengineering is still lacking. NLSCs are self-adherent cells that do not require bioactive substrates for liver differentiation but are limited by their lack of pluripotency. Novel techniques to derive other liver cell types from NLSCs can significantly improve liver tissue engineering. In addition, hydrogels and scaffolds made from recombinant human proteins are attractive biomaterials to enhance liver derivation from PSCs and LSCS in future research. To that end, hyaluronic acid-based hydrogels are promising candidates, but further research is needed for significant gains. Laminin 511 and laminin 521 are available as aqueous clinical-grade solutions only, and hydrogel development with in vivo validation is an important direction for future 3D liver bioengineering efforts and downstream clinical translation. Synthetic polymers are viable alternatives to “natural” polymers but remain underdeveloped. Notably, synthetic polymers have more milestones to achieve than human recombinant matrices, which have been more extensively researched.

This review was based on the 499 studies (detailed in Supplementary Tables S1–S3) meeting our selection criteria. However, a limitation of this review is that searches on Google and Scopus were not conducted because the sheer number of hits would have been too overwhelming to process. Therefore, studies not archived in PubMed, Web of Science, Medline, or Cochrane would not have been included in this review. Nonetheless, we believe the data presented herein is an accurate representation of the field.

## 3. Conclusions

Despite advances in biomaterial development, poorly defined xenogenic substrates remain the predominant biomaterials in liver bioengineering, hindering clinical translation. Current data suggests that human recombinant proteins can produce end-target phenotypes similar to Matrigel, but uptake within the field is slow despite being commercially available. Research into synthetic alternatives to Matrigel is active; however, in-depth characterisation, in vivo data, and wider validation are needed for progress. Strategies to advance the field of liver bioengineering are proposed within this review.

## 4. Materials and Methods

This review is reported in accordance with the recommendations from the PRISMA statement [66]. The review protocol is available from the authors upon reasonable request. The review has been registered with the Open Science Framework.

### 4.1. Data Sources and Searches

Electronic searches: A search of PubMed, Medline, Cochrane, and Web of Science databases was performed for articles published in the last five years between 1 April 2020 to 1 April 2025 using the search terms “stem cells” and “hepatocytes”, “stem cells” and “cholangiocytes”, “liver” and “stem cells”, “liver organoids”, “hepatic organoids”, “biliary organoids”, and “cholangiocyte organoids”. The UK Access Management Federation provided access to journals via Cambridge University. Searching in other resources: The Cambridge University Library was checked for articles that were listed in online search results but not accessible.

### 4.2. Study Selection

Inclusion criteria: Only (i) articles in English, (ii) articles with abstracts and full-text links for screening, and (iii) primary research articles were included. Exclusion criteria: Articles (i) not reporting primary research (e.g., reviews, comments, etc.), (ii) not written in English, (iii) lacking a DOI, web link, and not accessible by the UK Access Management Federation, were excluded. In addition, (iv) veterinary studies (e.g., animal cell lines and experimentation only) and (v) studies not reporting the differentiation of stem cells into liver cells, organoids, or tissue were also excluded.

### 4.3. Data Extraction and Quality Assessment

Three authors independently reviewed all titles and abstracts and then assessed articles against the inclusion criteria for analysis. A fourth reviewer resolved any differences. Data were extracted independently by each author using a standardised form. The recorded data included the biomaterials, the stem cell type, and the end-target cell or tissue type achieved. Outcomes of stem cell differentiation were recorded as end-target phenotypes: hepatocytes, cholangiocytes, hepatic organoids, or biliary organoids. Variables recorded were type of stem cell used, substrates used for stem cell maintenance, and substrates used for stem cell differentiation to achieve end-target phenotypes. All the above data were recorded in Supplementary Tables S1–S3. Risk of bias and quality assessment: All primary research articles that fulfilled the above criteria were screened, minimising the risk of publication bias. The risk of language bias is present but low.

### 4.4. Data Synthesis and Analysis

Statistical analysis: Data from articles that were successfully screened using the inclusion and exclusion criteria were extracted and tabulated in Microsoft Excel. Descriptive statistics were used to report data between the stem cell groups. Figures were created using Biorender (https://BioRender.com). Articles with missing data were omitted from the statistical analyses. Assessment of heterogeneity and publication bias was not applicable as meta-analyses were not performed.

## Figures and Tables

**Table 1:** Studies using biopolymers to bioengineer human liver from PSCs are summarised below and detailed in Supplementary Table S1.

Biopolymer/Substrate	Maintenance	Differentiation
Matrigel	112	155
Matrigel (GFR) ^1^	54	43
Biomimesys	-	1
Collagen-I	-	13
Cellulose	-	1
Cellartis DEF COAT-1	9	1
Cellartis DED coating ^2^	-	7
Decellularised liver ECM	-	3
Feeder cells	26	3
Fibronectin	-	2
Gelatine	5	13
GelMA ^3^	-	1
hE-cad-Fc	1	-
HepMat	-	1
Hydrox^TM^ coating	-	1
Laminin 111	-	1
Laminin 511	20	9
Laminin 511-Silk	6	6
Laminin 521	16	18
Laminin 521-Silk (Biosilk)	1	-
PEG-coated constructs ^4^	-	1
PEG-peptides	-	1
PLLA/PCL fibres ^5^	-	1
PCL-Gel-HA ^6^	-	1
Placenta ECM	1	2
PAA ^7^	-	1
Suspension Culture	1	11
Synthemax II coating	1	1
Vitronectin	35	2

^1^ GFR = growth factor reduced, ^2^ DED = definitive endoderm differentiation, ^3^ GelMA = gelatine methacryloyl, ^4^ PEG = polyethylene glycol, ^5^ PLLA/PCL = poly-L-lactic acid/poly (*ε*-caprolactone), ^6^ PCL-Gel-HA = poly *ε*-caprolactone-gelatine-hyaluronic acid, ^7^ PAA = polyacrylamide.

**Table 2:** Studies using biopolymers to bioengineer human liver from LSCs are summarised below and detailed in Supplementary Table S2.

Biopolymer/Substrate	Maintenance	Differentiation
Matrigel	90	96
Matrigel (GFR)	28	23
Collagen-I	14	11
Fibronectin	1	-
Gelatine	1	1
Gelatine-Alginate	-	1
GelMA	-	1
Hyaluronic Acid	1	1
Hydrox^TM^	-	1
Liver ECM	-	1
Laminin 332	-	1
Laminin 511-PIC ^1^	-	1
PIC-LEC ^2^	-	1
PEG	-	1
PCL	-	1
Suspension Culture	3	10
No coating	21	15

^1^ PIC = polyisocyanopeptides, ^2^ PIC-LEC = PIC-laminin 111-entactin complex.

**Table 3:** Studies using biopolymers to bioengineer human liver from NLSCs are summarised below and detailed in Supplementary Table S3.

Biopolymer/Substrate	Maintenance	Differentiation
Matrigel	1	-
Matrigel (GFR)	1	1
Agarose	-	1
Collagen-I	5	11
Fibronectin	3	2
Fibroin	-	1
Gelatine	3	4
Liver ECM (animal)	-	4
Lipidure coating ^1^	-	1
Wharton’s Jelly	1	1
No coating	45	33

^1^ Phosphorylcholine-based polymer.

**Table 4:** Non-Matrigel-derived biomaterials used to bioengineer human liver.

Biopolymers/ Synthetic Substrates	Bioactive	Fully Defined	Easily Transferable	Biodegradable In Vivo	Biosafety Studies
Collagen-I	✔	✔	✔	✔	✔
Hyaluronic Acid	✔	✔	✔	✔	✔
Laminin 111	✔	✔	✗	✔	✗
Laminin 332	✔	✔	✗	✔	✗
Laminin 511	✔	✔	✗	✔	✔
Laminin 521	✔	✔	✗	✔	✔
PIC-LEC	✔	✔	✔	✗	✗
Laminin 511-PIC	✔	✔	✔	✗	✗
Cellartis DEF COAT-1	✔	✔ *	✗	✗	✗
Cellartis DED Coating	✔	✔ *	✗	✗	✗
Fibronectin	✔	✔	✗	✗	✗
Fibroin	✔	✔	✗	✗	✗
hE-cad-Fc	✔	✔	✗	✗	✗
HepMat	✔	✔	✗	✗	✗
PEG-peptides	✔	✔	✗	✗	✗
PCL-Gel-HA	✔	✔	✗	✗	✗
Vitronectin	✔	✔	✗	✗	✗
Laminin 511-Silk	✔	✔	✗	✗	✗
Laminin 521-Silk	✔	✔	✗	✗	✗
Hydrox^TM^ coating	✗	✔	✗	✗	✗
Lipidure^®^ coating	✗	✔	✗	✗	✗
PCL	✗	✔	✗	✗	✗
PLLA/PCL fibres	✗	✔	✗	✗	✗
PEG-coated constructs	✗	✔	✗	✗	✗
PAA	✗	✔	✗	✗	✗
Synthemax II	✗	✔	✗	✗	✗
Cellulose	✗	✔	✗	✗	✗

✔ = data available, ✗ = no or weak evidence, * = composition undisclosed or proprietary.

## Data Availability

The original contributions presented in this study are included in the article/Supplementary Material. Further inquiries can be directed to the corresponding author.

## Supplementary Materials

The following supporting information can be downloaded at: https://www.mdpi.com/article/10.3390/gels11070525/s1, Table S1: Studies using biopolymers to bioengineer human liver from PSCs, Table S2: Studies using biopolymers to bioengineer human liver from LSCs, and Table S3: Studies using biopolymers to bioengineer human liver from NLSCs.

## Abbreviations

The following abbreviations are used in this manuscript:

2D	Two dimensional
3D	Three dimensional
AFP	Alpha-fetoprotein
CYP	Cytochrome P450
DED	Definitive Endoderm Differentiation
ECM	Extracellular Matrix
ESCs	Embryonic Stem Cells
GelMA	Gelatine Methacryloyl
GFR	Growth Factor Reduced
HA	Hyaluronic acid
HNF4	Hepatocyte Nuclear Factor-4
iPSCs	Induced Pluripotent Stem Cells
PIC-LEC	PIC-Laminin 111-Entactin-Complex
LSCs	Liver Stem Cells
LSECs	Liver Sinusoidal Endothelial Cells
MSCs	Mesenchymal Stem Cells
NLSCs	Non-Liver Stem Cells
PSCs	Pluripotent Stem Cells
PAA	Polyacrylamide
PEG	Polyethylene Glycol
PIC	Polyisocyanopeptides
PLLA/PCL	Poly-L-lactic acid/poly (*ε*-caprolactone)
PCL-Gel-HA	Poly *ε*-caprolactone-gelatine-hyaluronic acid
