# Supplementary material for: Biopolymers for Liver Tissue Engineering: A Systematic Review
Source: Gels. 2025 Jul 7;11(7):525. doi: 10.3390/gels11070525 (PMC12294766; doi:10.3390/gels11070525)
Supplement: Supplementary file 1 [file gels-11-00525-s001.zip › Table S1.pdf]

Table S1: Studies using biopolymers to bioengineer human liver from PSCs

S/N	PMID or DOI	Article	Stem cell type	End target	Maintenance Substrate	Differentiation substrate
1	39040044	Budi NYP, Lai WY, Huang YH, Ho HN. 3D organoid cultivation improves the maturation and functional differentiation of cholangiocytes from human pluripotent stem cells. Front Cell Dev Biol. 2024;12:1361084.	ipsc	Cholangiocytes	Matrigel	Matrigel
2	38664472	Weiand M, Sandfort V, Nadzemova O, Schierwagen R, Trebicka J, Schlevogt B, et al. Comparative analysis of SEC61A1 mutant R236C in two patient-derived cellular platforms. Sci Rep. 2024 Apr 25;14(1):9506.	ipsc	Cholangiocytes	Matrigel	Matrigel
3	34764255	Ogawa M, Jiang JX, Xia S, Yang D, Ding A, Laselva O, et al. Generation of functional ciliated cholangiocytes from human pluripotent stem cells. Nat Commun. 2021 Nov 11;12(1):6504.	ipsc	Cholangiocytes	Matrigel	Matrigel
4	33087715	Inada H, Udono M, Matsuda-Ito K, Horisawa K, Ohkawa Y, Miura S, et al. Direct reprogramming of human umbilical vein- and peripheral blood-derived endothelial cells into hepatic progenitor cells. Nat Commun. 2020 Oct 21;11(1):5292.	ipsc	Cholangiocytes	Matrigel	Matrigel
5	34519176	Jalan-Sakrikar N, De Assuncao TM, Navarro-Corcuera A, Hamdan FH, Loarca L, Kirkeby LA, et al. Induced Pluripotent Stem Cells From Subjects With Primary Sclerosing Cholangitis Develop a Senescence Phenotype Following Biliary Differentiation. Hepatol Commun. 2022 Feb;6(2):345–60.	ipsc	Cholangiocytes	Matrigel	Matrigel + Collagen-1
6	37995322	Deng S, Zhao X, Kou Z, Zhu Y, Zhang X, Chan HF. Effect of Valproic Acid on Promoting the Differentiation of Human Embryonic Stem Cells Into Cholangiocyte-Like Cells. Stem Cells Transl Med. 2024 Feb 14;13(2):166–76.	esc	Cholangiocytes	Matrigel (GFR)	Matrigel (GFR) + Collagen-1
7	35289126	Florentino RM, Li Q, Coard MC, Haep N, Motomura T, Diaz-Aragon R, et al. Transmembrane channel activity in human hepatocytes and cholangiocytes derived from induced pluripotent stem cells. Hepatol Commun. 2022 Jul;6(7):1561–73.	ipsc	Hepatocytes, cholangiocytes	Matrigel (GFR)	Matrigel (GFR) + Collagen-1
8	33243549	Poorna MR, Sudhindran S, Thampi MV, Mony U. Differentiation of induced pluripotent stem cells to hepatocyte-like cells on cellulose nanofibril substrate. Colloids Surf B Biointerfaces. 2021 Feb;198:111466.	ipsc	Hepatocytes	Matrigel	Cellulose
9	37357747	Marzec-Schmidt K, Ghosheh N, Stahlschmidt SR, Küppers-Munther B, Synnergren J, Ulfenborg B. Artificial Intelligence Supports Automated Characterization of Differentiated Human Pluripotent Stem Cells. Stem Cells. 2023 Sep 15;41(9):850–61.	ipsc	Hepatocytes	Cellartis DEF COAT-1	Cellartis Definitive Endoderm Differentiation Coating
10	35508708	Suzuki T, Furuhata E, Maeda S, Kishima M, Miyajima Y, Tanaka Y, et al. GATA6 is predicted to regulate DNA methylation in an in vitro model of human hepatocyte differentiation. Commun Biol. 2022 May 4;5(1):414.	ipsc	Hepatocytes	Cellartis DEF COAT-1	Cellartis Definitive Endoderm Differentiation Coating
11	36788249	Tanaka Y, Furuhata E, Maeda S, Kishima M, Suzuki H, Suzuki T. A dataset of definitive endoderm and hepatocyte differentiations from human induced pluripotent stem cells. Sci Data. 2023 Feb 14;10(1):93.	ipsc	Hepatocytes	Cellartis DEF COAT-1	Cellartis Definitive Endoderm Differentiation Coating
12	35898856	Gao X, Li R, Yourick JJ, Sprando RL. A transcriptomic dataset comparing two methods of hepatocyte differentiation from human induced pluripotent stem cells. Data Brief. 2022 Aug;43:108477.	ipsc	Hepatocytes	Cellartis DEF COAT-1	Cellartis Definitive Endoderm Differentiation Coating
13	35371477	Li R, Zhao Y, Yourick JJ, Sprando RL, Gao X. Phenotypical, functional and transcriptomic comparison of two modified methods of hepatocyte differentiation from human induced pluripotent stem cells. Biomed Rep. 2022 May;16(5):43.	ipsc	Hepatocytes	Cellartis DEF COAT-1	Cellartis Definitive Endoderm Differentiation Coating
14	39419394	Tsuchiya J, Miyoshi M, Kakinuma S, Kawai-Kitahata F, Kamiya A, Shimizu T, et al. Hepatitis B Virus-KMT2B Integration Drives Hepatic Oncogenic Processes in a Human Gene-edited Induced Pluripotent Stem Cells-derived Model. Cell Mol Gastroenterol Hepatol. 2025;19(2):101422.	ipsc	Hepatocytes	Cellartis DEF COAT-1	Cellartis Definitive Endoderm Differentiation Coating
15	35863491	Nell P, Kattler K, Feuerborn D, Hellwig B, Rieck A, Salhab A, et al. Identification of an FXR-modulated liver-intestine hybrid state in iPSC-derived hepatocyte-like cells. J Hepatol. 2022 Nov;77(5):1386–98.	ipsc	Hepatocytes	Cellartis DEF COAT-1	Cellartis Definitive Endoderm Differentiation Coating
16	32917265	Gao X, Li R, Cahan P, Zhao Y, Yourick JJ, Sprando RL. Hepatocyte-like cells derived from human induced pluripotent stem cells using small molecules: implications of a transcriptomic study. Stem Cell Res Ther. 2020 Sep 11;11(1):393.	ipsc	Hepatocytes	Cellartis DEF COAT-1	Cellartis DEF COAT-1
17	37566061	Fattahi P, de Hoyos-Vega JM, Choi JH, Duffy CD, Gonzalez-Suarez AM, Ishida Y, et al. Guiding Hepatic Differentiation of Pluripotent Stem Cells Using 3D Microfluidic Co-Cultures with Human Hepatocytes. Cells. 2023 Aug 1;12(15):1982.	ipsc	Hepatocytes	Feeder	Collagen-1
18	34059723	Tsuneishi R, Saku N, Miyata S, Akiyama S, Javaregowda PK, Ite K, et al. Ammonia-based enrichment and long-term propagation of zone I hepatocyte-like cells. Sci Rep. 2021 May 31;11(1):11381.	ipsc	Hepatocytes	Feeder	Collagen-1
19	**40050977**	Umezawa A, Fukuda A, Horikawa R, Uchida H, Enosawa S, Oishi Y, et al. First-in-human clinical study of an embryonic stem cell product for urea cycle disorders. Stem Cell Res Ther. 2025 Mar 6;16(1):120.	esc	Hepatocytes	Feeder	Collagen-1
20	37401657	Gong Y, Danoy M, Kido T, Mitsuhashi K, Choi H, Matsugi T, et al. Optimization of physical microenvironment to maintain the quiescence of human induced pluripotent stem cell-derived hepatic stellate cells. Biotechnol Bioeng. 2023 Aug;120(8):2345–56.	ipsc	Hepatocytes	Vitronectin	Collagen-1 vs. Matrigel
21	36640555	Blackford SJI, Yu TTL, Norman MDA, Syanda AM, Manolakakis M, Lachowski D, et al. RGD density along with substrate stiffness regulate hPSC hepatocyte functionality through YAP signalling. Biomaterials. 2023 Feb;293:121982.	ipsc	Hepatocytes	Vitronectin	Collagen-1 vs. PEG-Peptides
22	36766839	Liu S, Wang J, Chen S, Han Z, Wu H, Chen H, et al. C/EBPβ Coupled with E2F2 Promoted the Proliferation of hESC-Derived Hepatocytes through Direct Binding to the Promoter Regions of Cell-Cycle-Related Genes. Cells. 2023 Feb 2;12(3):497.	ipsc	Hepatocytes	Feeder	Feeder
23	37678970	Akiyama S, Saku N, Miyata S, Ite K, Nonaka H, Toyoda M, et al. Drug metabolic activity as a selection factor for pluripotent stem cell-derived hepatic progenitor cells. Prog Mol Biol Transl Sci. 2023;199:155–78.	ipsc	Hepatocytes	Feeder	Feeder
24	31989141	Danoy M, Poulain S, Koui Y, Tauran Y, Scheidecker B, Kido T, et al. Transcriptome profiling of hiPSC-derived LSECs with nanoCAGE. Mol Omics. 2020 Apr 1;16(2):138–46.	ipsc	LSEC	Feeder	Feeder
25	34229994	Danoy M, Jellali R, Tauran Y, Bruce J, Leduc M, Gilard F, et al. Characterization of the proteome and metabolome of human liver sinusoidal endothelial-like cells derived from induced pluripotent stem cells. Differentiation. 2021;120:28–35.	ipsc	Hepatocytes	Feeder	Fibronectin
26	35388883	Song D, Takahashi G, Zheng YW, Matsuo-Takasaki M, Li J, Takami M, et al. Retinoids rescue ceruloplasmin secretion and alleviate oxidative stress in Wilson's disease-specific hepatocytes. Hum Mol Genet. 2022 Oct 28;31(21):3652–71.	ipsc	Hepatocytes	Laminin 511-Silk	Gelatin
27	36980206	Hussein M, Pasqua M, Pereira U, Benzoubir N, Duclos-Vallée JC, Dubart-Kupperschmitt A, et al. Microencapsulated Hepatocytes Differentiated from Human Induced Pluripotent Stem Cells: Optimizing 3D Culture for Tissue Engineering Applications. Cells. 2023 Mar 10;12(6):865.	ipsc	Hepatocytes	Matrigel (GFR)	Gelatin
28	34687060	Luce E, Steichen C, Allouche M, Messina A, Heslan JM, Lambert T, et al. In vitro recovery of FIX clotting activity as a marker of highly functional hepatocytes in a hemophilia B iPSC model. Hepatology. 2022 Apr;75(4):866–80.	ipsc	Hepatocytes	Matrigel (GFR)	Gelatin
29	35338220	Imamura S, Yoshimoto K, Terada S, Takamuro K, Kamei KI. In vitro culture at 39 °C during hepatic maturation of human ES cells facilitates hepatocyte-like cell functions. Sci Rep. 2022 Mar 25;12(1):5155.	esc	Hepatocytes	Matrigel	Gelatin
30	38954820	Kent D, Ng SS, Syanda AM, Khoshkenar P, Ronzoni R, Li CZ, et al. Reduction of Z alpha-1 antitrypsin polymers in human iPSC-hepatocytes and mice by LRRK2 inhibitors. Hepatology. 2025 Mar 1;81(3):903–16.	ipsc	Hepatocytes	Vitronectin	Gelatin
31	38909044	Ng NHJ, Ghosh S, Bok CM, Ching C, Low BSJ, Chen JT, et al. HNF4A and HNF1A exhibit tissue specific target gene regulation in pancreatic beta cells and hepatocytes. Nat Commun. 2024 Jun 22;15(1):4288.	ipsc	Hepatocytes	Vitronectin	Gelatin
32	37484212	Grove JI, Lo PCK, Shrine N, Barwell J, Wain LV, Tobin MD, et al. Identification and characterisation of a rare MTTP variant underlying hereditary non-alcoholic fatty liver disease. JHEP Rep. 2023 Aug;5(8):100764.	ipsc	Hepatocytes	Vitronectin	Gelatin

33	35284810	Li CZ, Ogawa H, Ng SS, Chen X, Kishimoto E, Sakabe K, et al. Human iPSC-derived hepatocyte system models cholestasis with tight junction protein 2 deficiency. JHEP Rep. 2022 Apr;4(4):100446.	ipsc	Hepatocytes	Vitronectin	Gelatin
34	32304089	Kong FE, Tang YQ, Gong YF, Mo JQ, Zhao Y, Li MM, et al. Identification of prognostic claudins signature in hepatocellular carcinoma from a hepatocyte differentiation model. Hepatol Int. 2020 Jul;14(4):521–33.	esc	Hepatocytes	Feeder	Gelatin
35	38915486	Zimmerlin L, Angarita A, Park TS, Evans-Moses R, Thomas J, Yan S, et al. Proteogenomic Reprogramming to a Functional Human Totipotent Stem Cell State via a PARP-DUX4 Regulatory Axis. bioRxiv. 2024 Jun 15;2024.06.14.598510.	esc	Hepatocytes	Vitronectin	Gelatin
36	34861166	Koui Y, Himeno M, Mori Y, Nakano Y, Saijou E, Tanimizu N, et al. Development of human iPSC-derived quiescent hepatic stellate cell-like cells for drug discovery and in vitro disease modeling. Stem Cell Reports. 2021 Dec 14;16(12):3050–63.	ipsc	Hepatic Stellate Cells	Vitronectin	Gelatin
37	39927149	Ashmore-Harris C, Ayabe H, Yoshizawa E, Arisawa T, Takada Y, Takebe T, et al. Gene editing enables non-invasive in vivo PET imaging of human induced pluripotent stem cell-derived liver bud organoids. Mol Ther Methods Clin Dev. 2025 Mar 13;33(1):101406.	ipsc	Hepatocytes, Liver organoids	Vitronectin	Gelatin
38	34304139	Kumar M, Toprakhisar B, Van Haele M, Antoranz A, Boon R, Chesnais F, et al. A fully defined matrix to support a pluripotent stem cell derived multi-cell-liver steatohepatitis and fibrosis model. Biomaterials. 2021 Sep;276:121006.	esc	Hepatocytes, Liver sinusoidal endothelial cells	Matrigel	HepMat
39	40070824	Gantier M, Ménoret S, Fourrier A, Delbos F, Nguyen TH, Anegon I. Human pluripotent stem cell-derived hepatic progenitors exhibit a partially hypoimmunogenic phenotype and actively inhibit immune responses. Front Immunol. 2025;16:1507317.	ipsc	Hepatocytes	Vitronectin	Laminin 511
40	38956724	Tsuneyoshi N, Hosoya T, Takeno Y, Saitoh K, Murai H, Amimoto N, et al. Hypoimmunogenic human iPSCs expressing HLA-G, PD-L1, and PD-L2 evade innate and adaptive immunity. Stem Cell Res Ther. 2024 Jul 2;15(1):193.	ipsc	Hepatocytes	Laminin 511	Laminin 511
41	38475825	Gantier M, Rispal R, Fourrier A, Ménoret S, Delbos F, Anegon I, et al. Cryopreserved cGMP-compliant human pluripotent stem cell-derived hepatic progenitors rescue mice from acute liver failure through rapid paracrine effects on liver cells. Stem Cell Res Ther. 2024 Mar 12;15(1):71.	ipsc	Hepatocytes	Vitronectin	Laminin 511
42	32303458	Negoro R, Kawai K, Ichikawa M, Deguchi S, Takayama K, Mizuguchi H. Establishment of MDR1-knockout human induced pluripotent stem cell line. Drug Metab Pharmacokinet. 2020 Jun;35(3):288–96.	ipsc	Hepatocytes	Laminin 511	Laminin 511
43	38879647	Tian SP, Ge JY, Song YM, Yu XQ, Chen WH, Chen YY, et al. A novel efficient strategy to generate liver sinusoidal endothelial cells from human pluripotent stem cells. Sci Rep. 2024 Jun 15;14(1):13831.	ipsc	Hepatocytes	Laminin 511	Laminin 511
44	37927418	Fujisaka Y, Nakagawa T, Tomoda K, Watanabe M, Matsunaga N, Tamura Y, et al. The cytotoxicity of gefitinib on patient-derived induced pluripotent stem cells reflects gefitinib-induced liver injury in the clinical setting. Oncol Lett. 2023 Dec;26(6):520.	ipsc	Hepatocytes	Laminin 511	Laminin 511-Silk
45	37108241	Tsuzuki S, Yamaguchi T, Okumura T, Kasai T, Ueno Y, Taniguchi H. PDGF Receptors and Signaling Are Required for 3D-Structure Formation and Differentiation of Human iPSC-Derived Hepatic Spheroids. Int J Mol Sci. 2023 Apr 11;24(8):7075.	ipsc	Hepatocytes	Laminin 511	Laminin 511-Silk
46	36524054	Bando K, Yamashita H, Tsumori M, Minoura H, Okumura K, Hattori F. Compact automated culture machine for human induced pluripotent stem cell maintenance and differentiation. Front Bioeng Biotechnol. 2022;10:1074990.	ipsc	Hepatocytes	Laminin 511-Silk	Laminin 511-Silk
47	38872960	Okano M, Yasuda M, Shimomura Y, Matsuoka Y, Shirouzu Y, Fujioka T, et al. Citrin-deficient patient-derived induced pluripotent stem cells as a pathological liver model for congenital urea cycle disorders. Mol Genet Metab Rep. 2024 Sep;40:101096.	ipsc	Hepatocytes	Laminin 511-Silk	Laminin 511-Silk
48	35354033	Krumm J, Sekine K, Samaras P, Brazovskaja A, Breunig M, Yasui R, et al. High temporal resolution proteome and phosphoproteome profiling of stem cell-derived hepatocyte development. Cell Rep. 2022 Mar 29;38(13):110604.	ipsc	Hepatocytes	Laminin 511	Laminin 511
49	33087763	Sekine K, Ogawa S, Tsuzuki S, Kobayashi T, Ikeda K, Nakanishi N, et al. Generation of human induced pluripotent stem cell-derived liver buds with chemically defined and animal origin-free media. Sci Rep. 2020 Oct 21;10(1):17937.	ipsc	Hepatocytes, Liver organoids	Laminin 511	Laminin 511, then Matrigel
50	34861164	Tristan CA, Ormanoglu P, Slamecka J, Malley C, Chu PH, Jovanovic VM, et al. Robotic high-throughput biomanufacturing and functional differentiation of human pluripotent stem cells. Stem Cell Reports. 2021 Dec 14;16(12):3076–92.	ipsc	Hepatocytes	Vitronectin	Laminin 521
51	36291161	Koenig L, Ramme AP, Faust D, Mayer M, Flötke T, Gerhartl A, et al. A Human Stem Cell-Derived Brain-Liver Chip for Assessing Blood-Brain-Barrier Permeation of Pharmaceutical Drugs. Cells. 2022 Oct 19;11(20):3295.	ipsc	Hepatocytes	Matrigel (GFR)	Laminin 521
52	35569737	Briso-Montiano Á, Vilas A, Richard E, Ruiz-Sala P, Morato E, Desviat LR, et al. Hepatocyte-like cells differentiated from methylmalonic aciduria cblB type induced pluripotent stem cells: A platform for the evaluation of pharmacochaperoning. Biochim Biophys Acta Mol Basis Dis. 2022 Sep 1;1868(9):166433.	ipsc	Hepatocytes	Laminin 521	Laminin 521
53	33007774	Lucendo-Villarin B, Meseguer-Ripolles J, Drew J, Fischer L, Ma E, Flint O, et al. Development of a cost-effective automated platform to produce human liver spheroids for basic and applied research. Biofabrication. 2020 Oct 28;13(1).	ipsc	Hepatocytes	Laminin 521	Laminin 521
54	33377458	Wu D, Chen X, Sheng Q, Chen W, Zhang Y, Wu F. Production of Functional Hepatobiliary Organoids from Human Pluripotent Stem Cells. Int J Stem Cells. 2021 Feb 28;14(1):119–26.	ipsc	Hepatocytes	Laminin 521	Laminin 521
55	33556073	Leedale JA, Lucendo-Villarin B, Meseguer-Ripolles J, Kasarinaite A, Webb SD, Hay DC. Mathematical modelling of oxygen gradients in stem cell-derived liver tissue. PLoS One. 2021;16(2):e0244070.	ipsc	Hepatocytes	Laminin 521	Laminin 521
56	40133415	Nirgude S, Tichy ED, Liu Z, Kavari SL, Pradieu RD, Byrne M, et al. Single-nucleus multiomic analysis of Beckwith-Wiedemann syndrome liver reveals PPARA signaling enrichment and metabolic dysfunction. Commun Biol. 2025 Mar 26;8(1):495.	ipsc	Hepatocytes	Matrigel (GFR)	Laminin 521
57	39890799	Smiriglia A, Lorito N, Bacci M, Subbiani A, Bonechi F, Comito G, et al. Estrogen-dependent activation of TRX2 reverses oxidative stress and metabolic dysfunction associated with steatotic disease. Cell Death Dis. 2025 Jan 31;16(1):57.	esc	Hepatocytes	Laminin 521	Laminin 521
58	39598796	Collins JM, Wang D. DNA Methylation in the CYP3A Distal Regulatory Region (DRR) Is Associated with the Expression of CYP3A5 and CYP3A7 in Human Liver Samples. Molecules. 2024 Nov 16;29(22):5407.	ipsc	Hepatocytes	Laminin 521	Laminin 521
59	34200130	Bogacheva MS, Bystriakova MA, Lou YR. Thyroid Hormone Effect on the Differentiation of Human Induced Pluripotent Stem Cells into Hepatocyte-Like Cells. Pharmaceuticals (Basel). 2021 Jun 7;14(6):544.	ipsc	Hepatocytes	Laminin 521	Laminin 521
60	32481600	Fischer L, Lucendo-Villarin B, Hay DC, O'Farrelly C. Human PSC-Derived Hepatocytes Express Low Levels of Viral Pathogen Recognition Receptors, but Are Capable of Mounting an Effective Innate Immune Response. Int J Mol Sci. 2020 May 28;21(11):3831.	msc	Hepatocytes	Laminin 521	Laminin 521
61	40069876	Kasarinaite A, Ramos MJ, Beltran-Sierra M, Sutherland EF, Rei PA, Zhao M, et al. Hormone correction of dysfunctional metabolic gene expression in stem cell-derived liver tissue. Stem Cell Res Ther. 2025 Mar 11;16(1):130.	ipsc	Hepatocytes	Laminin 521	Laminin 521
62	39572522	Krivec N, Couvreu de Deckersberg E, Lei Y, Al Delbany D, Regin M, Verhulst S, et al. Gain of 1q confers an MDM4-driven growth advantage to undifferentiated and differentiating hESC while altering their differentiation capacity. Cell Death Dis. 2024 Nov 21;15(11):852.	ipsc	Hepatocytes	Laminin 521	Laminin 521
63	33409477	Sinton MC, Meseguer-Ripolles J, Lucendo-Villarin B, Wernig-Zorc S, Thomson JP, Carter RN, et al. A human pluripotent stem cell model for the analysis of metabolic dysfunction in hepatic steatosis. iScience. 2021 Jan 22;24(1):101931.	ipsc	Hepatocytes	Laminin 521	Laminin 521
64	37366409	Pridgeon CS, Forootan SS, Zhang F, Harper N, Palmer D, Weightmann R, et al. In Vivo Tumorigenicity of the 20q11.21 Amplicon in an Engraftment Model of hPSCs and Differentiated Liver Cells. J Stem Cells Regen Med. 2023;19(1):3–13.	esc	Hepatocytes	Laminin 521	Laminin 521
65	10.1242/bio.054189	Graffmann N, Ncube A, Martins S, Fiszl AR, Reuther P, Bohndorf M, et al. A stem cell based in vitro model of NAFLD enables the analysis of patient specific individual metabolic adaptations in response to a high fat diet and AdipoRon interference. Biology Open. 2021 Jan 25;10(1):bio054189.	ipsc	Hepatocytes	Laminin 521	Laminin 521 + Laminin 111

66	35120625	Raggi C, M'Callum MA, Pham QT, Gaub P, Selleri S, Baratang NV, et al. Leveraging interacting signaling pathways to robustly improve the quality and yield of human pluripotent stem cell-derived hepatoblasts and hepatocytes. Stem Cell Reports. 2022 Mar 8;17(3):584–98.	ipsc	Hepatocytes, Liver organoids	Laminin 521	Laminin 521 then suspension culture
67	35435152	Antarianto RD, Pragiwaksana A, Septiana WL, Mazfufah NF, Mahmood A. Hepatocyte Differentiation from iPSCs or MSCs in Decellularized Liver Scaffold: Cell-ECM Adhesion, Spatial Distribution, and Hepatocyte Maturation Profile. Organogenesis. 2022 Dec 31;18(1):2061263.	ipsc	Hepatocytes	Vitronectin	Rabbit liver scaffold, ECM
68	32492423	Takeishi K, Collin de l'Hortet A, Wang Y, Handa K, Guzman-Lepe J, Matsubara K, et al. Assembly and Function of a Bioengineered Human Liver for Transplantation Generated Solely from Induced Pluripotent Stem Cells. Cell Rep. 2020 Jun 2;31(9):107711.	ipsc	Hepatocytes	Matrigel	Matrigel (GFR)
69	36515690	Warren I, Moeller MM, Guiggey D, Chiang A, Maloy M, Ogoke O, et al. FOXA1/2 depletion drives global reprogramming of differentiation state and metabolism in a human liver cell line and inhibits differentiation of human stem cell-derived hepatic progenitor cells. FASEB J. 2023 Jan;37(1):e22652.	esc	Hepatocytes	Matrigel (GFR)	Matrigel (GFR)
70	36717592	Alexanova A, Raitoharju E, Valtonen J, Aalto-Setälä K, Viiri LE. Coronary artery disease patient-derived iPSC-hepatocytes have distinct miRNA profile that may alter lipid metabolism. Sci Rep. 2023 Jan 30;13(1):1706.	ipsc	Hepatocytes	Matrigel (GFR)	Matrigel (GFR)
71	37400454	Groeger M, Matsuo K, Heidary Arash E, Pereira A, Le Guillou D, Pino C, et al. Modeling and therapeutic targeting of inflammation-induced hepatic insulin resistance using human iPSC-derived hepatocytes and macrophages. Nat Commun. 2023 Jul 3;14(1):3902.	ipsc	Hepatocytes	Matrigel (GFR)	Matrigel (GFR)
72	37734628	Wesseler MF, Taebnia N, Harrison S, Youhanna S, Preiss LC, Kemas AM, et al. 3D microperfusion of mesoscale human microphysiological liver models improves functionality and recapitulates hepatic zonation. Acta Biomater. 2023 Nov;171:336–49.	esc, ipsc	Hepatocytes	Matrigel (GFR)	Matrigel (GFR)
73	37830581	Suominen S, Hyypijev T, Venäläinen M, Yrjänäinen A, Vuorenpää H, Lehti-Polojärvi M, et al. Improvements in Maturity and Stability of 3D iPSC-Derived Hepatocyte-like Cell Cultures. Cells. 2023 Sep 27;12(19):2368.	ipsc	Hepatocytes	Feeder	Matrigel (GFR)
74	37903763	Tschuck J, Theilacker L, Rothenaigner I, Weiß SAI, Akdogan B, Lam VT, et al. Farnesoid X receptor activation by bile acids suppresses lipid peroxidation and ferroptosis. Nat Commun. 2023 Oct 30;14(1):6908.	ipsc	Hepatocytes	Matrigel (GFR)	Matrigel (GFR)
75	38026193	Ori C, Ansari M, Angelidis I, Olmer R, Martin U, Theis FJ, et al. Human pluripotent stem cell fate trajectories toward lung and hepatocyte progenitors. iScience. 2023 Nov 17;26(11):108205.	ipsc	Hepatocytes	Matrigel (GFR)	Matrigel (GFR)
76	34288010	Tilson SG, Morell CM, Lenaerts AS, Park SB, Hu Z, Jenkins B, et al. Modeling PNPLA3-Associated NAFLD Using Human-Induced Pluripotent Stem Cells. Hepatology. 2021 Dec;74(6):2998–3017.	ipsc	Hepatocytes	Vitronectin	Matrigel (GFR)
77	34786702	Laemmle A, Poms M, Hsu B, Borsuk M, Rüfenacht V, Robinson J, et al. Aquaporin 9 induction in human iPSC-derived hepatocytes facilitates modeling of ornithine transcarbamylase deficiency. Hepatology. 2022 Sep;76(3):646–59.	ipsc	Hepatocytes	Feeder	Matrigel (GFR)
78	35240497	O G, Cascione S, Michielin F, Elvassore N. The emergence of the circadian clock network in hiPSC-derived hepatocytes on chip. Biochem Biophys Res Commun. 2022 Apr 23;601:109–15.	ipsc	Hepatocytes	Matrigel (GFR)	Matrigel (GFR)
79	35385743	Gage BK, Merlin S, Olgasi C, Follenzi A, Keller GM. Therapeutic correction of hemophilia A by transplantation of hPSC-derived liver sinusoidal endothelial cell progenitors. Cell Rep. 2022 Apr 5;39(1):110621.	ipsc	Hepatocytes	Gelatin + Matrigel	Matrigel (GFR)
80	39453535	Foglia M, Guarrera L, Kurosaki M, Cassanmagnago GA, Bolis M, Miduri M, et al. The NIPBL-gene mutation of a Cornelia de Lange Syndrome patient causes deficits in the hepatocyte differentiation of induced Pluripotent Stem Cells via altered chromatin-accessibility. Cell Mol Life Sci. 2024 Oct 25;81(1):439.	ipsc	Hepatocytes	Matrigel (GFR)	Matrigel (GFR)
81	38951656	Westerberg NS, Atneosen-Åsegg M, Melheim M, Chollet ME, Harrison SP, Siller R, et al. Effect of hypoxia on aquaporins and hepatobiliary transport systems in human hepatic cells. Pediatr Res. 2025 Jan;97(1):195–201.	ipsc	Hepatocytes	Matrigel (GFR)	Matrigel (GFR)
82	38818583	Feng Z, Zhou B, Shuai Q, Wei Y, Jin N, Wang X, et al. Development of an alcoholic liver disease model for drug evaluation from human induced pluripotent stem cell-derived liver organoids. Acta Biochim Biophys Sin (Shanghai). 2024 May 30;56(10):1460–72.	ipsc	Hepatocytes	Matrigel (GFR)	Matrigel (GFR)
83	38425868	Mathis D, Koch J, Koller S, Sauter K, Flück C, Uldry AC, et al. Induced pluripotent stem cell-derived hepatocytes reveal TCA cycle disruption and the potential basis for triheptanoin treatment for malate dehydrogenase 2 deficiency. Mol Genet Metab Rep. 2024 Jun;39:101066.	ipsc	Hepatocytes	Matrigel (GFR)	Matrigel (GFR)
84	38053928	Ramosaj A, Singhal P, Schaller A, Laemmle A. Induced pluripotent stem cell technology as diagnostic tool in patients with suspected ornithine transcarbamylase deficiency lacking genetic confirmation. Mol Genet Metab Rep. 2023 Dec;37:101007.	ipsc	Hepatocytes	Matrigel (GFR)	Matrigel (GFR)
85	36439639	Edwards JS, Delabat SA, Badilla AD, DiCaprio RC, Hyun J, Burgess RA, et al. Downregulation of SOCS1 increases interferon-induced ISGylation during differentiation of induced-pluripotent stem cells to hepatocytes. JHEP Rep. 2022 Dec;4(12):100592.	ipsc	Hepatocytes	Matrigel (GFR)	Matrigel (GFR)
86	36147945	Nakashima Y, Miyagi-Shiohira C, Saitoh I, Watanabe M, Matsushita M, Tsukahara M, et al. Induced hepatic stem cells are suitable for human hepatocyte production. iScience. 2022 Oct 21;25(10):105052.	ipsc	Hepatocytes	Gelatin	Matrigel (GFR)
87	35959725	Tomaz RA, Zacharis ED, Bachinger F, Wurmser A, Yamamoto D, Petrus-Reurer S, et al. Generation of functional hepatocytes by forward programming with nuclear receptors. Elife. 2022 Aug 12;11:e71591.	ipsc	Hepatocytes	Vitronectin	Matrigel (GFR)
88	35051043	Ghosh S, De Smedt J, Tricot T, Proença S, Kumar M, Nami F, et al. HiPSC-Derived Hepatocyte-like Cells Can Be Used as a Model for Transcriptomics-Based Study of Chemical Toxicity. Toxics. 2021 Dec 21;10(1):1.	ipsc	Hepatocytes	Matrigel	Matrigel (GFR)
89	33133779	Kulkeaw K, Tubsuwan A, Tongkrajang N, Whangviboonkij N. Generation of human liver organoids from pluripotent stem cell-derived hepatic endoderms. PeerJ. 2020;8:e9968.	ipsc	Hepatocytes	Matrigel (GFR)	Matrigel (GFR)
90	39044210	Wilhelmsen I, Combriat T, Dalmao-Fernandez A, Stokowiec J, Wang C, Olsen PA, et al. The effects of TGF-β-induced activation and starvation of vitamin A and palmitic acid on human stem cell-derived hepatic stellate cells. Stem Cell Res Ther. 2024 Jul 23;15(1):223.	ESC	Hepatocytes	Matrigel (GFR)	Matrigel (GFR)
91	35621497	Acun A, Oganesyan R, Jaramillo M, Yarmush ML, Uygun BE. Human-Origin iPSC-Based Recellularization of Decellularized Whole Rat Livers. Bioengineering (Basel). 2022 May 19;9(5):219.	ipsc	Hepatocytes	Matrigel (GFR)	Matrigel (GFR)
92	34430783	Deguchi S, Shintani T, Harada K, Okamoto T, Takemura A, Hirata K, et al. In Vitro Model for a Drug Assessment of Cytochrome P450 Family 3 Subfamily A Member 4 Substrates Using Human Induced Pluripotent Stem Cells and Genome Editing Technology. Hepatol Commun. 2021 Aug;5(8):1385–99.	ipsc	Hepatocytes	Feeder	Matrigel (GFR)
93	34025426	Lee-Montiel FT, Laemmle A, Charwat V, Dumont L, Lee CS, Huebsch N, et al. Integrated Isogenic Human Induced Pluripotent Stem Cell-Based Liver and Heart Microphysiological Systems Predict Unsafe Drug-Drug Interaction. Front Pharmacol. 2021;12:667010.	ipsc	Hepatocytes	Matrigel (GFR)	Matrigel (GFR)
94	33640436	Duwaerts CC, Le Guillou D, Her CL, Phillips NJ, Willenbring H, Mattis AN, et al. Induced Pluripotent Stem Cell-derived Hepatocytes From Patients With Nonalcoholic Fatty Liver Disease Display a Disease-specific Gene Expression Profile. Gastroenterology. 2021 Jun;160(7):2591-2594.e6.	ipsc	Hepatocytes	Matrigel (GFR)	Matrigel (GFR)
95	33305175	Lorvellec M, Pellegata AF, Maestri A, Turchetta C, Alvarez Mediavilla E, Shibuya S, et al. An In Vitro Whole-Organ Liver Engineering for Testing of Genetic Therapies. iScience. 2020 Dec 18;23(12):101808.	ipsc	Hepatocytes	Matrigel (GFR)	Matrigel (GFR)
96	39995235	Kogler S, Skottvoll FYS, Hrušková H, Rise F, Aizenshtadt A, Krauss S, et al. Electromembrane Extraction Provides Unprecedented Selectivity for Drugs in Cell Culture Media Used in Organoid and Organ-on-Chip Systems. Anal Chem. 2025 Mar 11;97(9):4923–31.	ipsc	Hepatocytes	Matrigel (GFR)	Matrigel (GFR)
97	38266310	Acun A, Fan L, Oganesyan R, Uygun KM, Yeh H, Yarmush ML, et al. Effect of Donor Age and Liver Steatosis on Potential of Decellularized Liver Matrices to be used as a Platform for iPSC-Hepatocyte Culture. Adv Healthc Mater. 2024 May;13(13):e2302943.	ipsc	Hepatocytes	Matrigel (GFR)	Matrigel (GFR) vs. Decellularised Human Liver ECM
98	35006958	Enomoto J, Toba Y, Yamazaki H, Kanai M, Mizuguchi H, Matsui H. Development of a 3D Cell Culture System Using Amphiphilic Polydepsipeptides and Its Application to Hepatic Differentiation. ACS Appl Bio Mater. 2021 Sep 20;4(9):7290–9.	ipsc	Hepatocytes, Liver organoids	Laminin 511	Hydrox vs. Matrigel
99	32516515	Munroe M, Niero EL, Fok WC, Vessoni AT, Jeong HC, Brenner KA, et al. Telomere Dysfunction Activates p53 and Represses HNF4α Expression Leading to Impaired Human Hepatocyte Development and Function. Hepatology. 2020 Oct;72(4):1412–29.	ipsc	Hepatocytes	Matrigel	Matrigel

100	32746905	Choi JS, Jeong IS, Park YJ, Kim SW. HGF and IL-10 expressing ALB::GFP reporter cells generated from iPSCs show robust anti-fibrotic property in acute fibrotic liver model. Stem Cell Res Ther. 2020 Aug 3;11(1):332.	ipsc	Hepatocytes	Vitronectin	Matrigel
101	33393184	Lu Y, Dai W, Huang J, Chen X, Yao Y. A Biomimetic Glue Protein Modulates Hepatic Gene Expression. Macromol Biosci. 2021 Mar;21(3):e2000303.	ipsc	Hepatocytes	Laminin 511	Matrigel
102	34982937	Moreno-Torres M, Kumar M, García-Llorens G, Quintás G, Tricot T, Boon R, et al. A Novel UPLC-MS Metabolomic Analysis-Based Strategy to Monitor the Course and Extent of iPSC Differentiation to Hepatocytes. J Proteome Res. 2022 Mar 4;21(3):702–12.	ipsc	Hepatocytes	Matrigel	Matrigel
103	10.1242/bio.055087	Gurevich I, Burton SA, Munn C, Ohshima M, Goedland ME, Czysz K, et al. iPSC-derived hepatocytes generated from NASH donors provide a valuable platform for disease modeling and drug discovery. Biol Open. 2020 Dec 16;9(12):bio055087.	ipsc	Hepatocytes	Matrigel	Matrigel
104	10.1016/j.apmt.2020.100730	Zheng L, Lü D, Zhang F, Xing M, Wang X, Jia X, et al. Regulation of hepatic differentiation of human embryonic stem cells by calcium silicate extracts for liver injury repairing. Applied Materials Today. 2020 Sep 1;20:100730.	esc	Hepatocytes	Matrigel	Matrigel
105	38579669	Jalil S, Keskinen T, Juutila J, Sartori Maldonado R, Euro L, Suomalainen A, et al. Genetic and functional correction of argininosuccinate lyase deficiency using CRISPR adenine base editors. Am J Hum Genet. 2024 Apr 4;111(4):714–28.	ipsc	Hepatocytes	Matrigel	Matrigel
106	39146183	Kent GM, Atkins MH, Lung B, Nikitina A, Fernandes IM, Kwan JJ, et al. Human liver sinusoidal endothelial cells support the development of functional human pluripotent stem cell-derived Kupffer cells. Cell Rep. 2024 Aug 27;43(8):114629.	ipsc	Hepatocytes	Feeder	Matrigel
107	36049612	Park J, Zhao Y, Zhang F, Zhang S, Kwong AC, Zhang Y, et al. IL-6/STAT3 axis dictates the PNPLA3-mediated susceptibility to non-alcoholic fatty liver disease. J Hepatol. 2023 Jan;78(1):45–56.	ipsc, esc	Hepatocytes	Matrigel (GFR)	Matrigel
108	36076262	Bai F, Duan J, Yang D, Lai X, Zhu X, He X, et al. Integrative network analysis of circular RNAs reveals regulatory mechanisms for hepatic specification of human iPSC-derived endoderm. Stem Cell Res Ther. 2022 Sep 8;13(1):468.	ipsc	Hepatocytes	Vitronectin	Matrigel
109	36231094	Xie X, Zhou X, Liu T, Zhong Z, Zhou Q, Iqbal W, et al. Direct Differentiation of Human Embryonic Stem Cells to 3D Functional Hepatocyte-like Cells in Alginate Microencapsulation Sphere. Cells. 2022 Oct 5;11(19):3134.	esc	Hepatocytes	Matrigel	Matrigel
110	36270282	Lai X, Li C, Xiang C, Pan Z, Zhang K, Wang L, et al. Generation of functionally competent hepatic stellate cells from human stem cells to model liver fibrosis in vitro. Stem Cell Reports. 2022 Nov 8;17(11):2531–47.	ipsc, esc	Hepatocytes	Matrigel (GFR)	Matrigel
111	36476855	Kaserman JE, Werder RB, Wang F, Matte T, Higgins MI, Dodge M, et al. Human iPSC-hepatocyte modeling of alpha-1 antitrypsin heterozygosity reveals metabolic dysregulation and cellular heterogeneity. Cell Rep. 2022 Dec 6;41(10):111775.	ipsc	Hepatocytes	Matrigel (GFR)	Matrigel
112	36552880	Wang J, Situ P, Chen S, Wu H, Zhang X, Liu S, et al. Hepatic Polarized Differentiation Promoted the Maturity and Liver Function of Human Embryonic Stem Cell-Derived Hepatocytes via Activating Hippo and AMPK Signaling Pathways. Cells. 2022 Dec 18;11(24):4117.	esc	Hepatocytes	Feeder	Matrigel
113	37165871	Gawronski KAB, Bone WP, Park Y, Pashos EE, Wenz BM, Dudek MF, et al. Evaluating the Contribution of Cell Type-Specific Alternative Splicing to Variation in Lipid Levels. Circ Genom Precis Med. 2023 Jun;16(3):248–57.	ipsc	Hepatocytes	Feeder	Matrigel
114	10.1088/2053-158	Kang HK, Kim DJ, Kim MS, Kim DH, Lee JY, Sung EA, et al. Improved hepatoblast differentiation of human pluripotent stem cells by coffee bean derived graphene quantum dots. 2D Mater. 2022 May;9(3):035012.	ipsc, esc	Hepatocytes	Feeder	Matrigel
115	37293770	Li M, Ueyama-Toba Y, Lindley M, Kongklad G, Nawa Y, Kumamoto Y, et al. Label-Free Evaluation of Maturation and Hepatotoxicity of Human iPSC-Derived Hepatocytes Using Hyperspectral Raman Imaging. Anal Chem. 2023 Jun 20;95(24):9252–62.	ipsc	Hepatocytes	Laminin 511	Matrigel
116	37302654	Choi YJ, Kim MS, Rhoades JH, Johnson NM, Berry CT, Root S, et al. Patient-Induced Pluripotent Stem Cell-Derived Hepatostellate Organoids Establish a Basis for Liver Pathologies in Telomeropathies. Cell Mol Gastroenterol Hepatol. 2023;16(3):451–72.	ipsc	Hepatocytes	Vitronectin	Matrigel
117	38006857	Zhang S, Yang R, Zhao M, Li S, Yin N, Zhang A, et al. Typical neonicotinoids and organophosphate esters, but not their metabolites, adversely impact early human development by activating BMP4 signaling. J Hazard Mater. 2024 Mar 5;465:133028.	ipsc	Hepatocytes	Vitronectin	Matrigel
118	34934826	Morita A, Omoya Y, Ito R, Ishibashi Y, Hiramoto K, Ohnishi S, et al. Glycyrrhizin and its derivatives promote hepatic differentiation via sweet receptor, Wnt, and Notch signaling. Biochem Biophys Rep. 2021 Dec;28:101181.	ipsc	Hepatocytes	Feeder	Matrigel
119	35410439	Pan T, Wang N, Zhang J, Yang F, Chen Y, Zhuang Y, et al. Efficiently generate functional hepatic cells from human pluripotent stem cells by complete small-molecule strategy. Stem Cell Res Ther. 2022 Apr 11;13(1):159.	esc, ipsc	Hepatocytes	Matrigel	Matrigel
120	35452598	Ma H, de Zwaan E, Guo YE, Cejas P, Thiru P, van de Bunt M, et al. The nuclear receptor THRB facilitates differentiation of human PSCs into more mature hepatocytes. Cell Stem Cell. 2022 May 5;29(5):795-809.e11.	ipsc	Hepatocytes	Matrigel	Matrigel
121	35455938	Kojima H, Yagi H, Kushige H, Toda Y, Takayama K, Masuda S, et al. Decellularized Organ-Derived Scaffold Is a Promising Carrier for Human Induced Pluripotent Stem Cells-Derived Hepatocytes. Cells. 2022 Apr 7;11(8):1258.	ipsc	Hepatocytes	Laminin 511	Matrigel
122	35915082	Varghese DS, Alawathugoda TT, Sheikh MA, Challagandla AK, Emerald BS, Ansari SA. Developmental modeling of hepatogenesis using obese iPSCs-hepatocyte differentiation uncovers pathological features. Cell Death Dis. 2022 Aug 1;13(8):670.	ipsc	Hepatocytes	Matrigel	Matrigel
123	33172680	Liang X, Yang R, Yin N, Faiola F. Evaluation of the effects of low nanomolar bisphenol A-like compounds' levels on early human embryonic development and lipid metabolism with human embryonic stem cell in vitro differentiation models. J Hazard Mater. 2021 Apr 5;407:124387.	ipsc	Hepatocytes	Vitronectin	Matrigel
124	33257114	Lee G, Kim H, Park JY, Kim G, Han J, Chung S, et al. Generation of uniform liver spheroids from human pluripotent stem cells for imaging-based drug toxicity analysis. Biomaterials. 2021 Feb;269:120529.	esc	Hepatocytes	Feeder	Matrigel
125	33657866	Fagg WS, Liu N, Patrikeev I, Saldarriaga OA, Motamedi M, Popov VL, et al. Endoderm and Hepatic Progenitor Cells Engraft in the Quiescent Liver Concurrent with Intrinsically Activated Epithelial-to-Mesenchymal Transition. Cell Transplant. 2021;30:963689721993780.	esc	Hepatocytes	Matrigel	Matrigel
126	33766737	Yuan F, Wang N, Chen Y, Huang X, Yang Z, Xu Y, et al. Calcitriol promotes the maturation of hepatocyte-like cells derived from human pluripotent stem cells. J Steroid Biochem Mol Biol. 2021 Jul;211:105881.	esc	Hepatocytes	Matrigel	Matrigel
127	34010638	Heslop JA, Pournasr B, Liu JT, Duncan SA. GATA6 defines endoderm fate by controlling chromatin accessibility during differentiation of human-induced pluripotent stem cells. Cell Rep. 2021 May 18;35(7):109145.	ipsc	Hepatocytes	hE-cad-Fc	Matrigel
128	35439850	Szeky B, Mayer B, Gyongy M, Hajdara A, Barsi S, Karpati S, et al. Tri-Lineage Differentiation of NTERA2 Clone D1 Cells towards Neural, Hepatic and Osteogenic Lineages in Vitro. Folia Biol (Praha). 2021;67(5–6):174–82.	ipsc	Hepatocytes	Matrigel	Matrigel
129	10.1016/j.molmet.2024.102057	Soares De Oliveira L, Kaserman JE, Van Der Spek AH, Lee NJ, Undeutsch HJ, Werder RB, et al. Thyroid hormone receptor beta (THRβ1) is the major regulator of T3 action in human iPSC-derived hepatocytes. Mol Metab. 2024 Dec;90:102057.	ipsc	Hepatocytes	Matrigel	Matrigel
130	10.36922/ijb.1403	Liang S, Luo Y, Su Y, Zhang D, Wang S jie, Xu M, et al. Distinct toxicity of microplastics/TBBPA co-exposure to bioprinted liver organoids derived from hiPSCs of healthy and patient donors. International Journal of Bioprinting. 2024 Jan 18;10(3):1403.	ipsc	Hepatocytes	Matrigel	Matrigel
131	40099268	Wang J, Huang D, Ren H, Zhao Y. Bioinspired Spatially Ordered Multicellular Lobules for Liver Regeneration. Research (Wash D C). 2025;8:0634.	ipsc	Hepatocytes	Matrigel	Matrigel
132	40027809	Prescott JB, Liu KJ, Lander A, Pek NMQ, Jha SK, Bokelmann M, et al. Metabolically purified human stem cell-derived hepatocytes reveal distinct effects of Ebola and Lassa viruses. bioRxiv. 2025 Feb 20;2025.02.17.638665.	ipsc	Hepatocytes	Matrigel	Matrigel

133	39988667	Bayarsaikhan D, Bayarsaikhan G, Lee J, Okano T, Kim K, Lee B. Development of iPSC-derived FIX-secreting hepatocyte sheet as a novel treatment tool for hemophilia B treatment. Stem Cell Res Ther. 2025 Feb 23;16(1):88.	ipsc	Hepatocytes	Matrigel	Matrigel
134	39933616	Sodani K, Ter Braak B, Hartvelt S, Boelens M, Jamalpoor A, Mukhi S. Toxicological mode-of-action and developmental toxicity of different carbon chain length PFAS. Toxicol Lett. 2025 Mar;405:59–66.	ipsc	Hepatocytes	Matrigel	Matrigel
135	39676795	Petazzi P, Gutierrez-Agüera F, Roca-Ho H, Castaño J, Bueno C, Alvarez N, et al. Generation of an inducible dCas9-SAM human PSC line for endogenous gene activation. Front Cell Dev Biol. 2024;12:1484955.	esc	Hepatocytes	Matrigel	Matrigel
136	39506029	Wang H, Danoy M, Gong Y, Utami T, Arakawa H, Kato Y, et al. Palmitic Acid Induced a Dedifferentiation Profile at the Transcriptome Level: A Collagen Synthesis but no Triglyceride Accumulation in Hepatocyte-Like Cells Derived From Human-Induced Pluripotent Stem Cells Cultivated Inside Organ on a Chip. J Appl Toxicol. 2025 Mar;45(3):460–71.	ipsc	Hepatocytes	Matrigel	Matrigel
137	39495460	Bayarsaikhan D, Bayarsaikhan G, Kang HA, Lee SB, Han SH, Okano T, et al. A Study on iPSC-Associated Factors in the Generation of Hepatocytes. Tissue Eng Regen Med. 2024 Dec;21(8):1245–54.	ipsc	Hepatocytes	Matrigel (GFR)	Matrigel
138	39336111	Zhang L, Wang X, Yang X, Chi Y, Chu Y, Zhang Y, et al. Genome Engineering of Primary and Pluripotent Stem Cell-Derived Hepatocytes for Modeling Liver Tumor Formation. Biology (Basel). 2024 Sep 2;13(9):684.	ipsc	Hepatocytes	Matrigel	Matrigel
139	39316725	Hu Y, Soares De Oliveira L, Falize K, Paul van Trotsenburg AS, Fliers E, Kaserman JE, et al. Disturbed function of TBL1X has a differential effect on T3-regulated gene expression in two human liver cell models. Eur Thyroid J. 2024 Oct 1;13(5):e240162.	ipsc	Hepatocytes	Matrigel (GFR)	Matrigel
140	39276457	Li H, Chen C, Huang W, Shi L, Zhang Q, Zhou L, et al. Long-term expanded hepatic progenitor cells ameliorate D-GalN/LPS-induced acute liver failure through repolarizing M1 macrophage to M2-Like phenotype via activation of the IL-10/JAK2/STAT3 signaling pathway. Int Immunopharmacol. 2024 Dec 5;142(Pt A):113127.	ipsc, esc	Hepatocytes	Matrigel	Matrigel
141	39266553	Qi L, Groeger M, Sharma A, Goswami I, Chen E, Zhong F, et al. Adipocyte inflammation is the primary driver of hepatic insulin resistance in a human iPSC-based microphysiological system. Nat Commun. 2024 Sep 12;15(1):7991.	ipsc	Hepatocytes	Matrigel	Matrigel
142	39232200	Chi H, Qu B, Prawira A, Richardt T, Maurer L, Hu J, et al. An hepatitis B and D virus infection model using human pluripotent stem cell-derived hepatocytes. EMBO Rep. 2024 Oct;25(10):4311–36.	ipsc	Hepatocytes	Matrigel	Matrigel
143	39208640	Wang J, Ye D, Li S, Lu X, Chi Y, Tang C, et al. Generation of human induced pluripotent stem cells carrying albumin-sfGFP reporter. Stem Cell Res. 2024 Dec;81:103543.	ipsc	Hepatocytes	Matrigel	Matrigel
144	39190693	Faccioli LAP, Sun Y, Animasahun O, Motomura T, Liu Z, Kurihara T, et al. Human-induced pluripotent stem cell-based hepatic modeling of lipid metabolism-associated TM6SF2-E167K variant. Hepatology. 2024 Aug 27;	ipsc	Hepatocytes	Matrigel	Matrigel
145	39000384	Doueiry C, Kappler CS, Martinez-Morant C, Duncan SA. A PNPLA3-Deficient iPSC-Derived Hepatocyte Screen Identifies Pathways to Potentially Reduce Steatosis in Metabolic Dysfunction-Associated Fatty Liver Disease. Int J Mol Sci. 2024 Jul 2;25(13):7277.	ipsc	Hepatocytes	Matrigel	Matrigel
146	38865994	Cools L, Dastjerd MK, Smout A, Merens V, Yang Y, Reynaert H, et al. Human iPSC-derived liver co-culture spheroids to model liver fibrosis. Biofabrication. 2024 Jun 28;16(3).	ipsc	Hepatocytes	Laminin 521	Matrigel
147	38615723	Chandrasekaran V, Wellens S, Bourguignon A, Djidrovski I, Fransen L, Ghosh S, et al. Evaluation of the impact of iPSC differentiation protocols on transcriptomic signatures. Toxicol In Vitro. 2024 Jun;98:105826.	ipsc	Hepatocytes	Matrigel	Matrigel
148	38586058	Shrestha S, Acharya P, Kang SY, Vanga MG, Lekkala VKR, Liu J, et al. Regenerative human liver organoids (HLOs) in a pillar/perfusion plate for hepatotoxicity assays. bioRxiv. 2024 Nov 3;2024.03.25.586638.	ipsc + esc	Hepatocytes	Matrigel (GFR)	Matrigel
149	38447229	Scheidecker B, Poulain S, Sugimoto M, Kido T, Kawanishi T, Miyajima A, et al. Dynamic, IPSC-derived hepatic tissue tri-culture system for the evaluation of liver physiologyin vitro. Biofabrication. 2024 Mar 25;16(2).	ipsc	Hepatocytes	Matrigel	Matrigel
150	38250613	Xu Y, Wang Y, Qi R, Li K, Wang X, Li X, et al. Role of connexin 32 in the directional differentiation of induced pluripotent stem cells into hepatocytes. Int J Med Sci. 2024;21(3):508–18.	ipsc	Hepatocytes	Matrigel	Matrigel
151	37995700	Zhu W, Li M, Zou J, Zhang D, Fang M, Sun Y, et al. Induction of local immunosuppression in allogeneic cell transplantation by cell-type-specific expression of PD-L1 and CTLA4Ig. Stem Cell Reports. 2023 Dec 12;18(12):2344–55.	esc	Hepatocytes	Matrigel	Matrigel
152	37907977	Farhan F, Trivedi M, Di Wu P, Cui W. Extracellular matrix modulates the spatial hepatic features in hepatocyte-like cells derived from human embryonic stem cells. Stem Cell Res Ther. 2023 Nov 1;14(1):314.	esc	Hepatocytes	Matrigel	Matrigel
153	37901872	Tomizawa M, Shinozaki F, Mikata T, Tanno H, Shigeta M. Lactate promotes survival and hepatocyte differentiation of human induced pluripotent stem cells in a medium without glucose and supplemented with galactose. Biomed Rep. 2023 Dec;19(6):90.	ipsc	Hepatocytes	Matrigel	Matrigel
154	37814474	Lv Y, Rao Z, Liu L, Jia J, Wu C, Xu J, et al. The efficient generation of functional human hepatocytes from chemically induced pluripotent stem cells. Cell Prolif. 2024 Feb;57(2):e13540.	ipsc	Hepatocytes	Matrigel	Matrigel
155	37626611	Calamaio S, Serzanti M, Boniotti J, Fra A, Garrafa E, Cominelli M, et al. Human iPSC-Derived 3D Hepatic Organoids in a Miniaturized Dynamic Culture System. Biomedicines. 2023 Jul 26;11(8):2114.	ipsc	Hepatocytes	Laminin 521	Matrigel
156	37511568	Trionfini P, Romano E, Varinelli M, Longaretti L, Rizzo P, Giampietro R, et al. Hypoimmunogenic Human Pluripotent Stem Cells as a Powerful Tool for Liver Regenerative Medicine. Int J Mol Sci. 2023 Jul 22;24(14):11810.	ipsc	Hepatocytes	Matrigel	Matrigel
157	37479967	Chitrangi S, Vaity P, Jamdar A, Bhatt S. Patient-derived organoids for precision oncology: a platform to facilitate clinical decision making. BMC Cancer. 2023 Jul 22;23(1):689.	ipsc	Hepatocytes	Matrigel	Matrigel
158	37240366	Tang Q, Hu Z, Zhao J, Zhou T, Tang S, Wang P, et al. CRISPR-Mediated In Situ Introduction or Integration of F9-Padua in Human iPSCs for Gene Therapy of Hemophilia B. Int J Mol Sci. 2023 May 19;24(10):9013.	ipsc	Hepatocytes	Matrigel	Matrigel
159	36691086	Chen Y, Zhou Y, Zhou Z, Fang Y, Ma L, Zhang X, et al. Hypoimmunogenic human pluripotent stem cells are valid cell sources for cell therapeutics with normal self-renewal and multilineage differentiation capacity. Stem Cell Res Ther. 2023 Jan 24;14(1):11.	esc	Hepatocytes	Matrigel	Matrigel
160	36481309	Tao F, Hanada S, Matsushima K, Arakawa H, Ishida N, Kato Y, et al. Enhancement and maintenance of hepatic metabolic functions by controlling 3D aggregation of cryopreserved human iPS cell-derived hepatocyte-like cells. J Biosci Bioeng. 2023 Feb;135(2):134–42.	ipsc	Hepatocytes	Matrigel	Matrigel
161	36404924	Florentino RM, Morita K, Haep N, Motomura T, Diaz-Aragon R, Faccioli LAP, et al. Biofabrication of synthetic human liver tissue with advanced programmable functions. iScience. 2022 Dec 22;25(12):105503.	ipsc	Hepatocytes	Matrigel	Matrigel
162	36251346	Tauran Y, Lereau-Bernier M, Segard BD, Danoy M, Kimura K, Shinohara M, et al. A novel agonist for the HGF receptor MET promotes differentiation of human pluripotent stem cells into hepatocyte-like cells. Dev Growth Differ. 2022 Dec;64(9):527–36.	ipsc	Hepatocytes	Matrigel	Matrigel
163	36056448	Wang C, Yu X, Ding S, Liu Y, Zhang H, Fu J, et al. Induced hepatic stem cells maintain self-renewal through the high expression of Myc coregulated by TET1 and CTCF. Cell Biosci. 2022 Sep 2;12(1):143.	ipsc	Hepatocytes	Gelatin	Matrigel
164	35453693	Jeong J, Kim TH, Kim M, Jung YK, Kim KS, Shim S, et al. Elimination of Reprogramming Transgenes Facilitates the Differentiation of Induced Pluripotent Stem Cells into Hepatocyte-like Cells and Hepatic Organoids. Biology (Basel). 2022 Mar 23;11(4):493.	ipsc	Hepatocytes	Matrigel	Matrigel
165	34877514	Wei R, Yang J, Cheng CW, Ho WI, Li N, Hu Y, et al. CRISPR-targeted genome editing of human induced pluripotent stem cell-derived hepatocytes for the treatment of Wilson's disease. JHEP Rep. 2022 Jan;4(1):100389.	ipsc	Hepatocytes	Matrigel	Matrigel
166	34830064	Völkner C, Liedtke M, Untucht R, Hermann A, Frech MJ. Patient-Specific iPSC-Derived Neural Differentiated and Hepatocyte-like Cells, Carrying the Compound Heterozygous Mutation p.V1023Sfs*15/p.G992R, Present the 'Variant' Biochemical Phenotype of Niemann-Pick Type C1 Disease. Int J Mol Sci. 2021 Nov 10;22(22):12184.	ipsc	Hepatocytes	Matrigel	Matrigel

167	34602548	Kikuchi C, Sakasai-Sakai A, Okimura R, Tanaka H, Takata T, Takeuchi M, et al. Accumulation of Toxic Advanced Glycation End-Products Induces Cytotoxicity and Inflammation in Hepatocyte-Like Cells Differentiated from Human Induced Pluripotent Stem Cells. Biol Pharm Bull. 2021;44(10):1399–402.	ipsc	Hepatocytes	Matrigel	Matrigel
168	34027283	Danoy M, Tauran Y, Poulain S, Jellali R, Bruce J, Leduc M, et al. Investigation of the hepatic development in the coculture of hiPSCs-derived LSECs and HLCs in a fluidic microenvironment. APL Bioeng. 2021 Jun;5(2):026104.	ipsc	Hepatocytes	Matrigel	Matrigel
169	33545341	Ter Braak B, Niemeijer M, Boon R, Parmentier C, Baze A, Richert L, et al. Systematic transcriptome-based comparison of cellular adaptive stress response activation networks in hepatic stem cell-derived progeny and primary human hepatocytes. Toxicol In Vitro. 2021 Jun;73:105107.	ipsc	Hepatocytes	Matrigel	Matrigel
170	33530582	Zabulica M, Jakobsson T, Ravaioli F, Vosough M, Gramignoli R, Ellis E, et al. Gene Editing Correction of a Urea Cycle Defect in Organoid Stem Cell Derived Hepatocyte-like Cells. Int J Mol Sci. 2021 Jan 26;22(3):1217.	ipsc	Hepatocytes	Vitronectin	Matrigel
171	33083764	Tanosaki S, Tohyama S, Fujita J, Someya S, Hishiki T, Matsuura T, et al. Fatty Acid Synthesis Is Indispensable for Survival of Human Pluripotent Stem Cells. iScience. 2020 Sep 25;23(9):101535.	ipsc	Hepatocytes	Matrigel	Matrigel
172	33015019	Yoshimoto K, Minier N, Yang J, Imamura S, Stocking K, Patel J, et al. Recapitulation of Human Embryonic Heartbeat to Promote Differentiation of Hepatic Endoderm to Hepatoblasts. Front Bioeng Biotechnol. 2020;8:568092.	esc	Hepatocytes	Matrigel	Matrigel
173	32323516	Mun SJ, Hong YH, Ahn HS, Ryu JS, Chung KS, Son MJ. Long-Term Expansion of Functional Human Pluripotent Stem Cell-Derived Hepatic Organoids. Int J Stem Cells. 2020 Jul 30;13(2):279–86.	msc	Hepatocytes	Matrigel	Matrigel
174	32302844	Hu B, Yin N, Yang R, Liang S, Liang S, Faiola F. Silver nanoparticles (AgNPs) and AgNO3 perturb the specification of human hepatocyte-like cells and cardiomyocytes. Sci Total Environ. 2020 Jul 10;725:138433.	esc	Hepatocytes	Vitronectin	Matrigel
175	32151917	Kim A, Lee SY, Seo CS, Chung SK. Ethanol extract of Magnoliae cortex (EEMC) limits teratoma formation of pluripotent stem cells by selective elimination of undifferentiated cells through the p53-dependent mitochondrial apoptotic pathway. Phytomedicine. 2020 Apr;69:153198.	ipsc	Hepatocytes	Matrigel	Matrigel
176	39884160	Surendran H, Battu R, Gopurappilly R, Vishnuprasad CN, Pal R. Generation of a human induced pluripotent stem cell (iPSC) line ERPLi004-A from an Alpha-1 antitrypsin deficiency (AATD) patient with SERPINA1 mutation. Stem Cell Res. 2025 Mar;83:103664.	ipsc	Hepatocytes	Matrigel	Matrigel
177	39559558	Mitani S, Hosoda C, Onodera Y, Takabayashi Y, Sakata A, Shima M, et al. Efficient generation of liver sinusoidal endothelial-like cells secreting coagulation factor VIII from human induced pluripotent stem cells. Mol Ther Methods Clin Dev. 2024 Dec 12;32(4):101355.	ipsc	Hepatocytes	Matrigel	Matrigel
178	39532815	Ge N, Suzuki K, Sato I, Noguchi M, Nakamura Y, Matsuo-Takasaki M, et al. Generation of human induced pluripotent stem cell lines derived from patients of cystic biliary atresia. Hum Cell. 2024 Nov 13;38(1):18.	ipsc	Hepatocytes	Laminin 511-Silk	Matrigel
179	39416072	Rezvani M, Lewis K, Quach S, Iwasawa K, Weihs J, Reza H, et al. Fetal Liver-like Organoids Recapitulate Blood-Liver Niche Development and Multipotent Hematopoiesis from Human Pluripotent Stem Cells. bioRxiv. 2024 Oct 12;2024.10.11.617794.	ipsc	Hepatocytes	Matrigel (GFR)	Matrigel
180	38694820	Loerch C, Szepanowski LP, Reiss J, Adjaye J, Graffmann N. Forskolin induces FXR expression and enhances maturation of iPSC-derived hepatocyte-like cells. Front Cell Dev Biol. 2024;12:1383928.	ipsc	Hepatocytes	Matrigel	Matrigel
181	38668386	Jin J, Nguyen LTG, Wassef A, Sadek R, Schmitt TM, Guo GL, et al. Identification and Functional Characterization of Alternative Transcripts of LncRNA HNF1A-AS1 and Their Impacts on Cell Growth, Differentiation, Liver Diseases, and in Response to Drug Induction. Noncoding RNA. 2024 Apr 21;10(2):28.	esc	Hepatocytes	Matrigel	Matrigel
182	36272632	Li S, Yang R, Yin N, Zhao M, Zhang S, Faiola F. Developmental toxicity assessments for TBBPA and its commonly used analogs with a human embryonic stem cell liver differentiation model. Chemosphere. 2023 Jan;310:136924.	ipsc	Hepatocytes	Vitronectin	Matrigel
183	35863744	Muñoz A, Theusch E, Kuang YL, Nalula G, Peaslee C, Dorlhiac G, et al. Undifferentiated Induced Pluripotent Stem Cells as a Genetic Model for Nonalcoholic Fatty Liver Disease. Cell Mol Gastroenterol Hepatol. 2022;14(5):1174-1176.e6.	ipsc	Hepatocytes	Matrigel (GFR)	Matrigel
184	35451583	Ahn H, Ryu J, Lee J, Mun SJ, Hong Y, Shin Y, et al. Generation of An Induced Pluripotent Stem Cell Line from Human Liver Fibroblasts from A Patient with Combined Hepatocellular-Cholangiocarcinoma. Cell J. 2022 Mar;24(3):133–9.	ipsc	Hepatocytes	Feeder	Matrigel
185	35182771	Nunes C, Singh P, Mazidi Z, Murphy C, Bourguignon A, Wellens S, et al. An in vitro strategy using multiple human induced pluripotent stem cell-derived models to assess the toxicity of chemicals: A case study on paraquat. Toxicol In Vitro. 2022 Jun;81:105333.	ipsc	Hepatocytes	Matrigel (GFR)	Matrigel
186	34765922	Zhang Y, Guo A, Lyu C, Bi R, Wu Z, Li W, et al. Synthetic liver fibrotic niche extracts achieve in vitro hepatoblasts phenotype enhancement and expansion. iScience. 2021 Nov 19;24(11):103303.	esc	Hepatocytes	Matrigel	Matrigel
187	34017432	Chen Y, Li R, Zhang L, Gan L, Ding J. Treatment of α-1 antitrypsin deficiency using hepatic-specified cells derived from human-induced pluripotent stem cells. Am J Transl Res. 2021;13(4):2710–6.	ipsc	Hepatocytes	Matrigel	Matrigel
188	32914807	Wang Y, Liu H, Zhang M, Wang H, Chen W, Qin J. One-step synthesis of composite hydrogel capsules to support liver organoid generation from hiPSCs. Biomater Sci. 2020 Oct 7;8(19):5476–88.	ipsc	Hepatocytes	Matrigel	Matrigel
189	37663646	Shintani T, Imamura C, Ueyama-Toba Y, Inui J, Watanabe A, Mizuguchi H. Establishment of UGT1A1-knockout human iPS-derived hepatic organoids for UGT1A1-specific kinetics and toxicity evaluation. Mol Ther Methods Clin Dev. 2023 Sep 14;30:429–42.	ipsc	Hepatocytes, Liver organoids	Laminin 511	Matrigel
190	36089014	Cheng W, Zhou Y, Xie Y, Li Y, Zhou R, Wang H, et al. Combined effect of polystyrene microplastics and bisphenol A on the human embryonic stem cells-derived liver organoids: The hepatotoxicity and lipid accumulation. Sci Total Environ. 2023 Jan 1;854:158585.	esc	Hepatocytes, Liver organoids	Matrigel	Matrigel
191	39213921	Cheng W, Chen H, Zhou Y, You Y, Lei D, Li Y, et al. Aged fragmented-polypropylene microplastics induced ageing statues-dependent bioenergetic imbalance and reductive stress: In vivo and liver organoids-based in vitro study. Environ Int. 2024 Sep;191:108949.	esc	Hepatocytes, Liver organoids	Matrigel	Matrigel
192	38184815	Utami T, Danoy M, Khadim RR, Tokito F, Arakawa H, Kato Y, et al. A highly efficient cell culture method using oxygen-permeable PDMS-based honeycomb microwells produces functional liver organoids from human induced pluripotent stem cell-derived carboxypeptidase M liver progenitor cells. Biotechnol Bioeng. 2024 Apr;121(4):1178–90.	ipsc	Hepatocytes, Liver organoids	Vitroectin vs. Matrigel	Matrigel
193	32923835	Abbey D, Elwyn S, Hand NJ, Musunuru K, Rader DJ. Self-Organizing Human Induced Pluripotent Stem Cell Hepatocyte 3D Organoids Inform the Biology of the Pleiotropic TRIB1 Gene. Hepatol Commun. 2020 Sep;4(9):1316–31.	ipsc	Hepatocytes, Liver organoids	Matrigel (GFR)	Matrigel
194	36541471	Deng S, Zhao X, Zhu Y, Tang N, Wang R, Zhang X, et al. Efficient hepatic differentiation of hydrogel microsphere-encapsulated human pluripotent stem cells for engineering prevascularized liver tissue. Biofabrication. 2022 Dec 16;15(1).	esc	Hepatocytes	Matrigel	Matrigel vs. Collagen-1
195	39729989	Zhao Z, Zeng F, Nie Y, Lu G, Xu H, En H, et al. Chemically defined and growth factor-free system for highly efficient endoderm induction of human pluripotent stem cells. Stem Cell Reports. 2025 Jan 14;20(1):102382.	esc	Hepatocytes	Matrigel vs. Vitronectin vs. Synthemax II-SC	Matrigel vs. Synthemax II-SC
196	34858546	Mobarra N, Raji S, Najafi S, Kafi FK, Ferns GA, Pakzad R. Hypoxia-Induced miR-210 Overexpression Promotes the Differentiation of Human-Induced Pluripotent Stem Cells to Hepatocyte-Like Cells on Random Nanofiber Poly-L-Lactic Acid/Poly (ε-Caprolactone) Scaffolds. Oxid Med Cell Longev. 2021;2021:4229721.	ipsc	Hepatocytes	Feeder	PLLA/PCL fibres
197	36098216	Parvanak M, Mostafavi-Pour Z, Soleimani M, Atashi A, Arefian E, Esmaeili E. Mir-122 upregulation and let-7f downregulation combination: The effects on hepatic differentiation of hiPSCs on the PCL-Gel-HA nanofibrous scaffold. J Cell Mol Med. 2022 Oct;26(20):5235–45.	ipsc	Hepatocytes	Feeder	PCL-Gel-HA
198	37515966	da Silva Nunes Barreto R, da Silva Júnior LN, Henrique Doná Rodrigues Almeida G, de Oliveira Horvath-Pereira B, da Silva TS, Garcia JM, et al. Placental scaffolds as a potential biological platform for embryonic stem cells differentiation into hepatic-like cells lineage: A pilot study. Tissue Cell. 2023 Oct;84:102181.	esc	Hepatocytes	Placenta	Placenta
199	32840229	Murchison AC, Odanga JJ, Treadwell ML, Breathwaite EK, Weaver JR, Lee JB. Human Placenta-Derived ECM Supports Tri-Lineage Differentiation of Human Induced Pluripotent Stem Cells. Int J Stem Cells. 2020 Nov 30;13(3):432–8.	ipsc	Hepatocytes	Matrigel	Placenta - Hugentra

200	33876166	Luo C, Lü D, Zheng L, Zhang F, Zhang X, Lü S, et al. Hepatic differentiation of human embryonic stem cells by coupling substrate stiffness and microtopography. Biomater Sci. 2021 May 18;9(10):3776–90.	esc	Hepatocytes	Matrigel	Polyacrylamide
201	34217893	Werder RB, Kaserman JE, Packer MS, Lindstrom-Vautrin J, Villacorta-Martin C, Young LE, et al. Adenine base editing reduces misfolded protein accumulation and toxicity in alpha-1 antitrypsin deficient patient iPSC-hepatocytes. Mol Ther. 2021 Nov 3;29(11):3219–29.	ipsc	Hepatocytes	Matrigel (GFR)	Matrigel (GFR)
202	39427033	Vicente P, Almeida JI, Crespo IE, Virgolini N, Isidro IA, Calleja-Cervantes ME, et al. Oxygen control in bioreactor drives high yield production of functional hiPSC-like hepatocytes for advanced liver disease modelling. Sci Rep. 2024 Oct 19;14(1):24599.	ipsc	Hepatocytes	Cellartis DEF COAT-1	Suspension Culture
203	36105133	He J, Wang J, Pang Y, Yu H, Qin X, Su K, et al. Bioprinting of a Hepatic Tissue Model Using Human-Induced Pluripotent Stem Cell-derived Hepatocytes for Drug-Induced Hepatotoxicity Evaluation. Int J Bioprint. 2022;8(3):581.	ipsc	Hepatocytes	Suspension Culture	Suspension Culture
204	38092880	Weber J, Linti C, Lörch C, Weber M, Andt M, Schlensak C, et al. Combination of melt-electrospun poly-ε-caprolactone scaffolds and hepatocyte-like cells from footprint-free hiPSCs to create 3D biohybrid constructs for liver tissue engineering. Sci Rep. 2023 Dec 13;13(1):22174.	ipsc	Hepatocytes	Vitronectin	Vitronectin
205	39094505	Tang P, Keshi E, Wilken S, Wutsdorff L, Mougnekabol J, Pratschke J, et al. Generation of an induced pluripotent stem cell (iPSC) line (EXSURGi001-A) from a patient homozygous for the p.Ala165Thr mutation in the MTARC1 gene. Stem Cell Res. 2024 Oct;80:103516.	ipsc	Hepatocytes	Vitronectin	Vitronectin
206	39036087	Roudaut M, Caillaud A, Souguir Z, Bray L, Girardeau A, Rimbert A, et al. Human induced pluripotent stem cells-derived liver organoids grown on a Biomimesys® hyaluronic acid-based hydroscaffold as a new model for studying human lipoprotein metabolism. Bioeng Transl Med. 2024 Jul;9(4):e10659.	ipsc	Liver organoids	Matrigel (GFR)	Biomimesys
207	38516405	Naito C, Kosar K, Kishimoto E, Pena L, Huang Y, Hao K, et al. Induced pluripotent stem cell (iPSC) modeling validates reduced GBE1 enzyme activity due to a novel variant, p.Ile694Asn, found in a patient with suspected glycogen storage disease IV. Mol Genet Metab Rep. 2024 Jun;39:101069.	ipsc	Liver organoids	Laminin 511	Laminin 511-Silk
208	38451817	Li Y, Nie Y, Yang X, Liu Y, Deng X, Hayashi Y, et al. Integration of Kupffer cells into human iPSC-derived liver organoids for modeling liver dysfunction in sepsis. Cell Rep. 2024 Mar 26;43(3):113918.	ipsc	Liver organoids	Laminin 511-Silk	Laminin 511-Silk
209	38110841	Septiana WL, Ayudyasari W, Gunardi H, Pawitan JA, Balachander GM, Yu H, et al. Liver organoids cocultured on decellularized native liver scaffolds as a bridging therapy improves survival from liver failure in rabbits. In Vitro Cell Dev Biol Anim. 2023 Dec;59(10):747–63.	ipsc	Liver organoids	Vitronectin	Decellularised Rabbit Liver
210	33672150	Mori A, Murata S, Tashiro N, Tadokoro T, Okamoto S, Otsuka R, et al. Establishment of Human Leukocyte Antigen-Mismatched Immune Responses after Transplantation of Human Liver Bud in Humanized Mouse Models. Cells. 2021 Feb 23;10(2):476.	ipsc	Liver organoids	Laminin 511	Elplasia
211	35159346	Messina A, Luce E, Benzoubir N, Pasqua M, Pereira U, Humbert L, et al. Evidence of Adult Features and Functions of Hepatocytes Differentiated from Human Induced Pluripotent Stem Cells and Self-Organized as Organoids. Cells. 2022 Feb 4;11(3):537.	ipsc	Liver organoids	Feeder	Gelatin
212	40032498	Li G, He J, Shi J, Li X, Liu L, Ge X, et al. Bioprinting functional hepatocyte organoids derived from human chemically induced pluripotent stem cells to treat liver failure. Gut. 2025 Mar 3;gutjnl-2024-333885.	ipsc	Liver organoids	Matrigel	GelMA
213	39621053	Wu X, Jiang D, Wang Y, Li X, Liu C, Chen Y, et al. Modeling metabolic-associated steatohepatitis with human pluripotent stem cell-derived liver organoids. Hepatol Commun. 2024 Dec 1;8(12):e0585.	ipsc	Liver organoids	Laminin 511	Laminin 511
214	39047116	Tadokoro T, Murata S, Kato M, Ueno Y, Tsuchida T, Okumura A, et al. Human iPSC-liver organoid transplantation reduces fibrosis through immunomodulation. Sci Transl Med. 2024 Jul 24;16(757):eadg0338.	ipsc	Liver organoids	Laminin 511	Laminin 511 then suspension culture
215	37546982	Sun C, Zhou C, Daneshvar K, Kratkiewicz AJ, Saad AB, Hess A, et al. Conserved long noncoding RNA TILAM promotes liver fibrosis through interaction with PML in hepatic stellate cells. bioRxiv. 2023 Jul 30;2023.07.29.551032.	esc	Liver organoids	Matrigel	Laminin 521
216	36032707	Xu X, Jiang S, Gu L, Li B, Xu F, Li C, et al. High-throughput bioengineering of homogenous and functional human-induced pluripotent stem cells-derived liver organoids via micropatterning technique. Front Bioeng Biotechnol. 2022;10:937595.	ipsc	Liver organoids	Vitronectin	PEG
217	39755926	Fan H, Shang J, Li J, Yang B, Zhou D, Jiang S, et al. High-Throughput Formation of Pre-Vascularized hiPSC-Derived Hepatobiliary Organoids on a Chip via Nonparenchymal Cell Grafting. Adv Sci (Weinh). 2025 Feb;12(8):e2407945.	ipsc	Liver organoids	Vitronectin	Suspension Culture
218	36456801	Torizal FG, Utami T, Lau QY, Inamura K, Nishikawa M, Sakai Y. Dialysis based-culture medium conditioning improved the generation of human induced pluripotent stem cell derived-liver organoid in a high cell density. Sci Rep. 2022 Dec 1;12(1):20774.	ipsc	Liver organoids	Vitronectin	Suspension Culture
219	38985547	Kogler S, Pedersen GM, Martínez-Ramírez F, Aizenshtadt A, Busek M, Krauss SJK, et al. An FDA-Validated, Self-Cleaning Liquid Chromatography-Mass Spectrometry System for Determining Small-Molecule Drugs and Metabolites in Organoid/Organ-on-Chip Medium. Anal Chem. 2024 Jul 23;96(29):12129–38.	esc	Liver organoids	Matrigel (GFR)	Suspension Culture
220	38529852	Harrison SP, Baumgarten SF, Chollet ME, Stavik B, Bhattacharya A, Almaas R, et al. Parenteral nutrition emulsion inhibits CYP3A4 in an iPSC derived liver organoids testing platform. J Pediatr Gastroenterol Nutr. 2024 May;78(5):1047–58.	ipsc	Liver organoids	Matrigel (GFR) vs. Vitronectin	Suspension Culture
221	35217482	Son JS, Park CY, Lee G, Park JY, Kim HJ, Kim G, et al. Therapeutic correction of hemophilia A using 2D endothelial cells and multicellular 3D organoids derived from CRISPR/Cas9-engineered patient iPSCs. Biomaterials. 2022 Apr;283:121429.	ipsc	Liver organoids	Feeder	Fibronectin then suspension Culture
222	34063948	Zahmatkesh E, Ghanian MH, Zarkesh I, Farzaneh Z, Halvaei M, Heydari Z, et al. Tissue-Specific Microparticles Improve Organoid Microenvironment for Efficient Maturation of Pluripotent Stem-Cell-Derived Hepatocytes. Cells. 2021 May 21;10(6):1274.	ipsc	Liver organoids	Matrigel	Suspension Culture
223	40082402	Kuse Y, Matsumoto S, Tsuzuki S, Carolina E, Okumura T, Kasai T, et al. Placenta-derived factors contribute to human iPSC-liver organoid growth. Nat Commun. 2025 Mar 13;16(1):2493.	ipsc	Liver organoids	Matrigel	Collagen-1 + Matrigel
224	39726370	Lekkala VKR, Shrestha S, Al Qaryoute A, Dhinoja S, Acharya P, Raheem A, et al. Enhanced Maturity and Functionality of Vascular Human Liver Organoids through 3D Bioprinting and Pillar Plate Culture. ACS Biomater Sci Eng. 2025 Jan 13;11(1):506–17.	ipsc	Liver organoids	Matrigel (GFR)	Matrigel (GFR)
225	38749417	Jo S, Park SB, Kim H, Im I, Noh H, Kim EM, et al. hiPSC-derived macrophages improve drug sensitivity and selectivity in a macrophage-incorporating organoid culture model. Biofabrication. 2024 May 28;16(3).	ipsc	Liver organoids	Matrigel	Matrigel (GFR)
226	38221504	Aizenshtadt A, Wang C, Abadpour S, Menezes PD, Wilhelmsen I, Dalmao-Fernandez A, et al. Pump-Less, Recirculating Organ-on-Chip (rOoC) Platform to Model the Metabolic Crosstalk between Islets and Liver. Adv Healthc Mater. 2024 May;13(13):e2303785.	esc	Liver organoids	Matrigel (GFR)	Matrigel (GFR)
227	37628828	Gomez-Mariano G, Perez-Luz S, Ramos-Del Saz S, Matamala N, Hernandez-SanMiguel E, Fernandez-Prieto M, et al. Acid Sphingomyelinase Deficiency Type B Patient-Derived Liver Organoids Reveals Altered Lysosomal Gene Expression and Lipid Homeostasis. Int J Mol Sci. 2023 Aug 10;24(16):12645.	LSC	Liver organoids	Matrigel (GFR)	Matrigel (GFR)
228	37380087	Kømurcu KS, Wilhelmsen I, Thorne JL, Krauss S, Wilson SR, Aizenshtadt A, et al. Mass spectrometry reveals that oxysterols are secreted from non-alcoholic fatty liver disease induced organoids. J Steroid Biochem Mol Biol. 2023 Sep;232:106355.	esc	Liver organoids	Matrigel (GFR)	Matrigel (GFR)
229	37159662	LaLone V, Aizenshtadt A, Goertz J, Skottvoll FS, Mota MB, You J, et al. Quantitative chemometric phenotyping of three-dimensional liver organoids by Raman spectral imaging. Cell Rep Methods. 2023 Apr 24;3(4):100440.	ipsc	Liver organoids	Matrigel (GFR)	Matrigel (GFR)
230	33534551	Skottvoll FS, Hansen FA, Harrison S, Boger IS, Mrsa A, Restan MS, et al. Electromembrane Extraction and Mass Spectrometry for Liver Organoid Drug Metabolism Studies. Anal Chem. 2021 Feb 23;93(7):3576–85.	esc	Liver organoids	Matrigel (GFR)	Matrigel (GFR)
231	39974651	Noh H, Choi S, Park KW, Lee S, Seok DW, Kim YE, et al. Amino Acid Hepatotoxicity Biomarkers in Human Hepatic Organoids: Promising Standardization of Drug Toxicity Evaluation. ACS Pharmacol Transl Sci. 2025 Feb 14;8(2):510–21.	ipsc	Liver organoids	Vitronectin	Matrigel (GFR)
232	33264615	Michielin F, Giobbe GG, Luni C, Hu Q, Maroni I, Orford MR, et al. The Microfluidic Environment Reveals a Hidden Role of Self-Organizing Extracellular Matrix in Hepatic Commitment and Organoid Formation of hiPSCs. Cell Rep. 2020 Dec 1;33(9):108453.	esc	Liver organoids	Matrigel (GFR)	Matrigel (GFR)

233	39198465	Carolina E, Kuse Y, Okumura A, Aoshima K, Tadokoro T, Matsumoto S, et al. Generation of human iPSC-derived 3D bile duct within liver organoid by incorporating human iPSC-derived blood vessel. Nat Commun. 2024 Aug 28;15(1):7424.	ipsc	Liver organoids	Laminin 511	Matrigel (GFR) + Collagen-1
234	39656528	Guan Y, Fang Z, Hu A, Roberts S, Wang M, Ren W, et al. Live-cell imaging of human liver fibrosis using hepatic micro-organoids. JCI Insight. 2024 Dec 10;10(2):e187099.	ipsc	Liver organoids	Matrigel	Matrigel
235	39696644	Zhang S, Liu L, Li X, Zhou T, Shi Q, Li D, et al. Transcriptomic and proteomic sequencing unveils the role of vitamin D and metabolic flux shifts in the induction of human hepatic organoids. Stem Cell Res Ther. 2024 Dec 18;15(1):478.	esc	Liver organoids	Matrigel	Matrigel
236	39447308	Chi KY, Kim G, Kim H, Kim H, Jo S, Lee J, et al. Optimization of culture conditions to generate vascularized multi-lineage liver organoids with structural complexity and functionality. Biomaterials. 2025 Mar;314:122898.	ipsc	Liver organoids	Matrigel	Matrigel
237	36128218	Richards A, Friesen M, Khalil A, Barrasa MI, Gehrke L, Jaenisch R. SARS-CoV-2 infection of human pluripotent stem cell-derived liver organoids reveals potential mechanisms of liver pathology. iScience. 2022 Oct 21;25(10):105146.	ipsc	Liver organoids	Matrigel	Matrigel
238	33632328	Pan T, Tao J, Chen Y, Zhang J, Getachew A, Zhuang Y, et al. Robust expansion and functional maturation of human hepatoblasts by chemical strategy. Stem Cell Res Ther. 2021 Feb 25;12(1):151.	esc, ipsc	Liver organoids	Matrigel	Matrigel
239	10.35848/1882-0786/abaeb5	Kobayashi N, Togo S, Matsuzaki T, Hashiseko K, Kawamura R, Suganuma M, et al. Stiffness distribution analysis in indentation depth direction reveals clear mechanical features of cells and organoids by using AFM. Appl Phys Express. 2020 Aug;13(9):097001.	ipsc	Liver organoids	Matrigel	Matrigel
240	10.1039/d4lc00149d	Shrestha S, Lekkala VKR, Acharya P, Kang SY, Vanga MG, Lee MY. Reproducible generation of human liver organoids (HLOs) on a pillar plate platform via microarray 3D bioprinting. Lab Chip. 2024 May 14;24(10):2747–61.	LSC	Liver organoids	Matrigel (GFR)	Matrigel
241	39921573	Wu H, Wang J, Liu S, Wang Y, Tang X, Xie J, et al. Large-Scale Production of Expandable Hepatoblast Organoids and Polarised Hepatocyte Organoids From hESCs Under 3D Static and Dynamic Suspension Conditions. Cell Prolif. 2025 Feb 8;e70001.	esc	Liver organoids	Feeder	Matrigel
242	39904185	Inui J, Ueyama-Toba Y, Imamura C, Nagai W, Asano R, Mizuguchi H. Two-dimensionally cultured functional hepatocytes generated from human induced pluripotent stem cell-derived hepatic organoids for pharmaceutical research. Biomaterials. 2025 Jul;318:123148.	ipsc	Liver organoids	Laminin 511	Matrigel
243	39716909	Wang J, Huang D, Ren H, Zhao Y. Biomimetic Vascularized iPSC-Hepatocyte Spheroids for Liver Regeneration. Adv Sci (Weinh). 2025 Feb;12(6):e2405662.	ipsc	Liver organoids	Matrigel	Matrigel
244	39639330	Nitaramorn N, Kobpornchai P, Tongkrajang N, Chaisri U, Imwong M, Kulkeaw K. Human liver organoids are susceptible to Plasmodium vivax infection. Malar J. 2024 Dec 5;23(1):368.	ipsc	Liver organoids	Matrigel (GFR)	Matrigel
245	39529647	Meyer SR, Zhang CJ, Garcia MA, Procario MC, Yoo S, Jolly AL, et al. A High-Throughput Microphysiological Liver Chip System to Model Drug-Induced Liver Injury Using Human Liver Organoids. Gastro Hep Adv. 2024;3(8):1045–53.	ipsc	Liver organoids	Matrigel (GFR)	Matrigel
246	39257824	Al Reza H, Santangelo C, Al Reza A, Iwasawa K, Sachiko S, Glaser K, et al. Self-Assembled Generation of Multi-zonal Liver Organoids from Human Pluripotent Stem Cells. bioRxiv. 2024 Aug 30;2024.08.30.610426.	ipsc	Liver organoids	Laminin 511	Matrigel
247	38815455	Wang L, Koui Y, Kanegae K, Kido T, Tamura-Nakano M, Yabe S, et al. Establishment of human induced pluripotent stem cell-derived hepatobiliary organoid with bile duct for pharmaceutical research use. Biomaterials. 2024 Oct;310:122621.	ipsc	Liver organoids	Matrigel	Matrigel
248	38104501	Jin ZL, Xu K, Kim J, Guo H, Yao X, Xu YN, et al. 3D hepatic organoid production from human pluripotent stem cells. Differentiation. 2024;135:100742.	ipsc, esc	Liver organoids	Matrigel	Matrigel
249	34686668	Guan Y, Enejder A, Wang M, Fang Z, Cui L, Chen SY, et al. A human multi-lineage hepatic organoid model for liver fibrosis. Nat Commun. 2021 Oct 22;12(1):6138.	ipsc	Liver organoids	Matrigel	Matrigel
250	32553762	Ramli MNB, Lim YS, Koe CT, Demircioglu D, Tng W, Gonzales KAU, et al. Human Pluripotent Stem Cell-Derived Organoids as Models of Liver Disease. Gastroenterology. 2020 Oct;159(4):1471-1486.e12.	ipsc	Liver organoids	Matrigel	Matrigel
251	40155664	Shin DS, Yang JY, Jeong HN, Mun SJ, Kim H, Son MJ, et al. Hepatotoxicity evaluation method through multiple-factor analysis using human pluripotent stem cell derived hepatic organoids. Sci Rep. 2025 Mar 28;15(1):10804.	ipsc	Liver organoids	Matrigel	Matrigel
252	39005378	Saiki N, Nio Y, Yoneyama Y, Kawamura S, Iwasawa K, Kawakami E, et al. Self-Organization of Sinusoidal Vessels in Pluripotent Stem Cell-derived Human Liver Bud Organoids. bioRxiv. 2024 Jul 4;2024.07.02.601804.	ipsc	Liver organoids	Laminin 511	Matrigel
253	38787150	Choi SY, Kim TH, Kim MJ, Mun SJ, Kim TS, Jung KK, et al. Validating Well-Functioning Hepatic Organoids for Toxicity Evaluation. Toxics. 2024 May 17;12(5):371.	ipsc	Liver organoids	Matrigel	Matrigel
254	38385067	Li H, Li J, Wang T, Sun K, Huang G, Cao Y, et al. Hepatobiliary organoids differentiated from hiPSCs relieve cholestasis-induced liver fibrosis in nonhuman primates. Int J Biol Sci. 2024;20(4):1160–79.	ipsc	Liver organoids	Matrigel	Matrigel
255	38129682	Mun SJ, Hong YH, Shin Y, Lee J, Cho HS, Kim DS, et al. Efficient and reproducible generation of human induced pluripotent stem cell-derived expandable liver organoids for disease modeling. Sci Rep. 2023 Dec 22;13(1):22935.	ipsc	Liver organoids	Feeder	Matrigel
256	37832542	Reza HA, Farooqui Z, Reza AA, Conroy C, Iwasawa K, Ogura Y, et al. Synthetic augmentation of bilirubin metabolism in human pluripotent stem cell-derived liver organoids. Stem Cell Reports. 2023 Nov 14;18(11):2071–83.	ipsc	Liver organoids	Laminin 511-silk	Matrigel
257	36864321	Wu X, Jiang D, Yang Y, Li S, Ding Q. Modeling drug-induced liver injury and screening for anti-hepatofibrotic compounds using human PSC-derived organoids. Cell Regen. 2023 Mar 3;12(1):6.	ipsc	Liver organoids	Matrigel (GFR)	Matrigel
258	34571217	Cheng W, Li X, Zhou Y, Yu H, Xie Y, Guo H, et al. Polystyrene microplastics induce hepatotoxicity and disrupt lipid metabolism in the liver organoids. Sci Total Environ. 2022 Feb 1;806(Pt 1):150328.	ipsc	Liver organoids	Matrigel	Matrigel
259	39263327	Correia de Sousa M, Delangre E, Berthou F, El Harane S, Maeder C, Fournier M, et al. Hepatic miR-149-5p upregulation fosters steatosis, inflammation and fibrosis development in mice and in human liver organoids. JHEP Rep. 2024 Sep;6(9):101126.	esc	Liver organoids	Biosilk (Laminin 521-silk)	Matrigel
260	37920068	Kuboyama-Sasaki A, Takahashi Y, Xia C, Hiro K, Kobayashi T, Ohdan H, et al. Establishment of a cell culture platform for human liver organoids and its application for lipid metabolism research. Biotechnol J. 2024 Jan;19(1):e2300365.	ipsc	Liver organoids	Matrigel	Matrigel
261	37680467	Kim JH, Mun SJ, Kim JH, Son MJ, Kim SY. Integrative analysis of single-cell RNA-seq and ATAC-seq reveals heterogeneity of induced pluripotent stem cell-derived hepatic organoids. iScience. 2023 Sep 15;26(9):107675.	ipsc	Liver organoids	Matrigel	Matrigel
262	37653039	Harrison SP, Siller R, Tanaka Y, Chollet ME, de la Morena-Barrio ME, Xiang Y, et al. Scalable production of tissue-like vascularized liver organoids from human PSCs. Exp Mol Med. 2023 Sep;55(9):2005–24.	esc	Liver organoids	Matrigel	Matrigel
263	36573434	Weng Y, Han S, Sekyi MT, Su T, Mattis AN, Chang TT. Self-Assembled Matrigel-Free iPSC-Derived Liver Organoids Demonstrate Wide-Ranging Highly Differentiated Liver Functions. Stem Cells. 2023 Mar 2;41(2):126–39.	ipsc	Liver organoids	Matrigel	Matrigel
264	36484723	Kogler S, Aizenshtadt A, Harrison S, Skottvoll FS, Berg HE, Abadpour S, et al. ‘Organ-in-a-Column’ Coupled On-line with Liquid Chromatography-Mass Spectrometry. Anal Chem. 2022 Dec 20;94(50):17677–84.	ipsc	Liver organoids	Matrigel (GFR)	Matrigel
265	36126647	Jiang S, Xu F, Jin M, Wang Z, Xu X, Zhou Y, et al. Development of a high-throughput micropatterned agarose scaffold for consistent and reproducible hPSC-derived liver organoids. Biofabrication. 2022 Oct 28;15(1).	ipsc	Liver organoids	Vitronectin	Matrigel
266	35688210	Shin J, Toyoda S, Nishitani S, Onodera T, Fukuda S, Kita S, et al. SARS-CoV-2 infection impairs the insulin/IGF signaling pathway in the lung, liver, adipose tissue, and pancreatic cells via IRF1. Metabolism. 2022 Aug;133:155236.	ipsc	Liver organoids	Matrigel	Matrigel
267	34951149	Tao T, Deng P, Wang Y, Zhang X, Guo Y, Chen W, et al. Microengineered Multi-Organoid System from hiPSCs to Recapitulate Human Liver-Islet Axis in Normal and Type 2 Diabetes. Adv Sci (Weinh). 2022 Feb;9(5):e2103495.	ipsc	Liver organoids	Matrigel	Matrigel
268	34665487	Ogoke O, Guiggey D, Mon T, Shamul C, Ross S, Rao S, et al. Spatiotemporal imaging and analysis of mouse and human liver bud morphogenesis. Dev Dyn. 2022 Apr;251(4):662–86.	ipsc	Liver organoids	Matrigel (GFR)	Matrigel

269	34407934	Pettinato G, Coughlan MF, Zhang X, Chen L, Khan U, Glyavina M, et al. Spectroscopic label-free microscopy of changes in live cell chromatin and biochemical composition in transplantable organoids. Sci Adv. 2021 Aug;7(34):eabj2800.	ipsc	Liver organoids	Matrigel	Matrigel
270	34026460	Guo J, Duan L, He X, Li S, Wu Y, Xiang G, et al. A Combined Model of Human iPSC-Derived Liver Organoids and Hepatocytes Reveals Ferroptosis in DGUOK Mutant mtDNA Depletion Syndrome. Adv Sci (Weinh). 2021 May;8(10):2004680.	ipsc	Liver organoids	Matrigel	Matrigel
271	33440728	Mun SJ, Lee J, Chung KS, Son MY, Son MJ. Effect of Microbial Short-Chain Fatty Acids on CYP3A4-Mediated Metabolic Activation of Human Pluripotent Stem Cell-Derived Liver Organoids. Cells. 2021 Jan 11;10(1):126.	ipsc	Liver organoids	Feeder	Matrigel
272	33320545	Wang Y, Wang H, Deng P, Tao T, Liu H, Wu S, et al. Modeling Human Nonalcoholic Fatty Liver Disease (NAFLD) with an Organoids-on-a-Chip System. ACS Biomater Sci Eng. 2020 Oct 12;6(10):5734–43.	ipsc	Liver organoids	Matrigel	Matrigel
273	33319899	Yin F, Zhang X, Wang L, Wang Y, Zhu Y, Li Z, et al. HiPSC-derived multi-organoids-on-chip system for safety assessment of antidepressant drugs. Lab Chip. 2021 Feb 9;21(3):571–81.	ipsc	Liver organoids	Matrigel	Matrigel
274	32579880	Yang L, Han Y, Nilsson-Payant BE, Gupta V, Wang P, Duan X, et al. A Human Pluripotent Stem Cell-based Platform to Study SARS-CoV-2 Tropism and Model Virus Infection in Human Cells and Organoids. Cell Stem Cell. 2020 Jul 2;27(1):125-136.e7.	ipsc	Liver organoids	Matrigel	Matrigel
275	39771560	Ryu JH, Yu J, Jeon JS, Jo S, Lee SM, Kim H, et al. Heterotropic Activation of Cytochrome P450 3A4 by Perillyl Alcohol. Pharmaceutics. 2024 Dec 11;16(12):1581.	ipsc	Liver organoids	Vitronectin	Matrigel
276	37856222	Hidalgo-Álvarez J, Salas-Lucia F, Vera Cruz D, Fonseca TL, Bianco AC. Localized T3 production modifies the transcriptome and promotes the hepatocyte-like lineage in iPSC-derived hepatic organoids. JCI Insight. 2023 Dec 8;8(23):e173780.	ipsc	Liver organoids	Matrigel	Matrigel
277	37552975	Zhang XS, Xie G, Ma H, Ding S, Wu YX, Fei Y, et al. Highly reproducible and cost-effective one-pot organoid differentiation using a novel platform based on PF-127 triggered spheroid assembly. Biofabrication. 2023 Aug 21;15(4).	esc	Liver organoids	Matrigel	Matrigel
278	36927149	Westhaus A, Cabanes-Creus M, Dilworth KL, Zhu E, Salas Gómez D, Navarro RG, et al. Assessment of Pre-Clinical Liver Models Based on Their Ability to Predict the Liver-Tropism of Adeno-Associated Virus Vectors. Hum Gene Ther. 2023 Apr;34(7–8):273–88.	ipsc	Liver organoids	Matrigel	Matrigel
279	35598335	Kim H, Im I, Jeon JS, Kang EH, Lee HA, Jo S, et al. Development of human pluripotent stem cell-derived hepatic organoids as an alternative model for drug safety assessment. Biomaterials. 2022 Jul;286:121575.	ipsc, esc	Liver organoids	Vitronectin	Matrigel
280	31760071	Guan Y, Chen X, Wu M, Zhu W, Arslan A, Takeda S, et al. The phosphatidylethanolamine biosynthesis pathway provides a new target for cancer chemotherapy. J Hepatol. 2020 Apr;72(4):746–60.	ipsc	Liver organoids	Matrigel	Matrigel
281	35883684	Nam D, Park MR, Lee H, Bae SC, Gerovska D, Araúzo-Bravo MJ, et al. Induced Endothelial Cell-Integrated Liver Assembloids Promote Hepatic Maturation and Therapeutic Effect on Cholestatic Liver Fibrosis. Cells. 2022 Jul 19;11(14):2242.	ipsc	Liver organoids	Feeder	Matrigel + Collagen-1
282	35723330	Chung PHY, Babu RO, Wu Z, Wong KKY, Tam PKH, Lui VCH. Developing Biliary Atresia-like Model by Treating Human Liver Organoids with Polyinosinic:Polycytidylic Acid (Poly (I:C)). Curr Issues Mol Biol. 2022 Jan 27;44(2):644–53.	ipsc	Liver organoids	Feeder	Matrigel
