# Supplementary material for: Biopolymers for Liver Tissue Engineering: A Systematic Review
Source: Gels. 2025 Jul 7;11(7):525. doi: 10.3390/gels11070525 (PMC12294766; doi:10.3390/gels11070525)
Supplement: Supplementary file 1 [file gels-11-00525-s001.zip › Table S2.pdf]

Table S2: Studies using biopolymers to bioengineer human liver from LSCs

S/N	PMID or DOI	Article	End target	Maintenance Substrate	Differentiation substrate
1	35226290	Di Matteo S, Di Meo C, Carpino G, Zoratto N, Cardinale V, Nevi L, et al. Therapeutic effects of dexamethasone-loaded hyaluronan nanogels in the experimental cholestasis. Drug Deliv Transl Res. 2022 Aug;12(8):1959–73.	Biliary Organoids	Hyaluronic Acid	Hyaluronic Acid
2	35344800	Willemse J, van Tienderen G, van Hengel E, Schurink I, van der Ven D, Kan Y, et al. Hydrogels derived from decellularized liver tissue support the growth and differentiation of cholangiocyte organoids. Biomaterials. 2022 May;284:121473.	Biliary Organoids	Matrigel	Liver ECM
3	35685290	Zarei K, Thornell IM, Stoltz DA. Anion Transport Across Human Gallbladder Organoids and Monolayers. Front Physiol. 2022;13:882525.	Biliary Organoids	Matrigel	Matrigel
4	10.1016/j.jhep.2021.03.029	Reich M, Spomer L, Klindt C, Fuchs K, Stindt J, Deutschmann K, et al. Downregulation of TGR5 (GPBAR1) in biliary epithelial cells contributes to the pathogenesis of sclerosing cholangitis. J Hepatol. 2021 Sep;75(3):634–46.	Biliary Organoids	Matrigel	Matrigel
5	39209139	Pinto TS, van der Eerden BC, Schreuders-Koedam M, van de Peppel J, Ayada I, Pan Q, et al. Interaction of high lipogenic states with titanium on osteogenesis. Bone. 2024 Nov;188:117242.	Biliary Organoids	Matrigel	Matrigel
6	39117112	Yao Q, Wang B, Yu J, Pan Q, Yu Y, Feng X, et al. ROS-responsive nanoparticle delivery of obeticholic acid mitigate primary sclerosing cholangitis. J Control Release. 2024 Oct;374:112–26.	Biliary Organoids	Matrigel	Matrigel
7	36382606	Nguyen VVT, Ye S, Gkouzioti V, van Wolferen ME, Yengej FY, Melkert D, et al. A human kidney and liver organoid-based multi-organ-on-a-chip model to study the therapeutic effects and biodistribution of mesenchymal stromal cell-derived extracellular vesicles. J Extracell Vesicles. 2022 Nov;11(11):e12280.	Biliary Organoids	Matrigel	Matrigel
8	35803261	Narayan NJC, Requena D, Lalazar G, Ramos-Espiritu L, Ng D, Levin S, et al. Human liver organoids for disease modeling of fibrolamellar carcinoma. Stem Cell Reports. 2022 Aug 9;17(8):1874–88.	Biliary Organoids	Matrigel	Matrigel
9	39878507	Wu H, Yang ASP, Stelloo S, Roos FJM, Te Morsche RHM, Verkerk AH, et al. Multi-omics analysis reveals distinct gene regulatory mechanisms between primary and organoid-derived human hepatocytes. Dis Model Mech. 2025 Jan 1;18(1):dmm050883.	Biliary Organoids	Matrigel	Matrigel
10	38678809	Xiao MH, Wu S, Liang P, Ma D, Zhang J, Chen H, et al. Mucosal-associated invariant T cells promote ductular reaction through amphiregulin in biliary atresia. EBioMedicine. 2024 May;103:105138.	Biliary Organoids	Matrigel (GFR)	Matrigel
11	38522506	Zhao H, Tian X, Wu B, Lu Y, Du J, Peng S, et al. Neurotensin contributes to cholestatic liver disease potentially modulating matrix metalloprotease-7. Int J Biochem Cell Biol. 2024 May;170:106567.	Biliary Organoids	Matrigel	Matrigel
12	37820623	Du Y, de Jong IEM, Gupta K, Waisbourd-Zinman O, Har-Zahav A, Soroka CJ, et al. Human vascularized bile duct-on-a chip: a multi-cellular micro-physiological system for studying cholestatic liver disease. Biofabrication. 2023 Oct 20;16(1):015004.	Biliary Organoids	Matrigel	Matrigel
13	36497057	Aktas RG, Karski M, Issac B, Sun L, Rockowitz S, Sliz P, et al. Long-Term Characteristics of Human-Derived Biliary Organoids under a Single Continuous Culture Condition. Cells. 2022 Nov 27;11(23):3797.	Biliary Organoids	Matrigel	Matrigel
14	32843549	Chen S, Li P, Wang Y, Yin Y, de Ruiter PE, Verstegen MMA, et al. Rotavirus Infection and Cytopathogenesis in Human Biliary Organoids Potentially Recapitulate Biliary Atresia Development. mBio. 2020 Aug 25;11(4):e01968-20.	Biliary Organoids	Matrigel	Matrigel
15	39160386	Frank AK, Chung BK, De Novales MLL, Engesæter LK, Hoyle HW, Øgaard J, et al. Single-Cell Transcriptomic Profiling of Cholangiocyte Organoids Derived from Bile Ducts of Primary Sclerosing Cholangitis Patients. Dig Dis Sci. 2024 Oct;69(10):3810–23.	Biliary Organoids	Matrigel	Matrigel
16	38829197	Garcia Moreno AS, Guicciardi ME, Wixom AQ, Jessen E, Yang J, Ilyas SI, et al. IL-17 signaling in primary sclerosing cholangitis patient-derived organoids. Hepatol Commun. 2024 Jun 1;8(6):e0454.	Biliary Organoids	Matrigel	Matrigel
17	38475870	Wang Z, Xing C, van der Laan LJW, Verstegen MMA, Spee B, Masereeuw R. Cholangiocyte organoids to study drug-induced injury. Stem Cell Res Ther. 2024 Mar 13;15(1):78.	Biliary Organoids	Matrigel	Matrigel
18	38407207	Islam D, Israr I, Taleb MAB, Rao A, Yosief R, Sultana R, et al. A novel model to study mechanisms of cholestasis in human cholangiocytes reveals a role for the SIPR2 pathway. Hepatol Commun. 2024 Mar 1;8(3):e0389.	Biliary Organoids	Matrigel	Matrigel
19	38369341	Mizoi K, Okada R, Mashimo A, Masuda N, Itoh M, Ishida S, et al. Novel Screening System for Biliary Excretion of Drugs Using Human Cholangiocyte Organoid Monolayers with Directional Drug Transport. Biol Pharm Bull. 2024;47(2):427–33.	Biliary Organoids	Matrigel	Matrigel
20	36677681	Bouwmeester MC, Tao Y, Proença S, van Steenbeek FG, Samsom RA, Nijmeijer SM, et al. Drug Metabolism of Hepatocyte-like Organoids and Their Applicability in In Vitro Toxicity Testing. Molecules. 2023 Jan 7;28(2):621.	Biliary Organoids	Matrigel	Matrigel
21	36429040	Koch M, Nickel S, Lieshout R, Lissek SM, Leskova M, van der Laan LJW, et al. Label-Free Imaging Analysis of Patient-Derived Cholangiocarcinoma Organoids after Sorafenib Treatment. Cells. 2022 Nov 15;11(22):3613.	Biliary Organoids	Matrigel	Matrigel
22	35764936	Lieshout R, Faria AVS, Peppelenbosch MP, van der Laan LJW, Verstegen MMA, Fuhler GM. Kinome profiling of cholangiocarcinoma organoids reveals potential druggable targets that hold promise for treatment stratification. Mol Med. 2022 Jun 28;28(1):74.	Biliary Organoids	Matrigel	Matrigel
23	35724847	de Bruijn VMP, Wang Z, Bakker W, Zheng W, Spee B, Bouwmeester H. Hepatic bile acid synthesis and secretion: Comparison of in vitro methods. Toxicol Lett. 2022 Jul 15;365:46–60.	Biliary Organoids	Matrigel	Matrigel
24	35523140	Roos FJM, van Tienderen GS, Wu H, Bordeu I, Vinke D, Albarinos LM, et al. Human branching cholangiocyte organoids recapitulate functional bile duct formation. Cell Stem Cell. 2022 May 5;29(5):776-794.e13.	Biliary Organoids	Matrigel	Matrigel
25	34954911	Roos FJM, Wu H, Willemse J, Lieshout R, Albarinos LAM, Kan YY, et al. Cholangiocyte organoids from human bile retain a local phenotype and can repopulate bile ducts in vitro. Clin Transl Med. 2021 Dec;11(12):e566.	Biliary Organoids	Matrigel	Matrigel
26	34922851	Bijvelds MJC, Roos FJM, Meijsen KF, Roest HP, Verstegen MMA, Janssens HM, et al. Rescue of chloride and bicarbonate transport by elexacaftor-ivacaftor-tezacaftor in organoid-derived CF intestinal and cholangiocyte monolayers. J Cyst Fibros. 2022 May;21(3):537–43.	Biliary Organoids	Matrigel	Matrigel
27	34795391	Nguyen L, Jager M, Lieshout R, de Ruiter PE, Locati MD, Besselink N, et al. Precancerous liver diseases do not cause increased mutagenesis in liver stem cells. Commun Biol. 2021 Nov 18;4(1):1301.	Biliary Organoids	Matrigel	Matrigel

28	34700031	Shi S, Verstegen MMA, Roest HP, Ardisasmita AI, Cao W, Roos FJM, et al. Recapitulating Cholangiopathy-Associated Necroptotic Cell Death In Vitro Using Human Cholangiocyte Organoids. Cell Mol Gastroenterol Hepatol. 2022;13(2):541–64.	Biliary Organoids	Matrigel	Matrigel
29	34671987	Lehmann V, Schene IF, Ardisasmita AI, Liv N, Veenendaal T, Klumperman J, et al. The potential and limitations of intrahepatic cholangiocyte organoids to study inborn errors of metabolism. J Inherit Metab Dis. 2022 Mar;45(2):353–65.	Biliary Organoids	Matrigel	Matrigel
30	33655768	Roos FJM, Bijvelds MJC, Verstegen MMA, Roest HP, Metselaar HJ, Polak WG, et al. Impact of hypoxia and AMPK on CFTR-mediated bicarbonate secretion in human cholangiocyte organoids. Am J Physiol Gastrointest Liver Physiol. 2021 May 1;320(5):G741–52.	Biliary Organoids	Matrigel	Matrigel
31	33634107	Roos FJM, Verstegen MMA, Muñoz Albarinos L, Roest HP, Poley JW, Tetteroo GWM, et al. Human Bile Contains Cholangiocyte Organoid-Initiating Cells Which Expand as Functional Cholangiocytes in Non-canonical Wnt Stimulating Conditions. Front Cell Dev Biol. 2020;8:630492.	Biliary Organoids	Matrigel	Matrigel
32	33602855	Sampaziotis F, Muraro D, Tysoe OC, Sawiak S, Beach TE, Godfrey EM, et al. Cholangiocyte organoids can repair bile ducts after transplantation in the human liver. Science. 2021 Feb 19;371(6531):839–46.	Biliary Organoids	Matrigel	Matrigel
33	33318612	Verstegen MMA, Roos FJM, Burka K, Gehart H, Jager M, de Wolf M, et al. Human extrahepatic and intrahepatic cholangiocyte organoids show region-specific differentiation potential and model cystic fibrosis-related bile duct disease. Sci Rep. 2020 Dec 14;10(1):21900.	Biliary Organoids	Matrigel	Matrigel
34	36241695	Ardisasmita AI, Schene IF, Joore IP, Kok G, Hendriks D, Artegiani B, et al. A comprehensive transcriptomic comparison of hepatocyte model systems improves selection of models for experimental use. Commun Biol. 2022 Oct 14;5(1):1094.	Biliary Organoids, Liver Organoids	Matrigel	Matrigel
35	36972396	Fiorotto R, Mariotti V, Taleb SA, Zehra SA, Nguyen M, Amenduni M, et al. Cell-matrix interactions control biliary organoid polarity, architecture, and differentiation. Hepatol Commun. 2023 Apr 1;7(4):e0094.	Biliary Organoids	Matrigel (GFR)	Matrigel (GFR)
36	34392560	Amarachintha SP, Mourya R, Ayabe H, Yang L, Luo Z, Li X, et al. Biliary organoids uncover delayed epithelial development and barrier function in biliary atresia. Hepatology. 2022 Jan;75(1):89–103.	Biliary Organoids	Matrigel (GFR)	Matrigel (GFR)
37	32222998	Rimland CA, Tilson SG, Morell CM, Tomaz RA, Lu WY, Adams SE, et al. Regional Differences in Human Biliary Tissues and Corresponding In Vitro-Derived Organoids. Hepatology. 2021 Jan;73(1):247–67.	Biliary Organoids	Matrigel (GFR)	Matrigel (GFR)
38	39323909	Kreiner P, Eggenhofer E, Schneider L, Rejas C, Goetz M, Bogovic N, et al. Extrahepatic Bile Duct Organoids as a Model to Study Ischemia/Reperfusion Injury During Liver Transplantation. Transpl Int. 2024;37:13212.	Biliary Organoids	Matrigel (GFR)	Matrigel (GFR)
39	38009279	Lim CK, Romeo O, Tran BM, Flanagan DJ, Kirby EN, McCartney EM, et al. Assessment of hepatitis B virus infection and interhost cellular responses using intrahepatic cholangiocyte organoids. J Med Virol. 2023 Nov;95(11):e29232.	Biliary Organoids	Matrigel (GFR)	Matrigel (GFR)
40	37788895	Zecher BF, Ellinghaus D, Schloer S, Niehrs A, Padoan B, Baumdick ME, et al. HLA-DPA1*02:01~B1*01:01 is a risk haplotype for primary sclerosing cholangitis mediating activation of NKp44+ NK cells. Gut. 2024 Jan 5;73(2):325–37.	Biliary Organoids	Matrigel (GFR)	Matrigel (GFR)
41	36608526	Shi S, Roest HP, van den Bosch TPP, Bijvelds MJC, Boehnert MU, de Jonge J, et al. Modeling bile duct ischemia and reoxygenation injury in human cholangiocyte organoids for screening of novel cholangio-protective agents. EBioMedicine. 2023 Feb;88:104431.	Biliary Organoids	Matrigel (GFR)	Matrigel (GFR)
42	36429084	van Tienderen GS, Willemse J, van Loo B, van Hengel EVA, de Jonge J, van der Laan LJW, et al. Scalable Production of Size-Controlled Cholangiocyte and Cholangiocarcinoma Organoids within Liver Extracellular Matrix-Containing Microcapsules. Cells. 2022 Nov 18;11(22):3657.	Biliary Organoids	Matrigel (GFR)	Matrigel (GFR)
43	36109670	Wesley BT, Ross ADB, Muraro D, Miao Z, Saxton S, Tomaz RA, et al. Single-cell atlas of human liver development reveals pathways directing hepatic cell fates. Nat Cell Biol. 2022 Oct;24(10):1487–98.	Biliary Organoids, Liver Organoids	Matrigel (GFR)	Matrigel (GFR)
44	35813994	Wang Z, Faria J, van der Laan LJW, Penning LC, Masereeuw R, Spee B. Human Cholangiocytes Form a Polarized and Functional Bile Duct on Hollow Fiber Membranes. Front Bioeng Biotechnol. 2022;10:868857.	Biliary Organoids	Matrigel	Matrigel + Collagen-1
45	39044566	Wang Z, Ye S, van der Laan LJW, Schneeberger K, Masereeuw R, Spee B. Chemically Defined Organoid Culture System for Cholangiocyte Differentiation. Adv Healthc Mater. 2024 Dec;13(30):e2401511.	Biliary Organoids	Matrigel	Matrigel + Collagen-1, PIC+Collagen-1
46	39183353	Li P, Miyamoto D, Fukumoto M, Kawaguchi Y, Yamashita M, Tetsuo H, et al. Generation of human hepatobiliary organoids with a functional bile duct from chemically induced liver progenitor cells. Stem Cell Res Ther. 2024 Aug 26;15(1):269.	Biliary Organoids	Collagen 1	Suspension Culture
47	37263345	Zhang W, Kyritsi K, Isidan A, Park Y, Li P, Cross-Najafi AA, et al. Development of Scaffold-Free Three-Dimensional Cholangiocyte Organoids to Study the Progression of Primary Sclerosing Cholangitis. Am J Pathol. 2023 Sep;193(9):1156–69.	Biliary Organoids	Collagen-1	Suspension Culture
48	35079629	Buisson EM, Park SH, Kim M, Kang K, Yoon S, Lee JE, et al. Transplantation of patient specific bile duct bioengineered with chemically reprogrammed and microtopographically differentiated cells. Bioeng Transl Med. 2022 Jan;7(1):e10252.	Cholangiocytes, Biliary Organoids	Collagen-1	Gelatin then Matrigel
49	36475903	Ferreira-Gonzalez S, Man TY, Esser H, Aird R, Kilpatrick AM, Rodrigo-Torres D, et al. Senolytic treatment preserves biliary regenerative capacity lost through cellular senescence during cold storage. Sci Transl Med. 2022 Dec 7;14(674):eabj4375.	Cholangiocytes	Collagen-1	Collagen-1
50	33296648	Feng S, Wu J, Qiu WL, Yang L, Deng X, Zhou Y, et al. Large-scale Generation of Functional and Transplantable Hepatocytes and Cholangiocytes from Human Endoderm Stem Cells. Cell Rep. 2020 Dec 8;33(10):108455.	Cholangiocytes, Hepatocytes, Liver Organoids	Matrigel (GFR)	Matrigel (GFR)
51	39524547	Kim M, Kim TH, Salas ESS, Jeon S, Shin JH, Choi D. The efficacy of exosomes from human chemically derived hepatic progenitors in liver damage alleviation: a preclinical experimental study. Ann Surg Treat Res. 2024 Nov;107(5):252–63.	Hepatocytes	Collagen 1	Collagen 1
52	37661098	Kim M, Kim Y, Silva ESS, Adisasmita M, Kim KS, Jung YK, et al. Enhancing generation efficiency of liver organoids in a collagen scaffold using human chemically derived hepatic progenitors. Ann Hepatobiliary Pancreat Surg. 2023 Nov 30;27(4):342–9.	Hepatocytes	Collagen-1	Collagen-1
53	33393114	Prabhakar B, Lee S, Bochanis A, He W, Manautou JE, Rasmussen TP. lnc-RHL, a novel long non-coding RNA required for the differentiation of hepatocytes from human bipotent progenitor cells. Cell Prolif. 2021 Feb;54(2):e12978.	Hepatocytes	Collagen-1	Collagen-1

54	34034028	Kim Y, Kim YW, Lee SB, Kang K, Yoon S, Choi D, et al. Hepatic patch by stacking patient-specific liver progenitor cell sheets formed on multiscale electrospun fibers promotes regenerative therapy for liver injury. Biomaterials. 2021 Jul;274:120899.	Hepatocytes	Collagen-1	Collagen-1
55	38681862	Yap KK, Schröder J, Gerrand YW, Dobric A, Kong AM, Fox AM, et al. Liver specification of human iPSC-derived endothelial cells transplanted into mouse liver. JHEP Rep. 2024 May;6(5):101023.	Hepatocytes	Matrigel	Matrigel
56	39309110	Zhang W, Cui Y, Lu M, Xu M, Li Y, Song H, et al. Hormonally and chemically defined expansion conditions for organoids of biliary tree Stem Cells. Bioact Mater. 2024 Nov;41:672–95.	Hepatocytes	Matrigel	Matrigel
57	39604571	Chen Y, Wu Y, Sun H, Zhang H, Tang D, Yuan T, et al. Human liver progenitor-like cells-derived extracellular vesicles promote liver regeneration during acute liver failure. Cell Biol Toxicol. 2024 Nov 28;40(1):106.	Hepatocytes	Matrigel	Matrigel
58	34601385	Li B, Wang Y, Pelz C, Moss J, Shemer R, Dor Y, et al. In vitro expansion of cirrhosis derived liver epithelial cells with defined small molecules. Stem Cell Res. 2021 Oct;56:102523.	Hepatocytes	Collagen-1	Matrigel
59	39527612	Asano R, Iizaka Y, Kashima M, Anzai Y, Yamaguchi S, Tada M. Unveiling dynamic hepatocyte plasticity in HepaRG cells with a dual CYP reporter system. PLoS One. 2024;19(11):e0308694.	Hepatocytes	No coating	No coating
60	39127924	De Berdt P, Deltour E, Pauly E, Gordillo N, Lin F, Sokal E, et al. Expansion of human allogeneic liver-derived progenitor cells for liver regenerative therapy in serum-free culture conditions. Cytotherapy. 2024 Dec;26(12):1571–8.	Hepatocytes	No coating	No coating
61	38389704	Tapparo M, Saccu G, Pasquino C, Fonsato V, Medana C, Schiavo V, et al. In vitro characterization of 3D culture-based differentiation of human liver stem cells. Front Cell Dev Biol. 2024;12:1352013.	Hepatocytes	No coating	No coating
62	36768260	Bellanti F, Mangieri D, di Bello G, Lo Buglio A, Pannone G, Pedicillo MC, et al. Redox-Dependent Modulation of Human Liver Progenitor Cell Line Fate. Int J Mol Sci. 2023 Jan 18;24(3):1934.	Hepatocytes	No coating	No coating
63	39844884	Janiga-MacNelly A, Vrazel M, Roat AE, Fernandez-Luna MT, Lavado R. Exploring the biological impact of bacteria-derived indole compounds on human cell health: Cytotoxicity and cell proliferation across six cell lines. Toxicol Rep. 2025 Jun;14:101883.	Hepatocytes	No coating	No coating
64	39627601	Costamagna A, Pasquino C, Lamorte S, Navarro-Tableros V, Delsedime L, Fanelli V, et al. Human liver stem cells and derived extracellular vesicles protect from sepsis-induced acute lung injury and restore bone marrow myelopoiesis in a murine model of sepsis. Intensive Care Med Exp. 2024 Dec 3;12(1):111.	Hepatocytes	No coating	No coating
65	39062090	Ceccotti E, Chiabotto G, Cedrino M, Gambella A, Delsedime L, Ghigo A, et al. Extracellular Vesicles Derived from Human Liver Stem Cells Counteract Chronic Kidney Disease Development and Cardiac Dysfunction in Remnant Kidney Murine Model: The Possible Involvement of Proteases. Biomedicines. 2024 Jul 8;12(7):1517.	Hepatocytes	No coating	No coating
66	36576512	Capinha L, Zhang Y, Holzer AK, Ückert AK, Zana M, Carta G, et al. Transcriptomic-based evaluation of trichloroethylene glutathione and cysteine conjugates demonstrate phenotype-dependent stress responses in a panel of human in vitro models. Arch Toxicol. 2023 Feb;97(2):523–45.	Hepatocytes	No coating	No coating
67	34786160	Bellanti F, di Bello G, Tamborra R, Amatruda M, Lo Buglio A, Dobrakowski M, et al. Impact of senescence on the transdifferentiation process of human hepatic progenitor-like cells. World J Stem Cells. 2021 Oct 26;13(10):1595–609.	Hepatocytes	No coating	No coating
68	33928152	Zeng J, Jing Y, Wu Q, Zeng J, Wei L, Liu J. Autophagy Is Required for Hepatic Differentiation of Hepatic Progenitor Cells via Wnt Signaling Pathway. Biomed Res Int. 2021;2021:6627506.	Hepatocytes	No coating	No coating
69	33495943	Yamazaki T, Tokiwa T. Elevated levels of expression of cytochrome P450 3A4 in a human liver epithelial cell line in differentiation-inducing conditions. Hum Cell. 2021 May;34(3):750–8.	Hepatocytes	No coating	No coating
70	37660999	Joshi P, Kang SY, Acharya P, Sidhpura D, Lee MY. High-throughput assessment of metabolism-mediated neurotoxicity by combining 3D-cultured neural stem cells and liver cell spheroids. Toxicol In Vitro. 2023 Dec;93:105688.	Hepatocytes	Suspension Culture	Suspension Culture
71	39280896	Wei H, Xue T, Li F, Ju E, Wang H, Li M, et al. Framework nucleic Acid-MicroRNA mediated hepatic differentiation and functional hepatic spheroid development for treating acute liver failure. Bioact Mater. 2024 Nov;41:611–26.	Hepatocytes	No coating	Suspension Culture
72	39061774	Thorbow J, Strauch A, Pfening V, Klee JP, Brücher P, Boshof B, et al. Large-Scale Expansion of Human Liver Stem Cells Using Two Different Bioreactor Systems. Bioengineering (Basel). 2024 Jul 9;11(7):692.	Hepatocytes	No coating	Suspension Culture
73	38725803	Adisasmita M, Lee HK, An Y, Kim M, Mamo MG, Hur JK, et al. Epigenetic modulation inhibits epithelial-mesenchymal transition-driven fibrogenesis and enhances characteristics of chemically-derived hepatic progenitors. Ann Surg Treat Res. 2024 May;106(5):274–83.	Liver Organoids	Collagen 1	Collagen 1
74	36738840	Zhang CJ, Meyer SR, O'Meara MJ, Huang S, Capeling MM, Ferrer-Torres D, et al. A human liver organoid screening platform for DILI risk prediction. J Hepatol. 2023 May;78(5):998–1006.	Liver Organoids	Collagen-1 + Fibronectin	Collagen-1
75	34589593	Brovold M, Keller D, Devarasetty M, Dominijanni A, Shirwaiker R, Soker S. Biofabricated 3D in vitro model of fibrosis-induced abnormal hepatoblast/biliary progenitors' expansion of the developing liver. Bioeng Transl Med. 2021 Sep;6(3):e10207.	Liver Organoids	No coating	Collagen-1
76	38508126	Li Y, Xu C, Zhou X, Li J, Xu S, Tu Y, et al. DNA adductomics aided rapid screening of genotoxic impurities using nucleosides and 3D bioprinted human liver organoids. Talanta. 2024 Jun 1;273:125902.	Liver Organoids	No coating	Gelatin + Alginate
77	37569847	Pérez-Luz S, Lalchandani J, Matamala N, Barrero MJ, Gil-Martín S, Saz SRD, et al. Quantitative Lipid Profiling Reveals Major Differences between Liver Organoids with Normal Pi*M and Deficient Pi*Z Variants of Alpha-1-antitrypsin. Int J Mol Sci. 2023 Aug 5;24(15):12472.	Liver Organoids	Matrigel (GFR)	GFR Matrigel
78	38736008	Tong Y, Ueyama-Toba Y, Yokota J, Matsui H, Kanai M, Mizuguchi H. Efficient hepatocyte differentiation of primary human hepatocyte-derived organoids using three dimensional nanofibers (HYDROX) and their possible application in hepatotoxicity research. Sci Rep. 2024 May 13;14(1):10846.	Liver Organoids	Matrigel (GFR)	Hydrox
79	40117762	Myszczyszyn A, Muench A, Lehmann V, Sinnige T, van Steenbeek FG, Bouwmeester M, et al. A hollow fiber membrane-based liver organoid-on-a-chip model for examining drug metabolism and transport. Biofabrication. 2025 Apr 1;17(2).	Liver Organoids	Matrigel (GFR)	Laminin 332
80	39699962	Defamie V, Aliar K, Sarkar S, Vyas F, Shetty R, Reddy Narala S, et al. Metalloproteinase inhibitors regulate biliary progenitor cells through sDLK1 in organoid models of liver injury. J Clin Invest. 2024 Dec 19;135(3):e164997.	Liver Organoids	Matrigel	Matrigel

81	38232180	Caipa Garcia AL, Kucab JE, Al-Serori H, Beck RSS, Bellamri M, Turesky RJ, et al. Tissue Organoid Cultures Metabolize Dietary Carcinogens Proficiently and Are Effective Models for DNA Adduct Formation. Chem Res Toxicol. 2024 Feb 19;37(2):234–47.	Liver Organoids	Matrigel	Matrigel
82	38740814	Hendriks D, Artegiani B, Margaritis T, Zoutendijk I, Chuva de Sousa Lopes S, Clevers H. Mapping of mitogen and metabolic sensitivity in organoids defines requirements for human hepatocyte growth. Nat Commun. 2024 May 13;15(1):4034.	Liver Organoids	Matrigel	Matrigel
83	39834100	Zhao X, Wang S, Liu Q, Wei W, Sun X, Song H, et al. Single-cell landscape of the intrahepatic ecosystem in alcohol-related liver disease. Clin Transl Med. 2025 Jan;15(1):e70198.	Liver Organoids	Matrigel	Matrigel
84	36714539	Tong Y, Ueyama-Toba Y, Mizuguchi H. Biliary epithelial cell differentiation of bipotent human liver-derived organoids by 2D and 3D culture. Biochem Biophys Rep. 2023 Mar;33:101432.	Liver Organoids	Matrigel	Matrigel
85	37137901	Rüland L, Andreatta F, Massalini S, Chuva de Sousa Lopes S, Clevers H, Hendriks D, et al. Organoid models of fibrolamellar carcinoma mutations reveal hepatocyte transdifferentiation through cooperative BAP1 and PRKAR2A loss. Nat Commun. 2023 May 3;14(1):2377.	Liver Organoids	Matrigel	Matrigel
86	35044825	Li P, Li Y, Wang Y, Liu J, Lavrijsen M, Li Y, et al. Recapitulating hepatitis E virus-host interactions and facilitating antiviral drug discovery in human liver-derived organoids. Sci Adv. 2022 Jan 21;8(3):eabj5908.	Liver Organoids	Matrigel	Matrigel
87	33571486	Boonekamp KE, Heo I, Artegiani B, Asra P, van Son G, de Ligt J, et al. Identification of novel human Wnt target genes using adult endodermal tissue-derived organoids. Dev Biol. 2021 Jun;474:37–47.	Liver Organoids	Matrigel	Matrigel
88	34105295	Zhao Y, Li ZX, Zhu YJ, Fu J, Zhao XF, Zhang YN, et al. Single-Cell Transcriptome Analysis Uncovers Intratumoral Heterogeneity and Underlying Mechanisms for Drug Resistance in Hepatobiliary Tumor Organoids. Adv Sci (Weinh). 2021 Jun;8(11):e2003897.	Liver Organoids	Matrigel	Matrigel
89	10.1016/j.dld.2023.01.026	De Siervi S, Nibali SC, Mantovani S, Oliviero B, Mondelli MU, Di Pasqua LG, et al. Patient-derived liver organoids as an in vitro model to study new personalized therapies targeting VDAC1 in intrahepatic cholangiocarcinoma. Digestive and Liver Disease. 2023 Mar 1;55:S14.	Liver Organoids	Matrigel	Matrigel
90	39986119	Gong Y, You Q, Yuan X, Zeng F, Zhang F, Xiao J, et al. Mesenchymal stem cell-derived extracellular vesicles attenuate ferroptosis in aged hepatic ischemia/reperfusion injury by transferring miR-1275. Redox Biol. 2025 Apr;81:103556.	Liver Organoids	Matrigel	Matrigel
91	39872219	Goswami Y, Baghel A, Sharma G, Sharma PK, Biswas S, Yadav R, et al. Liver Organoids From Hepatocytes of Healthy Humans and Non-alcoholic Fatty Liver Disease (NAFLD) Patients Display Multilineage Architecture and can be Used to Develop an In Vitro Model of Steatohepatitis. J Clin Exp Hepatol. 2025;15(3):102463.	Liver Organoids	Matrigel	Matrigel
92	38778114	Gribben C, Galanakis V, Calderwood A, Williams EC, Chazarra-Gil R, Larraz M, et al. Acquisition of epithelial plasticity in human chronic liver disease. Nature. 2024 Jun;630(8015):166–73.	Liver Organoids	Matrigel	Matrigel
93	37938000	Meyers NL, Ashuach T, Lyons DE, Khalid MM, Simoneau CR, Erickson AL, et al. Hepatitis C virus infects and perturbs liver stem cells. mBio. 2023 Dec 19;14(6):e0131823.	Liver Organoids	Matrigel	Matrigel
94	37798809	Kong D, Mourtzinos A, Heegsma J, Blokzijl H, de Meijer VE, Faber KN. Growth differentiation factor 7 autocrine signaling promotes hepatic progenitor cell expansion in liver fibrosis. Stem Cell Res Ther. 2023 Oct 5;14(1):288.	Liver Organoids	Matrigel	Matrigel
95	39561715	Ye S, Marsee A, van Tienderen GS, Rezaeimoghaddam M, Sheikh H, Samsom RA, et al. Accelerated production of human epithelial organoids in a miniaturized spinning bioreactor. Cell Rep Methods. 2024 Nov 18;4(11):100903.	Liver Organoids	Matrigel	Matrigel
96	39329726	Romaldini A, Spanò R, Veronesi M, Grimaldi B, Bandiera T, Sabella S. Human Multi-Lineage Liver Organoid Model Reveals Impairment of CYP3A4 Expression upon Repeated Exposure to Graphene Oxide. Cells. 2024 Sep 13;13(18):1542.	Liver Organoids	Collagen-1	Matrigel
97	39280628	Ueyama-Toba Y, Tong Y, Yokota J, Murai K, Hikita H, Eguchi H, et al. Development of a hepatic differentiation method in 2D culture from primary human hepatocyte-derived organoids for pharmaceutical research. iScience. 2024 Sep 20;27(9):110778.	Liver Organoids	Matrigel	Matrigel
98	39271308	Wai AWY, Lui VCH, Tang CSM, Wang B, Tam PKH, Wong KKY, et al. Human Liver Organoids to Predict the Outcome of Kasai Portoenterostomy. J Pediatr Surg. 2025 Feb;60(2):161686.	Liver Organoids	Matrigel	Matrigel
99	39206088	Byeon JH, Jung DJ, Han HJ, Son WC, Jeong GS. Fast formation and maturation enhancement of human liver organoids using a liver-organoid-on-a-chip. Front Cell Dev Biol. 2024;12:1452485.	Liver Organoids	Matrigel (GFR)	Matrigel
100	39181211	Liu YJ, Kimura M, Li X, Sulc J, Wang Q, Rodríguez-López S, et al. ACMSD inhibition corrects fibrosis, inflammation, and DNA damage in MASLD/MASH. J Hepatol. 2025 Feb;82(2):174–88.	Liver Organoids	Matrigel	Matrigel
101	38770342	Rao S, Romal S, Torenvliet B, Slotman JA, Huijs T, Mahmoudi T. A 3D organoid platform that supports liver-stage P.falciparum infection can be used to identify intrahepatic antimalarial drugs. Heliyon. 2024 May 30;10(10):e30740.	Liver Organoids	Matrigel	Matrigel
102	38660778	Shrestha S, Lekkala VKR, Acharya P, Kang SY, Vanga MG, Lee MY. Reproducible generation of human liver organoids (HLOs) on a pillar plate platform via microarray 3D bioprinting. Lab Chip. 2024 May 14;24(10):2747–61.	Liver Organoids	Matrigel (GFR)	Matrigel
103	38535810	Hai-Bing Y, Sivasankaran MS, Ottakandathil BR, Zhong-Luan W, Man-Ting S, Ho-Yu CP, et al. Environmental Toxin Biliatresone-Induced Biliary Atresia-like Abnormal Cilia and Bile Duct Cell Development of Human Liver Organoids. Toxins (Basel). 2024 Mar 11;16(3):144.	Liver Organoids	Matrigel	Matrigel
104	38400666	Tatsumi K, Wada H, Hasegawa S, Asukai K, Nagata S, Ekawa T, et al. Prediction for oxaliplatin-induced liver injury using patient-derived liver organoids. Cancer Med. 2024 Feb;13(3):e7042.	Liver Organoids	Matrigel	Matrigel
105	37984761	Guo H, Liu D, Liu K, Hou Y, Li C, Li Q, et al. Drug repurposing screen identifies vidofludimus calcium and pyrazofurin as novel chemical entities for the development of hepatitis E interventions. Virol Sin. 2024 Feb;39(1):123–33.	Liver Organoids	Matrigel	Matrigel
106	37962490	Hess A, Gentile SD, Ben Saad A, Rahman RU, Habboub T, Pratt DS, et al. Single-cell transcriptomics stratifies organoid models of metabolic dysfunction-associated steatotic liver disease. EMBO J. 2023 Dec 11;42(24):e113898.	Liver Organoids	Matrigel	Matrigel
107	37947592	Yoon Y, Gong SC, Kim MY, Baik SK, Hong JE, Rhee KJ, et al. Generation of Fibrotic Liver Organoids Using Hepatocytes, Primary Liver Sinusoidal Endothelial Cells, Hepatic Stellate Cells, and Macrophages. Cells. 2023 Oct 24;12(21):2514.	Liver Organoids	Matrigel	Matrigel

108	37749332	Cherubini A, Ostadreza M, Jamialahmadi O, Pelusi S, Rrapaj E, Casirati E, et al. Interaction between estrogen receptor-α and PNPLA3 p.I148M variant drives fatty liver disease susceptibility in women. Nat Med. 2023 Oct;29(10):2643–55.	Liver Organoids	Matrigel (GFR)	Matrigel
109	37253730	Urciuolo A, Giobbe GG, Dong Y, Michielin F, Brandolino L, Magnussen M, et al. Hydrogel-in-hydrogel live bioprinting for guidance and control of organoids and organotypic cultures. Nat Commun. 2023 May 30;14(1):3128.	Liver Organoids	Matrigel	Matrigel
110	37040844	Jin K, Shi Y, Zhang H, Zhangyuan G, Wang F, Li S, et al. A TNFα/Miz1-positive feedback loop inhibits mitophagy in hepatocytes and propagates non-alcoholic steatohepatitis. J Hepatol. 2023 Aug;79(2):403–16.	Liver Organoids	Matrigel	Matrigel
111	36996081	Ye Y, Lui VCH, Babu RO, Wu Z, Wu W, Chung PHY, et al. Identification of cancer-related genes FGFR2 and CEBPB in choledochal cyst via RNA sequencing of patient-derived liver organoids. PLoS One. 2023;18(3):e0283737.	Liver Organoids	Matrigel	Matrigel
112	36737811	Kim HJ, Kim G, Chi KY, Kim H, Jang YJ, Jo S, et al. Generation of multilineage liver organoids with luminal vasculature and bile ducts from human pluripotent stem cells via modulation of Notch signaling. Stem Cell Res Ther. 2023 Feb 3;14(1):19.	Liver Organoids	Matrigel	Matrigel
113	36655405	Busek M, Aizenshtadt A, Koch T, Frank A, Delon L, Martinez MA, et al. Pump-less, recirculating organ-on-a-chip (rOoC) platform. Lab Chip. 2023 Feb 14;23(4):591–608.	Liver Organoids	Matrigel	Matrigel
114	36614051	Caipa Garcia AL, Kucab JE, Al-Serori H, Beck RSS, Fischer F, Hufnagel M, et al. Metabolic Activation of Benzo[a]pyrene by Human Tissue Organoid Cultures. Int J Mol Sci. 2022 Dec 29;24(1):606.	Liver Organoids	Matrigel	Matrigel
115	36435860	Tian X, Wang Y, Lu Y, Wu B, Chen S, Du J, et al. Metabolic regulation of cholestatic liver injury by D-2-hydroxyglutarate with the modulation of hepatic microenvironment and the mammalian target of rapamycin signaling. Cell Death Dis. 2022 Nov 26;13(11):1001.	Liver Organoids	Matrigel	Matrigel
116	36221953	Lam YK, Yu J, Huang H, Ding X, Wong AM, Leung HH, et al. TP53 R249S mutation in hepatic organoids captures the predisposing cancer risk. Hepatology. 2023 Sep 1;78(3):727–40.	Liver Organoids	Matrigel	Matrigel
117	36137184	Tian X, Wang Y, Zhou Y, Wu B, Lu Y, Du J, et al. Beta-amyloid Deposition in Biliary Atresia Reduces Liver Regeneration by Inhibiting Energy Metabolism and Mammalian Target of Rapamycin Signaling. Clin Transl Gastroenterol. 2022 Nov 1;13(11):e00536.	Liver Organoids	Matrigel	Matrigel
118	35704148	Bonanini F, Kurek D, Previdi S, Nicolas A, Hendriks D, de Ruiter S, et al. In vitro grafting of hepatic spheroids and organoids on a microfluidic vascular bed. Angiogenesis. 2022 Nov;25(4):455–70.	Liver Organoids	Matrigel	Matrigel
119	35688027	Tomofuji K, Fukumitsu K, Kondo J, Horie H, Makino K, Wakama S, et al. Liver ductal organoids reconstruct intrahepatic biliary trees in decellularized liver grafts. Biomaterials. 2022 Aug;287:121614.	Liver Organoids	Matrigel	Matrigel
120	35059746	Wang L, Li M, Yu B, Shi S, Liu J, Zhang R, et al. Recapitulating lipid accumulation and related metabolic dysregulation in human liver-derived organoids. J Mol Med (Berl). 2022 Mar;100(3):471–84.	Liver Organoids	Matrigel	Matrigel
121	34929248	Li Y, Li P, He Q, Zhang R, Li Y, Kamar N, et al. Niclosamide inhibits hepatitis E virus through suppression of NF-kappaB signalling. Antiviral Res. 2022 Jan;197:105228.	Liver Organoids	Matrigel	Matrigel
122	34858903	Meng L, Liu J, Wang J, Du M, Zhang S, Huang Y, et al. Characteristics of the Gut Microbiome and IL-13/TGF-β1 Mediated Fibrosis in Post-Kasai Cholangitis of Biliary Atresia. Front Pediatr. 2021;9:751204.	Liver Organoids	Matrigel	Matrigel
123	34838408	Hou C, Sha W, Xu Z, Hu Y, Amakye WK, Yao M, et al. Culture and establishment of self-renewing human liver 3D organoids with high uric acid for screening antihyperuricemic functional compounds. Food Chem. 2022 Apr 16;374:131634.	Liver Organoids	Matrigel	Matrigel
124	34823017	Hou C, Hu Y, Jiang H, Xu Z, Sha W, Liu J, et al. Establishment of a 3D hyperuricemia model based on cultured human liver organoids. Free Radic Biol Med. 2022 Jan;178:7–17.	Liver Organoids	Matrigel	Matrigel
125	34559943	Bouwmeester MC, Bernal PN, Oosterhoff LA, van Wolferen ME, Lehmann V, Vermaas M, et al. Bioprinting of Human Liver-Derived Epithelial Organoids for Toxicity Studies. Macromol Biosci. 2021 Dec;21(12):e2100327.	Liver Organoids	Matrigel	Matrigel
126	33677197	Collett S, Torresi J, Silveira LE, Truong VK, Christiansen D, Tran BM, et al. Investigating virus-host cell interactions: Comparative binding forces between hepatitis C virus-like particles and host cell receptors in 2D and 3D cell culture models. J Colloid Interface Sci. 2021 Jun 15;592:371–84.	Liver Organoids	Matrigel	Matrigel
127	33097693	Schene IF, Joore IP, Oka R, Mokry M, van Vugt AHM, van Boxtel R, et al. Prime editing for functional repair in patient-derived disease models. Nat Commun. 2020 Oct 23;11(1):5352.	Liver Organoids	Matrigel	Matrigel
128	32553668	Babu RO, Lui VCH, Chen Y, Yiu RSW, Ye Y, Niu B, et al. Beta-amyloid deposition around hepatic bile ducts is a novel pathobiological and diagnostic feature of biliary atresia. J Hepatol. 2020 Dec;73(6):1391–403.	Liver Organoids	Matrigel	Matrigel
129	32340283	Lee JY, Han HJ, Lee SJ, Cho EH, Lee HB, Seok JH, et al. Use of 3D Human Liver Organoids to Predict Drug-Induced Phospholipidosis. Int J Mol Sci. 2020 Apr 23;21(8):2982.	Liver Organoids	Matrigel	Matrigel
130	31990370	Claesen JLA, Koomen E, Schene IF, Jans JJM, Mast N, Pikuleva IA, et al. Misdiagnosis of CTX due to propofol: The interference of total intravenous propofol anaesthesia with bile acid profiling. J Inherit Metab Dis. 2020 Jul;43(4):843–51.	Liver Organoids	Matrigel	Matrigel
131	31715015	Schneeberger K, Sánchez-Romero N, Ye S, van Steenbeek FG, Oosterhoff LA, Pla Palacin I, et al. Large-Scale Production of LGR5-Positive Bipotential Human Liver Stem Cells. Hepatology. 2020 Jul;72(1):257–70.	Liver Organoids	Matrigel	Matrigel
132	34943788	Gamboa CM, Wang Y, Xu H, Kalemba K, Wondisford FE, Sabaawy HE. Optimized 3D Culture of Hepatic Cells for Liver Organoid Metabolic Assays. Cells. 2021 Nov 24;10(12):3280.	Liver Organoids	Matrigel	Matrigel
133	37671190	Li P, Miyamoto D, Huang Y, Adachi T, Hidaka M, Hara T, et al. Three-dimensional human bile duct formation from chemically induced human liver progenitor cells. Front Bioeng Biotechnol. 2023;11:1249769.	Liver Organoids	Gelatin	Matrigel (GFR)
134	34631757	Lo Nigro A, Gallo A, Bulati M, Vitale G, Paini DS, Pampalone M, et al. Amnion-Derived Mesenchymal Stromal/Stem Cell Paracrine Signals Potentiate Human Liver Organoid Differentiation: Translational Implications for Liver Regeneration. Front Med (Lausanne). 2021;8:746298.	Liver Organoids	Matrigel (GFR)	Matrigel (GFR)
135	33901295	McCarron S, Bathon B, Conlon DM, Abbey D, Rader DJ, Gawronski K, et al. Functional Characterization of Organoids Derived From Irreversibly Damaged Liver of Patients With NASH. Hepatology. 2021 Oct;74(4):1825–44.	Liver Organoids	Matrigel (GFR)	Matrigel (GFR)
136	39500083	Tong W, Zhu L, Han P, Bai Y, Wang T, Chen D, et al. TWEAK is an activator of Hippo-YAP signaling protecting against hepatic Ischemia/ reperfusion injury. Int Immunopharmacol. 2024 Dec 25;143(Pt 3):113567.	Liver Organoids	Matrigel (GFR)	Matrigel (GFR)

137	38838471	Bitterer F, Kupke P, Adenugba A, Evert K, Glehr G, Riquelme P, et al. Soluble CD46 as a diagnostic marker of hepatic steatosis. EBioMedicine. 2024 Jun;104:105184.	Liver Organoids	Matrigel (GFR)	Matrigel (GFR)
138	38499143	Bonanini F, Singh M, Yang H, Kurek D, Harms AC, Mardinoglu A, et al. A comparison between different human hepatocyte models reveals profound differences in net glucose production, lipid composition and metabolism in vitro. Exp Cell Res. 2024 Apr 1;437(1):114008.	Liver Organoids	Matrigel (GFR)	Matrigel (GFR)
139	36845105	Ziegler AE, Fittje P, Müller LM, Ahrenstorf AE, Hagemann K, Hagen SH, et al. The co-inhibitory receptor TIGIT regulates NK cell function and is upregulated in human intrahepatic CD56bright NK cells. Front Immunol. 2023;14:1117320.	Liver Organoids	Matrigel (GFR)	Matrigel (GFR)
140	35232252	Natarajan V, Simoneau CR, Erickson AL, Meyers NL, Baron JL, Cooper S, et al. Modelling T-cell immunity against hepatitis C virus with liver organoids in a microfluidic coculture system. Open Biol. 2022 Mar;12(3):210320.	Liver Organoids	Matrigel (GFR)	Matrigel (GFR)
141	34328417	De Crignis E, Hossain T, Romal S, Carofiglio F, Moulos P, Khalid MM, et al. Application of human liver organoids as a patient-derived primary model for HBV infection and related hepatocellular carcinoma. Elife. 2021 Jul 30;10:e60747.	Liver Organoids	Matrigel (GFR)	Matrigel (GFR)
142	33730579	Kulsuptrakul J, Wang R, Meyers NL, Ott M, Puschnik AS. A genome-wide CRISPR screen identifies UFMylation and TRAMP-like complexes as host factors required for hepatitis A virus infection. Cell Rep. 2021 Mar 16;34(11):108859.	Liver Organoids	Matrigel (GFR)	Matrigel (GFR)
143	32962010	Saltsman JA, Hammond WJ, Narayan NJC, Requena D, Gehart H, Lalazar G, et al. A Human Organoid Model of Aggressive Hepatoblastoma for Disease Modeling and Drug Testing. Cancers (Basel). 2020 Sep 18;12(9):2668.	Liver Organoids	Matrigel (GFR)	Matrigel (GFR)
144	33021863	Prinelli A, Silva-Almeida C, Parks S, Pasotti A, Telopoulou A, Dunlop S, et al. In-Plate Cryopreservation of 2D and 3D Cell Models: Innovative Tools for Biomedical Research and Preclinical Drug Discovery. SLAS Discov. 2021 Jan;26(1):32–43.	Liver Organoids	Matrigel (GFR)	Matrigel (GFR)
145	38174962	Carpentier N, Ye S, Delemarre MD, Van der Meeren L, Skirtach AG, van der Laan LJW, et al. Gelatin-Based Hybrid Hydrogels as Matrices for Organoid Culture. Biomacromolecules. 2024 Feb 12;25(2):590–604.	Liver Organoids	Matrigel	Matrigel vs. PIC-LECvs. Gelma
146	38465578	Salas-Silva S, Kim Y, Kim TH, Kim M, Seo D, Choi J, et al. Human chemically-derived hepatic progenitors (hCdHs) as a source of liver organoid generation: Application in regenerative medicine, disease modeling, and toxicology testing. Biomaterials. 2023 Dec;303:122360.	Hepatocytes, Liver Organoids	Collagen-1	Matrigel, Collagen-1
147	32651372	Sorrentino G, Rezakhani S, Yildiz E, Nuciforo S, Heim MH, Lutolf MP, et al. Mechano-modulatory synthetic niches for liver organoid derivation. Nat Commun. 2020 Jul 10;11(1):3416.	Liver Organoids	Matrigel	Matrigel, PEG
148	38670107	Isaac R, Bandyopadhyay G, Rohm TV, Kang S, Wang J, Pokhrel N, et al. TM7SF3 controls TEAD1 splicing to prevent MASH-induced liver fibrosis. Cell Metab. 2024 May 7;36(5):1030-1043.e7.	Liver Organoids	No coating	No coating
149	38232700	Sasidharan K, Caddeo A, Jamialahmadi O, Noto FR, Tomasi M, Malvestiti F, et al. IL32 downregulation lowers triglycerides and type I collagen in di-lineage human primary liver organoids. Cell Rep Med. 2024 Jan 16;5(1):101352.	Liver Organoids	No coating	No coating
150	37951556	Bronsard J, Savary C, Massart J, Viel R, Moutaux L, Catheline D, et al. 3D multi-cell-type liver organoids: A new model of non-alcoholic fatty liver disease for drug safety assessments. Toxicol In Vitro. 2024 Feb;94:105728.	Liver Organoids	No coating	No coating
151	36030102	Zhang W, Wauthier E, Lanzoni G, Hani H, Yi X, Overi D, et al. Patch grafting of organoids of stem/progenitors into solid organs can correct genetic-based disease states. Biomaterials. 2022 Sep;288:121647.	Liver Organoids	No coating	No coating
152	35245467	Hallett JM, Ferreira-Gonzalez S, Man TY, Kilpatrick AM, Esser H, Thirlwell K, et al. Human biliary epithelial cells from discarded donor livers rescue bile duct structure and function in a mouse model of biliary disease. Cell Stem Cell. 2022 Mar 3;29(3):355-371.e10.	Liver Organoids	Matrigel	PCL, Matrigel
153	**34658689**	Ye S, Boeter JWB, Mihajlovic M, van Steenbeek FG, van Wolferen ME, Oosterhoff LA, et al. A Chemically Defined Hydrogel for Human Liver Organoid Culture. Adv Funct Mater. 2020 Nov 25;30(48):2000893.	Liver Organoids	Matrigel	PIC+Laminin 511 vs. Matrigel
154	37870288	Oliva-Vilarnau N, Beusch CM, Sabatier P, Sakaraki E, Tjaden A, Graetz L, et al. Wnt/β-catenin and NFκB signaling synergize to trigger growth factor-free regeneration of adult primary human hepatocytes. Hepatology. 2024 Jun 1;79(6):1337–51.	Liver Organoids	Suspension Culture	Suspension Culture
155	34141708	Wang X, Zhang W, Yang Y, Wang J, Qiu H, Liao L, et al. A MicroRNA-Based Network Provides Potential Predictive Signatures and Reveals the Crucial Role of PI3K/AKT Signaling for Hepatic Lineage Maturation. Front Cell Dev Biol. 2021;9:670059.	Liver Organoids	Suspension Culture	Suspension Culture
156	38458193	Yuan X, Wu J, Sun Z, Cen J, Shu Y, Wang C, et al. Preclinical efficacy and safety of encapsulated proliferating human hepatocyte organoids in treating liver failure. Cell Stem Cell. 2024 Apr 4;31(4):484-498.e5.	Liver Organoids	Collagen-1	Suspension Culture
157	37700462	Tao TP, Brandmair K, Gerlach S, Przibilla J, Schepky A, Marx U, et al. Application of a skin and liver Chip2 microphysiological model to investigate the route-dependent toxicokinetics and toxicodynamics of consumer-relevant doses of genistein. J Appl Toxicol. 2024 Feb;44(2):287–300.	Liver Organoids	No coating	Suspension Culture
158	35536601	Kühnlenz J, Karwelat D, Steger-Hartmann T, Raschke M, Bauer S, Vural Ö, et al. A microfluidic thyroid-liver platform to assess chemical safety in humans. ALTEX. 2023;40(1):61–82.	Liver Organoids	No coating	Suspension Culture
