# Supplementary material for: Biopolymers for Liver Tissue Engineering: A Systematic Review
Source: Gels. 2025 Jul 7;11(7):525. doi: 10.3390/gels11070525 (PMC12294766; doi:10.3390/gels11070525)
Supplement: Supplementary file 1 [file gels-11-00525-s001.zip › Table S3.pdf]

Table S3: Studies using biopolymers to bioengineer human liver from NLSCs

S/N	PMID or DOI	Article	End target	Maintenance Substrate	Differentiation substrate	NLSC type
1	32622343	Sun S, Yuan L, An Z, Shi D, Xin J, Jiang J, et al. DLL4 restores damaged liver by enhancing hBMSC differentiation into cholangiocytes. Stem Cell Res. 2020 Jun 29;47:101900.	Cholangiocytes	Collagen-1	Collagen-1	Human bone marrow MSCs
2	37968291	Waki Y, Saito Y, Chen S, Ikemoto T, Noma T, Teraoku H, et al. Effects of green light-emitting diode irradiation on hepatic differentiation of hepatocyte-like cells generated from human adipose-derived mesenchymal cells. Sci Rep. 2023 Nov 15;13(1):19954.	Hepatocytes	Collagen 1	Collagen 1	Human adipose-derived MSCs
3	40175803	Choi MS, Hong JS, Lee DH, Jang YJ, Kim JH, Lee YS. Anti-liver fibrotic effects of small extracellular vesicle microRNAs from human umbilical cord-derived mesenchymal stem cells and their differentiated hepatocyte-like cells. Biotechnol Lett. 2025 Apr 2;47(2):38.	Hepatocytes	Collagen-1	Collagen-1	Human umbilical cord MSCs
4	34215318	Deng J, Luo K, Xu P, Jiang Q, Wang Y, Yao Y, et al. High-efficiency c-Myc-mediated induction of functional hepatoblasts from the human umbilical cord mesenchymal stem cells. Stem Cell Res Ther. 2021 Jul 2;12(1):375.	Hepatocytes	No coating	Collagen-1	Human umbilical cord MSCs
5	37413859	Shibu MA, Huang CY, Ding DC. Comparison of two hepatocyte differentiation protocols in human umbilical cord mesenchymal stem cells: In vitro study. Tissue Cell. 2023 Aug;83:102153.	Hepatocytes	Collagen-1	Collagen-1	Human umbilical cord MSCs
6	32503263	Cipriano M, Pinheiro PF, Sequeira CO, Rodrigues JS, Oliveira NG, Antunes AMM, et al. Nevirapine Biotransformation Insights: An Integrated In Vitro Approach Unveils the Biocompetence and Glutathiolomic Profile of a Human Hepatocyte-Like Cell 3D Model. Int J Mol Sci. 2020 Jun 3;21(11):3998.	Hepatocytes	Collagen-1	Collagen-1	Human umbilical cord MSCs
7	35499693	Rashid S, Salim A, Qazi REM, Malick TS, Haneef K. Sodium Butyrate Induces Hepatic Differentiation of Mesenchymal Stem Cells in 3D Collagen Scaffolds. Appl Biochem Biotechnol. 2022 Aug;194(8):3721–32.	Hepatocytes	No coating	Collagen-1	Human bone marrow MSCs
8	34795766	Gil-Recio C, Montori S, Al Demour S, Ababneh MA, Ferrés-Padró E, Marti C, et al. Chemically Defined Conditions Mediate an Efficient Induction of Dental Pulp Pluripotent-Like Stem Cells into Hepatocyte-Like Cells. Stem Cells Int. 2021;2021:5212852.	Hepatocytes	Fibronectin	Collagen-1	Dental Pulp pluripotent-like stem cells
9	33404674	Merimi M, Lagneaux L, Lombard CA, Agha DM, Bron D, Lewalle P, et al. Immuno-comparative screening of adult-derived human liver stem/progenitor cells for immune-inflammatory-associated molecules. Inflamm Res. 2021 Feb;70(2):229–39.	Hepatocytes	No coating	Collagen-1	Human umbilical cord MSCs
10	32228984	Huang TY, Wang GS, Ko CS, Chen XW, Su WT. A study of the differentiation of stem cells from human exfoliated deciduous teeth on 3D silk fibroin scaffolds using static and dynamic culture paradigms. Mater Sci Eng C Mater Biol Appl. 2020 Apr;109:110563.	Hepatocytes	No coating	Fibroin	Dental MSCs
11	33436050	Yuniartha R, Yamaza T, Sonoda S, Yoshimaru K, Matsuura T, Yamaza H, et al. Cholangiogenic potential of human deciduous pulp stem cell-converted hepatocyte-like cells. Stem Cell Res Ther. 2021 Jan 13;12(1):57.	Hepatocytes	Fibronectin	Fibronectin	Dental MSCs
12	34772451	Choi J, Kang S, Kim B, So S, Han J, Kim GN, et al. Efficient hepatic differentiation and regeneration potential under xeno-free conditions using mass-producible amnion-derived mesenchymal stem cells. Stem Cell Res Ther. 2021 Nov 12;12(1):569.	Hepatocytes	Gelatin	Gelatin	Human placental derived MSCs
13	39075621	Choi J, Kang S, An HI, Kim CE, Lee S, Pack CG, et al. Fasudil and viscosity of gelatin promote hepatic differentiation by regulating organelles in human umbilical cord matrix-mesenchymal stem cells. Stem Cell Res Ther. 2024 Jul 29;15(1):229.	Hepatocytes	Gelatin	Gelatin	Human umbilical cord MSCs
14	34926672	Luo S, Xiao S, Ai Y, Wang B, Wang Y. Changes in the hepatic differentiation potential of human mesenchymal stem cells aged in vitro. Ann Transl Med. 2021 Nov;9(21):1628.	Hepatocytes	Gelatin	Gelatin	Human umbilical cord MSCs
15	34422999	Luo S, Ai Y, Xiao S, Wang B, Wang Y. Functional hit 1 (FH1)-based rapid and efficient generation of functional hepatocytes from human mesenchymal stem cells: a novel strategy for hepatic differentiation. Ann Transl Med. 2021 Jul;9(13):1087.	Hepatocytes	No coating	Gelatin	Human umbilical cord MSCs
16	35883581	Michalik M, Wieczorek P, Czekaj P. In Vitro Differentiation of Human Amniotic Epithelial Cells into Hepatocyte-like Cells. Cells. 2022 Jul 7;11(14):2138.	Hepatocytes	GFR Matrigel	GFR Matrigel	Human placental derived MSCs
17	33338746	Asadi M, Lotfi H, Salehi R, Mehdipour A, Zarghami N, Akbarzadeh A, et al. Hepatic cell-sheet fabrication of differentiated mesenchymal stem cells using decellularized extracellular matrix and thermoresponsive polymer. Biomed Pharmacother. 2021 Feb;134:111096.	Hepatocytes	No coating	Decellularised rat liver ECM	Human adipose-derived MSCs
18	36716645	Xue G, Zhang J, Wu L, Sun S, Wu H, Hou Y, et al. Differentiation of umbilical cord mesenchymal stem cells into hepatocytes with CYP450 metabolic enzyme activity induced by a liver injury microenvironment. Biochem Biophys Res Commun. 2023 Mar 5;647:47–54.	Hepatocytes	No coating	Decellularised rat liver ECM	Human umbilical cord MSCs
19	38379698	Kim W, Kim G. Engineered 3D liver-tissue model with minispheroids formed by a bioprinting process supported with in situ electrical stimulation. Bioact Mater. 2024 May;35:382–400.	Hepatocytes	No coating	No coating	Human adipose-derived MSCs
20	40313860	Lim JH, Kim DH, Lee J, Jung CR, Kang HM. Transdifferentiation of Integrin Beta 1 High+ Skin Progenitor Cells Into Functional Hepatocytes. Stem Cells Int. 2025;2025:8953305.	Hepatocytes	No coating	No coating	Human epidermal progenitor cells
21	36848084	Takashina T, Matsunaga A, Shimizu Y, Sakuma T, Okamura T, Matsuoka K, et al. Robust protein-based engineering of hepatocyte-like cells from human mesenchymal stem cells. Hepatol Commun. 2023 Mar 1;7(3):e0051.	Hepatocytes	No coating	No coating	Human umbilical cord MSCs
22	37072803	Garcia-Llorens G, Martínez-Sena T, Pareja E, Tolosa L, Castell JV, Bort R. A robust reprogramming strategy for generating hepatocyte-like cells usable in pharmaco-toxicological studies. Stem Cell Res Ther. 2023 Apr 18;14(1):94.	Hepatocytes	No coating	No coating	Human dermal fibroblasts
23	37552689	Heo SK, Yu HM, Kim DK, Seo HJ, Shin Y, Kim SA, et al. LIGHT (TNFSF14) promotes the differentiation of human bone marrow-derived mesenchymal stem cells into functional hepatocyte-like cells. PLoS One. 2023;18(8):e0289798.	Hepatocytes	No coating	No coating	Human bone marrow MSCs
24	10.2306/scienceasia1513-1874.2024.058	Nuttapasita N, Aungsuchawan S, Narakornsak S, Treebupachatsakul W, Markmee R, Thongdi N, et al. Differentiation potential of human amniotic fluid-derived mesenchymal stem cells into hepatocyte-like cells. ScienceAsia. 2024;50(3):1.	Hepatocytes	No coating	No coating	Human placental derived MSCs
25	40033232	Afshari A, Azarpira N, Pakbaz S. Differentiation of Wharton's jelly-derived mesenchymal stromal cells into hepatocyte-like cells using a refined method. BMC Mol Cell Biol. 2025 Mar 3;26(1):9.	Hepatocytes	No coating	No coating	Human umbilical cord MSCs
26	40001892	Lo Iacono M, Corrao S, Alberti G, Amico G, Timoneri F, Russo E, et al. Characterization and Proteomic Profiling of Hepatocyte-like Cells Derived from Human Wharton's Jelly Mesenchymal Stromal Cells: De Novo Expression of Liver-Specific Enzymes. Biology (Basel). 2025 Jan 24;14(2):124.	Hepatocytes	No coating	No coating	Human umbilical cord MSCs

27	39773036	Rashid S, Salim A, Naeem N, Haneef K. Synergistic Effects of Hydrogen Peroxide Preconditioning and Valproic Acid on Hepatic Differentiation of Mesenchymal Stem Cells. Curr Protein Pept Sci. 2025 Jan 3;	Hepatocytes	No coating	No coating	MSCs (not specified)
28	39656437	Wang X, Xu Y, Wang Y, Tang X, Zhou X, Lu W, et al. S-Nitrosylation of NOTCH1 Regulates Mesenchymal Stem Cells Differentiation Into Hepatocyte-Like Cells by Inhibiting Notch Signalling Pathway. J Cell Mol Med. 2024 Dec;28(23):e70274.	Hepatocytes	No coating	No coating	Human umbilical cord MSCs
29	39487286	Liu H, Bi X, Yang N, Zhang X, Fang B, Kusuman N, et al. Induced hepatocyte-like cells derived from adipose-derived stem cells alleviates liver injury in mice infected with Echinococcus Multilocularis. Sci Rep. 2024 Nov 1;14(1):26296.	Hepatocytes	No coating	No coating	Human adipose-derived MSCs
30	39461063	Jin Y, Zhang J, Chen X, Li F, Xue T, Yi K, et al. 3D printing incorporating gold nanozymes with mesenchymal stem cell-derived hepatic spheroids for acute liver failure treatment. Biomaterials. 2025 Apr;315:122895.	Hepatocytes	No coating	No coating	Human adipose-derived MSCs
31	39343956	Zhang Y, Hua M, Ma X, Li W, Cao Y, Han X, et al. Dipeptidyl peptidase-4 marks distinct subtypes of human adipose stromal/stem cells with different hepatocyte differentiation and immunoregulatory properties. Stem Cell Res Ther. 2024 Sep 29;15(1):338.	Hepatocytes	No coating	No coating	Human adipose-derived MSCs
32	38850132	Xu C, Fang X, Lu B, Song Y, Shu W, Lu Z, et al. Human umbilical cord mesenchymal stem cells alleviate fatty liver ischemia-reperfusion injury by activating autophagy through upregulation of IFNγ. Cell Biochem Funct. 2024 Jun;42(4):e4040.	Hepatocytes	No coating	No coating	Human umbilical cord MSCs
33	38529014	Chen F, Che Z, Liu Y, Luo P, Xiao L, Song Y, et al. Invigorating human MSCs for transplantation therapy via Nrf2/DKK1 co-stimulation in an acute-on-chronic liver failure mouse model. Gastroenterol Rep (Oxf). 2024;12:goae016.	Hepatocytes	No coating	No coating	Human adipose-derived MSCs
34	38442726	Yan M, Yao J, Xie Y, Jiang P, Yan J, Li X. Bioreactor-based stem cell therapy for liver fibrosis. Biofabrication. 2024 Mar 14;16(2).	Hepatocytes	No coating	No coating	Human umbilical cord MSCs
35	38323006	Jin M, Yi X, Zhu X, Hu W, Wang S, Chen Q, et al. Schisandrin B promotes hepatic differentiation from human umbilical cord mesenchymal stem cells. iScience. 2024 Feb 16;27(2):108912.	Hepatocytes	No coating	No coating	Human umbilical cord MSCs
36	37940517	Takasu C, Chen S, Gao L, Saito Y, Morine Y, Ikemoto T, et al. Role of Nrf2 signaling in development of hepatocyte-like cells. J Med Invest. 2023;70(3.4):343–9.	Hepatocytes	No coating	No coating	Human adipose-derived MSCs
37	37936499	Yu Y, Huang H, Ye J, Li Y, Xie R, Zeng L, et al. 3D Spheroids Facilitate Differentiation of Human Adipose-Derived Mesenchymal Stem Cells into Hepatocyte-Like Cells via p300-Mediated H3K56 Acetylation. Stem Cells Transl Med. 2024 Feb 14;13(2):151–65.	Hepatocytes	No coating	No coating	Human adipose-derived MSCs
38	37214257	Wei H, Li F, Xue T, Wang H, Ju E, Li M, et al. MicroRNA-122-functionalized DNA tetrahedron stimulate hepatic differentiation of human mesenchymal stem cells for acute liver failure therapy. Bioact Mater. 2023 Oct;28:50–60.	Hepatocytes	No coating	No coating	Human adipose-derived MSCs
39	37078751	Jin Y, Shi R, Qi T, Li Q, Chen C, Gao S, et al. Adipose-derived stem cells show hepatic differentiation potential and therapeutic effect in rats with acute liver failure. Acta Biochim Biophys Sin (Shanghai). 2023 Apr 20;55(4):601–12.	Hepatocytes	No coating	No coating	Human adipose-derived MSCs
40	36518486	Kantisin S, Chaisatra K, Hunsonti P, Parnlob V, Navasumrit P, Ruchirawat M. In utero arsenic exposure increases DNA damage and gene expression changes in umbilical cord mesenchymal stem cells (UC-MSCs) from newborns as well as in UC-MSC differentiated hepatocytes. Toxicol Rep. 2022;9:1728–41.	Hepatocytes	No coating	No coating	Human umbilical cord MSCs
41	36512201	Tarique S, Naeem N, Salim A, Ainuddin JA, Haneef K. The role of epigenetic modifiers in the hepatic differentiation of human umbilical cord derived mesenchymal stem cells. Biol Futur. 2022 Dec;73(4):495–502.	Hepatocytes	No coating	No coating	Human umbilical cord MSCs
42	35380478	Perera D, Soysa P, Wijeratne S. A Comparison of Mesenchymal Stem Cell-derived Hepatocyte-like Cells and HepG2 Cells for Use in Drug-Induced Liver Injury Studies. Altern Lab Anim. 2022 Mar;50(2):146–55.	Hepatocytes	No coating	No coating	MSCs (not specified)
43	35180101	Yin Y, Hu Z, Guan Z, Lv S, Wang Y, Su W, et al. Immunological characteristics of human umbilical cord mesenchymal stem cells after hepatogenic differentiation. Biomed Mater Eng. 2023;34(1):1–11.	Hepatocytes	No coating	No coating	Human umbilical cord MSCs
44	34975309	Jiang J, Xin J, Ding W, Shi D, Sun S, Guo B, et al. MicroRNA Profile of Human Bone Marrow Mesenchymal Stem Cells during Hepatic Differentiation and Therapy. Int J Med Sci. 2022;19(1):152–63.	Hepatocytes	No coating	No coating	Human bone marrow MSCs
45	34786159	Fatima A, Malick TS, Khan I, Ishaque A, Salim A. Effect of glycyrrhizic acid and 18β glycyrrhetinic acid on the differentiation of human umbilical cord-mesenchymal stem cells into hepatocytes. World J Stem Cells. 2021 Oct 26;13(10):1580–94.	Hepatocytes	No coating	No coating	Human umbilical cord MSCs
46	34362466	Fares AE, Gabr H, ShamsEldeen AM, Farghali HAM, Rizk MMSM, Mahmoud BE, et al. Implanted subcutaneous versus intraperitoneal bioscaffold seeded with hepatocyte-like cells: functional evaluation. Stem Cell Res Ther. 2021 Aug 6;12(1):441.	Hepatocytes	No coating	No coating	Human bone marrow MSCs
47	32551386	Allameh A, Ahmadi-Ashtiani HR, Maleki N. Glutathione-related inflammatory signature in hepatocytes differentiated from the progenitor mesenchymal stem cells. Heliyon. 2020 Jun;6(6):e04149.	Hepatocytes	No coating	No coating	Human bone marrow MSCs
48	40268456	Saito Y, Chen S, Ikemoto T, Teraoku H, Yamada S, Morine Y, et al. Farnesoid X receptor agonist accelerates ammonium metabolism of mesenchymal stem cell-derived hepatocyte-like cells. J Med Invest. 2025;72(1.2):54–9.	Hepatocytes	No coating	No coating	Human adipose-derived MSCs
49	37601454	Vazirzadeh M, Azarpira N, Vosough M, Ghaedi K. Galactosylation of rat natural scaffold for MSC differentiation into hepatocyte-like cells: A comparative analysis of 2D vs. 3D cell culture techniques. Biochem Biophys Rep. 2023 Sep;35:101503.	Hepatocytes	No coating	No coating	Human umbilical cord MSCs
50	21900788	Amer MEM, El-Sayed SZ, El-Kheir WA, Gabr H, Gomaa AA, El-Noomani N, et al. Clinical and laboratory evaluation of patients with end-stage liver cell failure injected with bone marrow-derived hepatocyte-like cells. Eur J Gastroenterol Hepatol. 2011 Oct;23(10):936–41.	Hepatocytes	No coating	No coating	Human bone marrow MSCs
51	38063365	Li F, Wei H, Jin Y, Xue T, Xu Y, Wang H, et al. Microfluidic Fabrication of MicroRNA-Induced Hepatocyte-Like Cells/Human Umbilical Vein Endothelial Cells-Laden Microgels for Acute Liver Failure Treatment. ACS Nano. 2023 Dec 26;17(24):25243–56.	Hepatocytes	No coating	No coating	Human umbilical cord MSCs
52	38380546	Zhang J, Chen X, Chai Y, Zhuo C, Xu Y, Xue T, et al. 3D Printing of a Vascularized Mini-Liver Based on the Size-Dependent Functional Enhancements of Cell Spheroids for Rescue of Liver Failure. Adv Sci (Weinh). 2024 May;11(17):e2309899.	Hepatocytes	No coating	Porcine liver derived ECM	Human adipose-derived MSCs
53	38143931	Campinoti S, Almeida B, Goudarzi N, Bencina S, Grundland Freile F, McQuitty C, et al. Rat liver extracellular matrix and perfusion bioreactor culture promote human amnion epithelial cell differentiation towards hepatocyte-like cells. J Tissue Eng. 2023;14:20417314231219813.	Hepatocytes	No coating	Rat liver ECM	Human placental derived MSCs
54	37575681	Mitani S, Onodera Y, Hosoda C, Takabayashi Y, Sakata A, Shima M, et al. Generation of functional liver sinusoidal endothelial-like cells from human bone marrow-derived mesenchymal stem cells. Regen Ther. 2023 Dec;24:274–81.	LSEC	Fibronectin	Fibronectin	Human bone marrow MSCs

55	40026618	Le QB, Ezhilarasu H, Chan WW, Patra AT, Murugan P, Venkatesh SA, et al. A platform for Bioengineering Tissue Membranes from cell spheroids. Mater Today Bio. 2025 Apr;31:101526.	Liver Organoids	No coating	Agarose	Human bone marrow MSCs
56	38749443	Bi G, Zhang X, Li W, Lu X, He X, Li Y, et al. Modeling alcohol-associated liver disease in humans using adipose stromal or stem cell-derived organoids. Cell Rep Methods. 2024 May 20;4(5):100778.	Liver Organoids	Matrigel	Collagen 1	Human adipose-derived MSCs
57	38534391	Chen S, Saito Y, Waki Y, Ikemoto T, Teraoku H, Yamada S, et al. Generation of Highly Functional Hepatocyte-like Organoids from Human Adipose-Derived Mesenchymal Stem Cells Cultured with Endothelial Cells. Cells. 2024 Mar 20;13(6):547.	Liver Organoids	No coating	Collagen 1	Human adipose-derived MSCs
58	33224947	Uchida Y, Ferdousi F, Zheng YW, Oda T, Isoda H. Global Gene Expression Profiling Reveals Isorhamnetin Induces Hepatic-Lineage Specific Differentiation in Human Amniotic Epithelial Cells. Front Cell Dev Biol. 2020;8:578036.	Liver Organoids	No coating	Lipidure	Human placental derived MSCs
59	37902572	Zhang Z, Wu Y, Xuan Z, Xu H, Yin S, Meng Z. Self-assembly of three-dimensional liver organoids: virtual reconstruction via endocytosed polymer dots for refactoring the fine structure. Biomater Sci. 2023 Dec 5;11(24):7867–83.	Liver Organoids	Wharton's jelly	Wharton's jelly	MSCs (not specified)
